# Ruthenium(ii)-catalyzed regioselective 1,6-conjugate addition of umpolung aldehydes as carbanion equivalents†

**DOI:** 10.1039/d1sc03732c

**Published:** 2021-11-29

**Authors:** Hyotaik Kang, Chao-Jun Li

**Affiliations:** Department of Chemistry, FRQNT Centre for Green Chemistry and Catalysis, McGill University 801 Sherbrooke St. W. Montréal Québec H3A 0B8 Canada cj.li@mcgill.ca

## Abstract

One of the most efficient and reliable approaches to construct C–C bonds involves the conjugate addition of carbon nucleophiles to electron-deficient ketones. Yet, 1,6-conjugate additions of extended conjugated systems largely remain underexplored due to difficulties in controlling the regioselectivity. Herein, we report umpolung aldehydes as carbanion equivalents for highly regioselective 1,6-conjugate addition reactions to unsaturated ketones, with preliminary studies of the enantioselective variant. The synergy of ruthenium(ii) catalyst and electron-rich, bidentate phosphine ligand is essential for the reactivity and selectivity under mild reaction conditions.

## Abstract 2

Highly regioselective 1,6-conjugate addition was developed using hydrazone as carbanion equivalent catalyzed by ruthenium under mild conditions.

## Graphical abstract

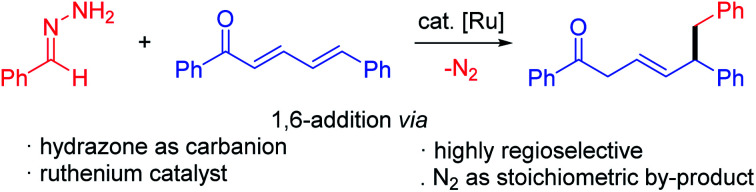

## Introduction

**Fig. 1:**
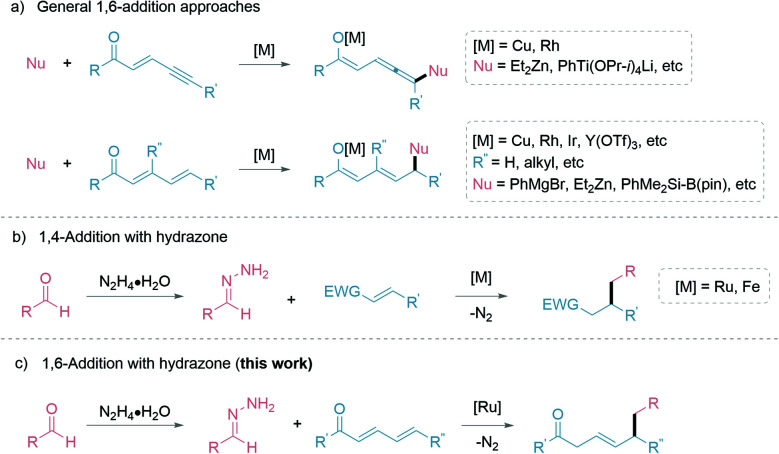
Strategies for various conjugate nucleophilic addition reactions.

Building molecular complexity *via* C–C bond formations is an invaluable tool in synthetic chemistry and often plays a pivotal role in reaction designs.^1^ In particular, the 1,4-addition of carbon nucleophiles to unsaturated carbonyl compounds is a common C–C bond formation strategy in the synthesis of fine chemicals and pharmaceuticals.^2–5^ However, controlling the regioselectivity of such nucleophiles can be challenging and has been a constant research interest.^6^ Additionally, this challenge is further elevated in the less common 1,6-conjugate addition, with comparably stagnant developments than the closely related 1,4-addition reactions. The complication is attributed to the presence of multiple electrophilic sites, resulting in different regioselectivities.^7^ Thus, strategies to adopt 1,6-addition includes: (1) modification of the electrophiles; and (2) adaptation of the carbon nucleophiles (Fig. 1a). The (1) modification of electrophiles involves using conjugated enynones^8–11^ and resorting to substitution at the β-position carbon to sterically suppress the 1,4-addition.^12–19^ Additionally, organocatalytic^20,21^ and Lewis-acid catalytic^22,23^ 1,6-additions allow for different activation pathways of the electrophiles. The (2) adaptation of carbon nucleophiles commonly utilizes soft organometallic reagents *via* transition-metal catalysis.^24–27^ Thus, several copper-catalyzed works are exemplified by Feringa,^28,29^ Hoveyda,^7,30^ and others.^31,32^ Alternative transition-metal catalyzed 1,6-additions have been developed by Hayashi with rhodium,^11^ cobalt,^33^ and iridium catalysts.^34,35^ Expansion of metal-catalyzed 1,6-additions to boryl and silyl groups are represented by the works of Lam,^36,37^ Liao^38^ and Newhouse.^39^ While these advancements are very significant, most involve stoichiometric use of organometallic nucleophiles, which inevitably leads to sizable metallic waste and poor atom economy. With our group's continuous pursuit in the umpolung of hydrazones as “soft” alkyl carbanions (Fig. 1b), herein we report the first example of ruthenium-catalyzed 1,6-addition of hydrazones as a simple and effective nucleophile with excellent regioselectivity (Fig. 1c). The application of hydrazones as nucleophiles is beneficial as their precursors, aldehyde moieties, are ubiquitous, commercially available, and can be renewably sourced.^40^ Furthermore, the formation of carbanion species by carbonyl umpolung generates a “soft” nucleophile through polarity inversion of the carbonyl carbon.^41^ To capitalize on the soft property of the nucleophile, we speculated a favourable, soft–soft interaction following the HSAB theory.^42^ For the nucleophile, such an interaction can be realized with a late transition-metal, ruthenium(ii)-catalyst. Likewise, the much softer δ-electrophilic position compared to the β-position favours the 1,6-addition over 1,4-addition.

**Fig. 2:**
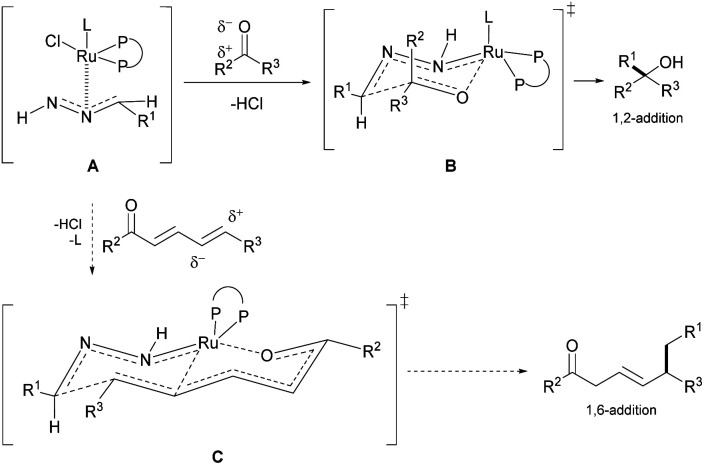
Proposed bicyclic TS (C) for the 1,6-conjugate addition and 6-membered ring chair-like TS (B) for 1,2-addition from the Ru-coordinated hydrazone intermediate (A).

Our group previously developed ruthenium-catalyzed 1,2-^43,44^ and 1,4-addition^45^ of hydrazones and postulated a possible six-membered ring transition-state from the *in situ* generated ruthenium-coordinated hydrazone intermediate (Fig. 2, A and B).^46^ We speculated that the polarized double bonds in the extended conjugated system could adopt a bicyclic transition state (Fig. 2, C), delivering the nucleophile at the 1,6-position selectively.

## Results and discussion

We started testing our hypothesis with the preformed hydrazone 2a from benzaldehyde and (*E*,*E*)-cinnamylideneacetophenone (3a) as the model substrates. The initial studies showed that the combination of bidentate phosphine ligand L1 (1.5 mol%) with [Ru(*p*-cymene)Cl_2_]_2_ (0.75 mol%), sodium carbonate (Na_2_CO_3_, 5.0 mol%), and caesium fluoride (CsF, 1.0 equiv.) in THF at 60 °C for 12 h gave a moderate yield of the desired 1,6-adduct 4a (Table 1, entry 1). Other inorganic bases such as KO*t*Bu, K_2_CO_3_, and Cs_2_CO_3_ were not as efficient (entry 2) as Na_2_CO_3_. Ir- and Rh-catalysts proved less effective (entries 3 and 4) and (PPh_3_)_4_RuCl_2_ performed better (entry 5). Evaluation of different ligands (entries 6–8) showed that the more π-acidic, phenyl substituted ligand, 1,2-bis(diphenylphosphino)ethane (L4, dppe), which could lead to stronger coordination and more polarization at the 6-position, gave both greater 1,6-regioselectivity and product yield compared to the alkyl and cyclohexyl counterparts, which were known previously beneficial for 1,2- and 1,4-additions. The additive CsF at 1.0 equiv. was critical for the reaction to proceed (entries 9 and 10), which was consistent with our previous reports.^43–45^ Conducting the reaction at 40 °C, 80 °C or using 2-Me-THF as solvent diminished the product yield (entries 11–13). The yield of 4a could be increased to 96% by prolonging the reaction time to 16 h (entries 14). It is noteworthy that the reaction under the optimized conditions gave 4a exclusively, with no 1,2- or 1,4-addition (4b) being observed. The entries with a reduced formation of 4a were mainly attributed to the recovered starting material 3a and the formation of azine 4c.

**Table 1:** Effects of reaction parameters^a^

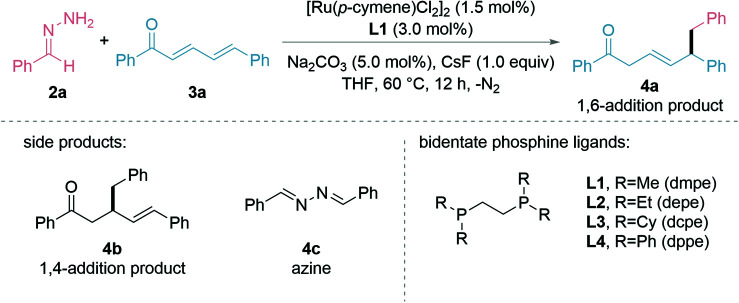		
Entry	Variation from standard conditions	Yield%
1	None	62
2	Other inorganic bases than Na_2_CO_3_ (KO*t*Bu, K_2_CO_3_, and Cs_2_CO_3_)	48–57
3	[Cp*IrCl_2_]_2_	15
4	[Cp*RhCl_2_]_2_	20
5	(PPh_3_)_4_RuCl_2_	66
6	(PPh_3_)_4_RuCl_2_, L2	51
7	(PPh_3_)_4_RuCl_2_, L3	53
8	(PPh_3_)_4_RuCl_2_, L4	86
9	(PPh_3_)_4_RuCl_2_, L4, no CsF	—
10	(PPh_3_)_4_RuCl_2_, L4, 1.5 equiv. CsF	78
11	(PPh_3_)_4_RuCl_2_, L4, 40 °C	60
12	(PPh_3_)_4_RuCl_2_, L4, 80 °C	72
13	(PPh_3_)_4_RuCl_2_, L4, 2-Me-THF	70
14	(PPh_3_)_4_RuCl_2_, L4, 16 h	96^b^

a Conditions: 0.2 mmol scale. 2a (1.25 M THF, 1.5 equiv.), 3a (0.20 mmol), catalyst (1.5 mol%), ligand (3.0 mol%), base (5.0 mol%), CsF (1.0 equiv.), THF, 60 °C, 12 h under N_2_ atmosphere. Yields by ^1^H NMR with dibromomethane as the internal standard.

b Isolated yield.

With the optimized reaction conditions in hand, the scope of the regioselective 1,6-conjugate addition was investigated (Table 2). In general, both electron-donating and withdrawing groups fared well under the reaction conditions. Alkyl, such as methyl and isopropyl substituted benzaldehyde hydrazones showed similar reactivities to the ether-substituted ones 5–12. Hydrazones generated from biphenyl-4-carboxaldehyde 14 and halobenzaldehydes 16–24 provided increased yields, possibly due to the stabilization of the *in situ* generated carbanion species by these substituents. The hydrazone bearing an aryl-amine functional group afforded a good yield of product 25, while the nitro-substituted one was less effective for generating 26. Delightfully, various heteroarene aldehydes, such as thiophene and pyridine-derived aldehydes (27–33) were all compatible with this reaction, albeit thiophene aldehydes and their derivatives (27–30) performed less efficiently. Surprisingly, the pyridine aldehyde and derivatives (31–33) gave good yields, despite being a possible chelating ligand in transition-metal catalysis.^47^ The utility of the reaction was further examined on linear and cyclic aliphatic aldehydes with increased base loading, generating the desired products 34–35 in lower yields. For the conjugated electrophile, both electron-withdrawing and donating groups on the aryl ketone provided the desired 1,6-addition products in moderate to good yields (36–43). The thiophene-derived heteroarene ketone also led to a moderate yield (44). It is noteworthy that ketones substituted at the γ-position, monoaryl substituted ketone, and a cyclopropyl ketone all generated the desired products in good yields (45–48). Importantly, a gram-scale synthesis of 4a (1.45 g, 89%) was performed to demonstrate the practicability of the reporting method.

**Table 2:** Nucleophilic and electrophilic substrate scope of the reaction^a^

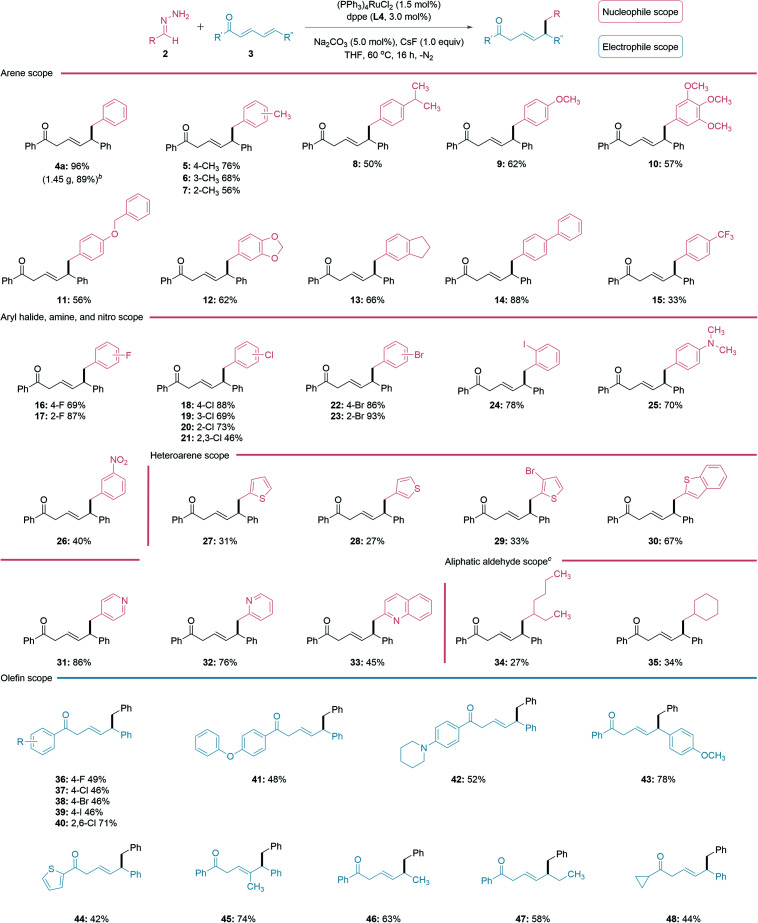

a General reaction conditions: 2 (1.25 M THF, 1.5 equiv.), 3 (0.20 mmol), (PPh_3_)_4_RuCl_2_ (1.5 mol%), dppe (L3, 3.0 mol%), Na_2_CO_3_ (5.0 mol%), CsF (1.0 equiv.), THF (100 μL) under N_2_ atmosphere at 60 °C for 16 h. The isolated yields are reported.

b Gram-scale reaction for 4a: 3a (5 mmol), (PPh_3_)_4_RuCl_2_ (0.75 mol%) and dppe (1.5 mol%) in THF (100 μL); isolated yield (1.45 g, 89%).

c Increased base loading to 1.2 equiv. for aliphatic aldehyde derivatives.

**Fig. 3:**
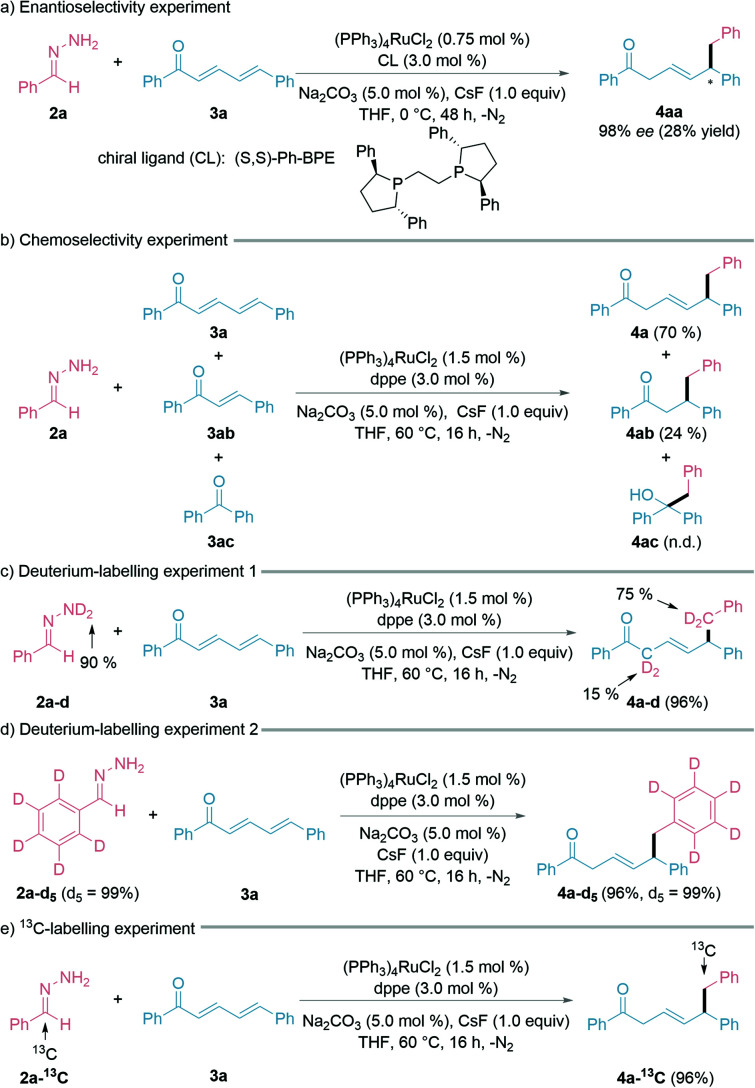
Enantioselectivity, chemoselectivity, and labelling experiments. See ESI† for more details.

To explore the possible enantioselectivity of this transformation, we examined various chiral ligands (ESI† for details). To our satisfaction, 98% ee was obtained with ligand (*S*,*S*)-Ph-BPE under the modified reaction conditions (0 °C, 48 h), albeit with a lower yield of the desired 1,6-product and the recovery of 3a (Fig. 3a). We then turned our attention to chemoselectivity, being an important challenge faced in modern synthetic chemistry.^48^ To study the chemoselectivity, we designed a competition experiment with a 1 : 1 : 1 mixture of (*E*,*E*)-cinnamylideneacetophenone (3a), (*E*)-chalcone (3ab), and benzophenone (3ac) (Fig. 3b). The nucleophilic 1,6-addition product 4a was much more favoured over the 1,4-addition product 4ab under the standard conditions, whereas the 1,2-addition product 4ac was not detected. A deuterium-labelling experiment using deuterated hydrazone (2a–d, 90% D) was conducted with 3a under the standard reaction conditions (Fig. 3c). The observation of H/D scrambling exclusively at the benzylic and α-positions suggested that the hydrazone acts as both the alkyl nucleophile and hydrogen donor. Deuterium-labelled aryl-D_5_ hydrazone retained all the deuterium on the aryl ring during the reaction (Fig. 3d). Finally, a ^13^C-labelled hydrazone led to the synthesis of a ^13^C-labelled 1,6-conjugate addition product by this method (Fig. 3e).

## Conclusions

In conclusion, we have developed a highly regioselective 1,6-conjugate addition to extended conjugated ketones. The combination of umpolung aldehydes as carbanions and ruthenium-catalyst with bidentate phosphine ligand (dppe) is important to the regioselectivity as both exhibit the key “soft” property. The reaction proceeds under mild reaction conditions with various functional group tolerance. Our efforts in the expansion of enantioselectivity, coupling partners, and synthetic applications are currently ongoing.

## Data availability

Data for all compounds in this manuscript are available in the ESI,† which includes general information, general procedures, experimental details, characterizations, and copies of ^1^H and ^13^C NMR spectra.

## Author contributions

CJL was involved in the conceptualization and supervision of the project, with funding acquisition and writing – reviewing and editing. HK performed the experimental investigations, formal analysis of data, and writing original draft.

## Conflicts of interest

There are no conflicts to declare.

## Supplementary Material

SC-013-D1SC03732C-s001
