# Supplementary material for: Ruthenium(ii)-catalyzed regioselective 1,6-conjugate addition of umpolung aldehydes as carbanion equivalents
Source: Chem Sci. 2021 Nov 29;13(1):118–22. doi: 10.1039/d1sc03732c (PMC8694324; doi:10.1039/d1sc03732c)
Supplement: SC-013-D1SC03732C-s001 [file SC-013-D1SC03732C-s001.pdf]

Electronic Supplementary Material (ESI) for Chemical Science.
This journal is © The Royal Society of Chemistry 2021

Supporting Information

Ruthenium(II)-Catalyzed Regioselective 1,6-Conjugate Addition of Umpolung Aldehydes as Carbanion Equivalents

Hyotaik Kang and Chao-Jun Li*

Department of Chemistry, FRQNT Centre for Green Chemistry and Catalysis, McGill University, 801 Sherbrooke St. W., Montreal, Quebec, Canada H3A 0B8

correspondence E-mail: cj.li@mcgill.ca

Table of Contents

1. General Experimental Information 3
2. Experimental Procedures 4
3. Optimization for the Reaction Conditions 6
4. Synthesis of Starting Materials 9
5. Chemoselective Competition Experiment 9
6. Deuterium-Labelling Studies 10
7. ^{13}C-Labelling Study 15
8. Product and Starting Material Characterization 17
9. ^{1}H and ^{13}C NMR Spectra 34
10. HPLC Chromatograph 93
11. References 94

1. General Experimental Information

Solvents: Tetrahydrofuran (THF) was taken from Pure Solvent MD-7 purification system from Innovative Technology in a dry round bottom flask with activated 5 Å molecular sieves beads activated in the oven at 380 °C for at least 12 h before use and purchased from Millipore Sigma. Solvents for filtration, transfers, and chromatography, were acetone (ACS grade), dichloromethane (CH_2Cl_2) (ACS grade), ethyl acetate (EtOAc) (ACS grade), hexane (Fisher, ACS grade), pentane (ACS grade), methanol (ACS grade), and chloroform (ACS grade).

Purification: All work-up and purification procedures were carried out with reagent-grade solvents. Analytical thin-layer chromatography (TLC) was performed using E. Merck silica gel 60 F_{254} pre-coated plates (0.25 mm). Flash column chromatography was performed with E. Merck silica gel P60 (40–63 µm particle size, 230–400 mesh) (SiO_2). Visualization was accomplished with UV light. Automated flash column chromatography was performed on Biotage Isolera™ Spektra Systems with ACI™.

Chemicals: In the model study, benzaldehyde (Millipore Sigma) was distilled before use. Other commercially available chemicals are purchased from Millipore Sigma, Alfa Aesar, Oakwood Chemicals, Thermo Fisher Scientific, and Santa Cruz Biotechnology and used without further purification.

NMR Spectroscopy: Nuclear magnetic resonance (^{1}H, ^{13}C, and ^{19}F) spectra were recorded on a Bruker AV500 equipped with a 60-position Sample Xpress sample changer (^{1}H, 500 MHz; ^{13}C, 125 MHz, ^{19}F 471 MHz). Chemical shifts are expressed in parts per million (ppm) units downfield from TMS, with the solvent residue peak as the chemical shift standard ($CDCl_3$: δ 7.26 ppm in ^{1}H NMR, δ 77.0 ppm in ^{13}C NMR). Data are reported as following: chemical shift, multiplicity (s = singlet, d = doublet, dd = doublet of doublets, t = triplet, td = triplet of doublets, q = quartet, quint = quintet, sext = sextet, sept = septet, m = multiplet, br = broad singlet), coupling constants *J* (Hz), and integration.

Mass Spectrometry: EI-MS was obtained from the Agilent GC-MS system. High-Resolution Mass (HRMS) spectra were performed by the McGill Chemistry Department Mass Spectrometry Facility and were recorded using electrospray ionization (ESI+) and/or atmospheric pressure chemical ionization APCI(+/-), performed either on an "Exactive Plus Orbitrap" ThermoScientific high-resolution accurate mass (HR/AM) FT mass spectrometer, or a Bruker Daltonics Maxis Impact quadrupole-time of flight (QTOF) mass spectrometer. Protonated molecular ions $(M+H)^+$ or sodium adducts $(M+Na)^+$ were used for empirical formula confirmation.

2. Experimental Procedures

2.1 Preparation of hydrazone solution

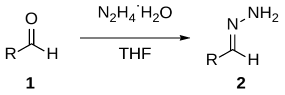

Hydrazone solution (1.25 M): Dry THF (1.0 mL) was added first into a small vial with a stir bar. Hydrazine monohydrate (2.3 mmol, 111.6 µL, 64-65 wt%) was added into the vial at 0 °C. Then, the corresponding aldehyde **1** (1.5 mmol) was added dropwise into the stirred solution and the mixture was stirred for 30 minutes. The solution was warmed to room temperature and stirred for an additional 3 h with the addition of a proper amount of anhydrous Na_2SO_4 for water removal.

2.2 General procedure for the regioselective 1,6-conjugate addition reaction

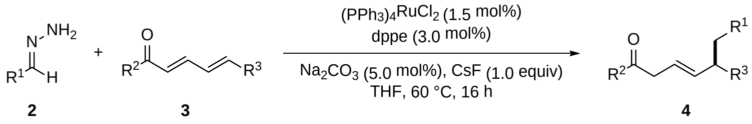

In a flame-dried or oven-dried V-shaped microwave reaction vial (2.0 mL) equipped with a Teflon-coated magnetic stirring bar, electron-deficient diene **3** (0.2 mmol), $(PPh_3)_4RuCl_2$ (0.003 mmol, 3.7 mg, 1.5 mol%), dppe (0.006 mmol, 2.4 mg, 3.0 mol%), and Na_2CO_3 (0.01 mmol, 1.1 mg, 5.0 mol%) were charged. The vial was sealed and transferred into the glovebox and charged with hydrazone solution **2** (1.25 M THF solution, 240 µL, 1.5 equiv), CsF (0.2 mmol, 30.4 mg, 1.0 equiv), and dry THF (100 µL). The vial was sealed with aluminum seals with PTFE-faced silicone septa, under an atmosphere of nitrogen. The mixture was stirred for 16 h under N_2 at 60 °C. After completion, the solution was filtered by celite with DCM as eluent, concentrated, and purified by silica gel thin-layer chromatography (TLC) to give the corresponding product.

2.3 General procedure for the enantioselective 1,6-addition reaction

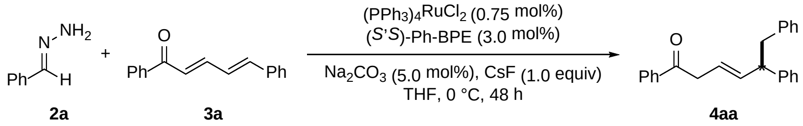

In a flame-dried or oven-dried V-shaped microwave reaction vial (2.0 mL) equipped with a Teflon-coated magnetic stirring bar, electron-deficient diene **3a** (0.2 mmol, 46.8 mg), $(PPh_3)_4RuCl_2$ (0.0015 mmol, 1.8 mg, 0.75 mol%), and Na_2CO_3 (0.01 mmol, 1.1 mg, 5.0 mol%) were charged. The vial was sealed and transferred into the glovebox and charged with hydrazone solution **2a** (1.25 M THF solution, 320 µL, 2.0 equiv), (*S*,*S*)-Ph-BPE (0.006 mmol, 3.0 mg, 3.0 mol%), CsF (0.2 mmol, 30.4 mg, 1.0 equiv), and dry THF (100 µL). The vial was sealed with aluminum seals with PTFE-faced silicone septa, under a nitrogen atmosphere. The mixture was stirred for 48 h under N_2 at 0 °C. After completion, the solution was filtered by celite with DCM as eluent, concentrated, and purified by silica gel TLC to give the corresponding product. The enantiomeric excess (ee %) was determined by HPLC.

2.4 Gram-scale synthesis of **4a**

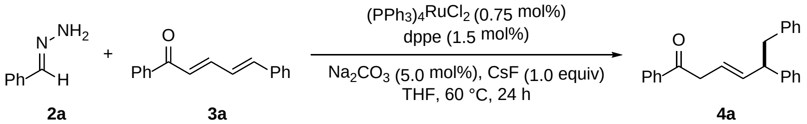

In a flame-dried screw-capped pressure reaction vial (15 mL) equipped with a Teflon-coated magnetic stirring bar, electron-deficient diene **3a** (5 mmol, 1.17 g), $(PPh_3)_4RuCl_2$ (0.0375 mmol, 45.9 mg, 0.75 mol%), dppe (0.075 mmol,

29.9 mg, 1.5 mol%), and Na_2CO_3 (0.25 mmol, 26.5 mg, 5.0 mol%) were charged. The vial was sealed and transferred into the glovebox, and charged with hydrazone solution **2a** (1.25 M THF solution, 6.0 mL, 1.5 equiv), CsF (5 mmol, 759.5 mg, 1.0 equiv), and dry THF (2.5 mL). The vial was sealed with a rubber O-ring attached screwcap under a nitrogen atmosphere. The mixture was stirred for 24 h under N_2 at 60 °C. After completion, the reaction mixture was then filtered by celite with DCM as eluent, concentrated, and purified by automated flash column chromatography (hexanes/ethyl acetate 9:1) to give the desired product **4a** as yellow crystal (1.45 g, 89% yield).

3. Optimization for the Reaction Conditions

Table S1. Evaluation of different bases[a]

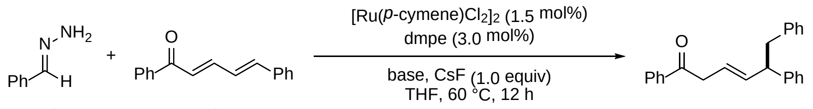

Entry	Base (5.0 mol%)	Yield (%)
1	KO*t*Bu	48
2	LiO*t*Bu	47
3	K_3PO_4	52
4	$CaCO_3$	58
5	K_2CO_3	57
6	Cs_2CO_3	55
7	Na_2CO_3	62
8	-	15

[a]Reaction conditions: **2a** (1.25 M THF solution, 1.5 equiv), **3a** (0.2 mmol), [Ru(*p*-cymene)Cl_2]$_2$ (0.003 mmol, 1.5 mol%), dmpe (0.006 mmol, 3.0 mol%), base (0.01 mmol, 5.0 mol%), CsF (0.2 mmol, 1.0 equiv), dry THF (100 µL), 60 °C, 12 h, and under N_2. Yields determined by crude ^{1}H NMR using mesitylene as an internal standard.

Table S2. Evaluation of catalyst[a]

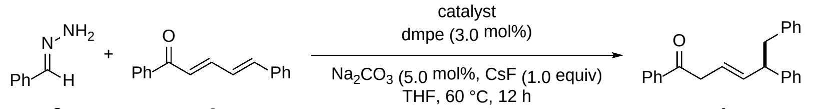

Entry	Catalyst (1.5 mol%)	Yield (%)
1	[Ru(*p*-cymene)Cl_2]$_2$	62
2	[Ru(COD)Cl_2]n	56
3	$(PPh_3)_4RuCl_2$	66
4	[Rh(nbd)Cl]$_2$	7
5	[Rh(COD)Cl]$_2$	7
6	[Cp*RhCl_2]$_2$	20
7	[Cp*IrCl_2]$_2$	15

[a]Reaction conditions: **2a** (1.25 M THF solution, 1.5 equiv), **3a** (0.2 mmol), catalyst (0.003 mmol, 1.5 mol%), dmpe (0.006 mmol, 3.0 mol%), Na_2CO_3 (0.01 mmol, 5.0 mol%), CsF (0.2 mmol, 1.0 equiv), dry THF (100 µL), 60 °C, 12 h, and under N_2. Yields determined by crude ^{1}H NMR using mesitylene as an internal standard.

Table S3. Evaluation of ligands[a]

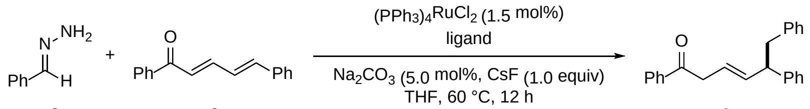

2a **3a** **4a**

Entry	Ligand (3.0 mol %)	Yield (%)
1	SL1	30
2	SL2	66
3	SL3	51
4	SL4	53
5	SL5	86
6	SL6	56
7	SL7	61
8	SL8	43
9	SL9	54

[a]Reaction conditions: **2a** (1.25 M THF solution, 1.5 equiv), **3a** (0.2 mmol), $(PPh_3)_4RuCl_2$ (0.003 mmol, 1.5 mol%), ligand (0.006 mmol, 3.0 mol%), Na_2CO_3 (0.01 mmol, 5.0 mol%), CsF (0.2 mmol, 1.0 equiv), dry THF (100 µL), 60 °C, 12 h, and under N_2. Yields determined by crude ^{1}H NMR using mesitylene as an internal standard.

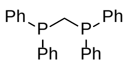

SL1 dppm
Bis(diphenylphosphino)methane

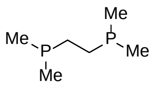

SL2 dmpe
1,2-Bis(dimethylphosphino)ethane

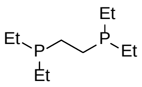

SL3 depe
1,2-Bis(diethylphosphino)ethane

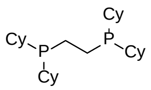

SL4 dcpe
1,2-Bis(dicyclohexylphosphino)ethane

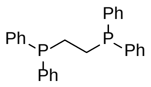

SL5 dppe
1,2-Bis(diphenylphosphino)ethane

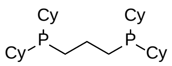

SL6 dcpp
1,3-Bis(dicyclohexylphosphino)propane

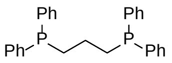

SL7 dppp
1,3-Bis(diphenylphosphino)propane

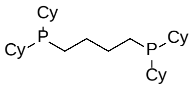

SL8 dcpb
1,4-Bis(dicyclohexylphosphino)butane

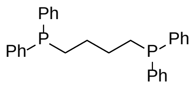

SL9 dppb
1,4-Bis(diphenylphosphino)butane

Figure S1. Ligands in Tables S3.

Table S4. Evaluation of different experimental parameters[a]

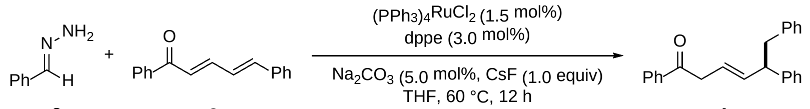

2a **3a** **4a**

Entry	Variation from conditions above	Yield (%)
1	none	86
2	10 h	70
3	16 h	96
4	no CsF	n.d.
5	CsF (0.50 equiv)	75
6	CsF (1.50 equiv)	78
7	40 °C	60
8	80 °C	72
9	diethyl ether	60
10	1,4-dioxane	65
11	2-methyltetrahydrofuran	70

[a]Reaction conditions: **2a** (1.25 M THF solution, 1.5 equiv), **3a** (0.2 mmol), $(PPh_3)_4RuCl_2$ (0.003 mmol, 1.5 mol%), dmpe (0.006 mmol, 3.0 mol%), Na_2CO_3 (0.01 mmol, 5.0 mol%), CsF (0.2 mmol, 1.0 equiv), dry THF (100 µL), 60 °C, 12 h, and under N_2. Yields determined by crude ^1H NMR using mesitylene as an internal standard.

Table S5. Evaluation of asymmetric reaction conditions[a]

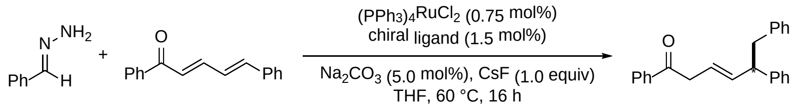

2a **3a** **4aa**

Entry	Chiral ligand	*ee* (%)	Yield (%)
1	SCL1	0	51
2	SCL2	0	43
3	SCL3	0	54
4	SCL4	0	28
5	SCL5	0	46
6	SCL6	0	55
7	SCL7	0	91
8	SCL8	0	52
9	SCL9	19	59
10[b]	SCL9	44	55
11[c]	SCL9	98	28

[a]Reaction conditions: **2a** (1.25 M THF solution, 1.5 equiv), **3a** (0.2 mmol), $(PPh_3)_4RuCl_2$ (0.0015 mmol, 0.75 mol%), CL (0.003 mmol, 1.5 mol%), Na_2CO_3 (0.01 mmol, 5.0 mol%), CsF (0.2 mmol, 1.0 equiv), dry THF (100 µL), 60 °C, 16 h, and under N_2. Yields determined by crude ^1H NMR using mesitylene as an internal standard.[b]40 °C. [c]0 °C, 48 h, **2a** (1.25 M THF solution, 2.0 equiv), SCL9 3.0 mol%.

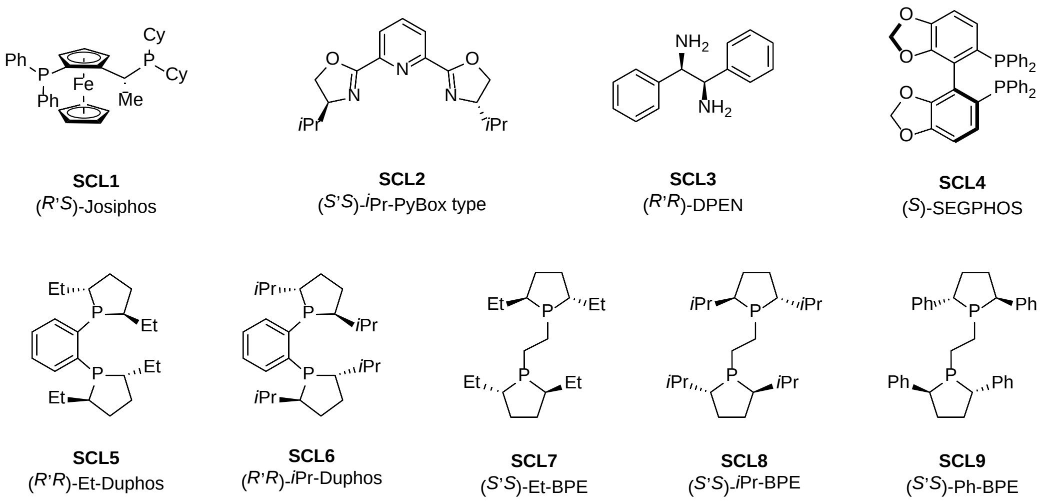

Figure S2. Chiral ligands in Table S5.

4. Synthesis of Starting Materials

The general procedures are demonstrated by the synthesis of (*E*,*E*)-cinnamylideneacetophenone, and other conjugated ketones are synthesized in similar methods with the appropriate substitution of acetophenone or cinnamaldehyde. The starting materials **3l** and **3m** are synthesized following a different method according to reported literature.[3]

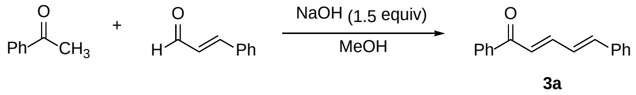

Synthesis of (*E*,*E*)-cinnamylideneacetophenone (**3a**): The methods used were according to reported literature.[1,2] To a 50 mL round-bottom flask charged with a magnetic stir bar, acetophenone (4.0 mmol, 466.6 µL), 20 mL methanol, and sodium hydroxide (6 mmol, 240 mg, 1.5 equiv) were slowly added and cooled to 0 °C. After cooling the solution to 0 °C, cinnamaldehyde (4.8 mmol, 604.1 µL, 1.2 equiv) was added dropwise under constant stirring. The mixture was brought to room temperature and stirred for an additional 20 h. After completion of the reaction, the mixture was quenched by water and dilute hydrochloric acid (1 M). The obtained precipitate was filtered, washed with cooled methanol, and purified by silica gel chromatography. The purity of the product was determined by ^{1}H NMR and recrystallized from ethanol if needed. The **3a** was isolated as yellow crystals (80% yield) and shown to match analytical data to those previously reported.[1,2]

5. Chemoselective Competition Experiment

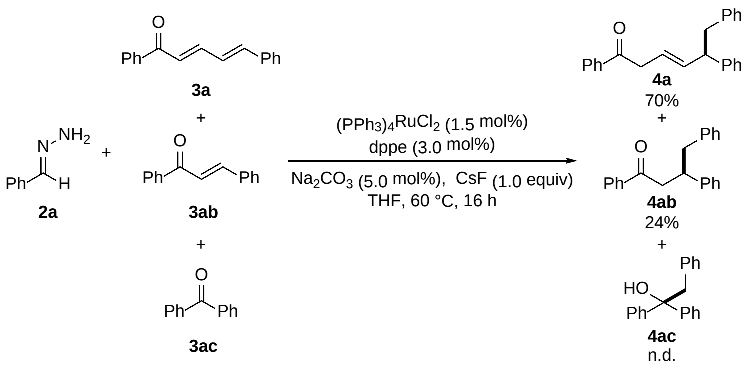

A competition experiment was designed with a 1:1:1 mixture of (*E*,*E*)-cinnamylideneacetophenone (**3a**), (*E*)-chalcone (**3ab**), and benzophenone (**3ac**). In a flame-dried or oven-dried V-shaped microwave reaction vial (2 mL) equipped with a Teflon-coated magnetic stirring bar, **3a** (0.1 mmol, 23.4 mg, 1.0 equiv), **3ab** (0.1 mmol,20.8 mg, 1.0 equiv), **3ac** (0.1 mmol, 18.2 mg, 1.0 equiv), $(PPh_3)_4RuCl_2$ (0.0015 mmol, 1.8 mg, 1.5 mol%,), dppe (0.003 mmol, 1.2 mg, 3.0 mol%), and Na_2CO_3 (0.005 mmol, 0.5 mg, 5.0 mol%) were charged. The vial was transferred into the glovebox and charged with hydrazone solution (1.25M, 80 µL, 1.0 equiv), CsF (0.1 mmol, 15.2 mg, 1.0 equiv), and 100 µL of dry THF and then sealed with a PTEF-faced silicone septum under N_2 atmosphere. The reaction system was then heated to 60 °C in an oil bath. After completion, the solution was filtered by celite, concentrated, and the yield was determined by ^{1}H NMR with dibromomethane as the internal standard (**4a** 70%, **4ab** 24%, and **4ac** n.d.), we observed high chemoselectivity for the 1,6-addition.

6. Deuterium-Labelling Studies

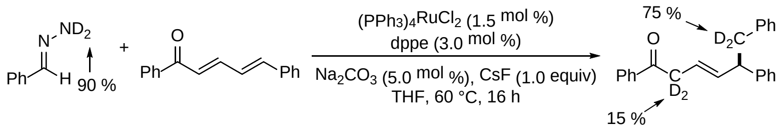

2a-d **3a** **4a-d** (96%)

The deuterated hydrazone **2a-d** was synthesized according to the literature.[4] Hydrazone **2-d** (90% D): **^{1}H NMR** (500 MHz, $CDCl_3$) δ 7.75 (s, 1H), 7.56 – 7.54 (m, 2H), 7.37 – 7.33 (m, 2H), 7.32 – 7.28 (m, 1H), 5.49 (br, 0.20H). **^{2}H NMR** (77 MHz, $CDCl_3$) δ 5.45

The other operations were following the general procedures at 0.1 mmol scale. **4a-d** (31.2 mg, 96%). Isolated by preparative TLC. Percent deuterium (% D) incorporation was depicted as the amount of deuterium in place of the combined hydrogen atoms at that site. **4a-d**: **^{1}H NMR** (500 MHz, $CDCl_3$): 7.89 – 7.85 (m, 2H), 7.56 – 7.53 (m, 1H), 7.48 – 7.44 (m, 2H), 7.30 – 7.27 (m, 5H), 7.22 – 7.17 (m, 5H), 6.33 – 6.29 (m, 1H), 6.18 – 6.13 (m, 1H), 3.26 (dt, *J* = 8.1, 6.7 Hz, 1H), 3.10 – 3.04 (m, 1.70H), 2.90 – 2.83 (m, 0.50H). **HRMS** (ESI +ve, *m/z*) Calculated for $C_{24}H_{20}D_2ONa$ $[M+Na]^+$ 351.1688; found: 351.1685. Deuterium incorporation was determined by ^{1}H NMR.

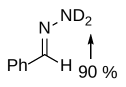

2a-d

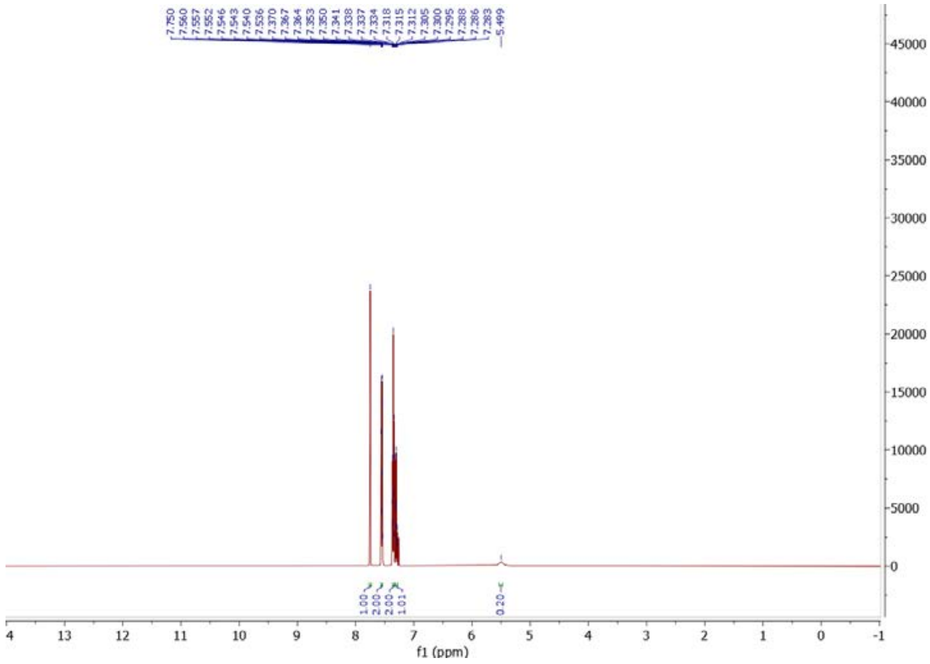

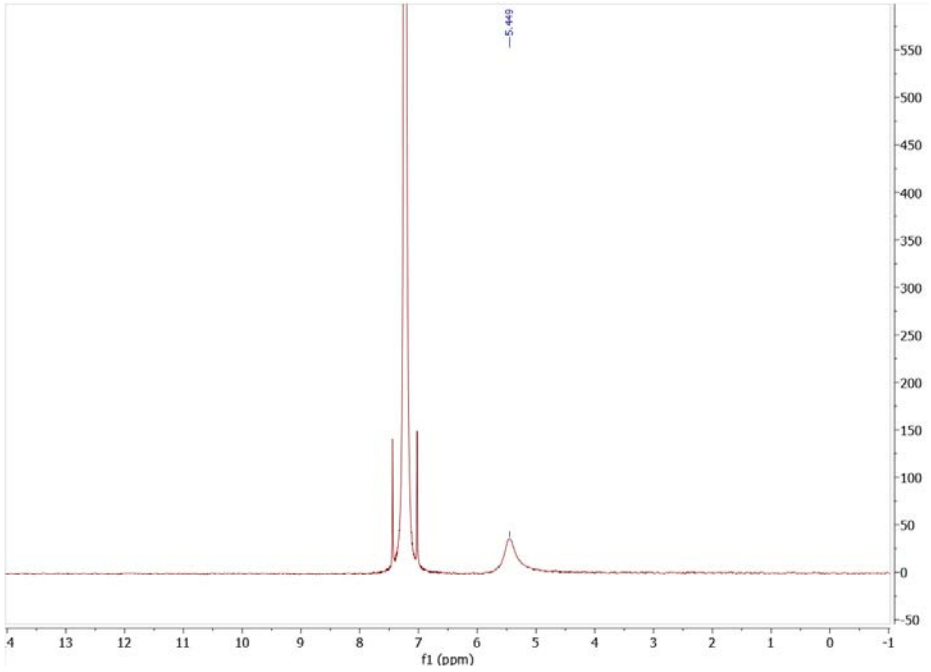

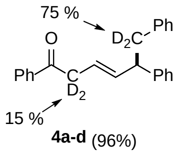

4a-d (96%)

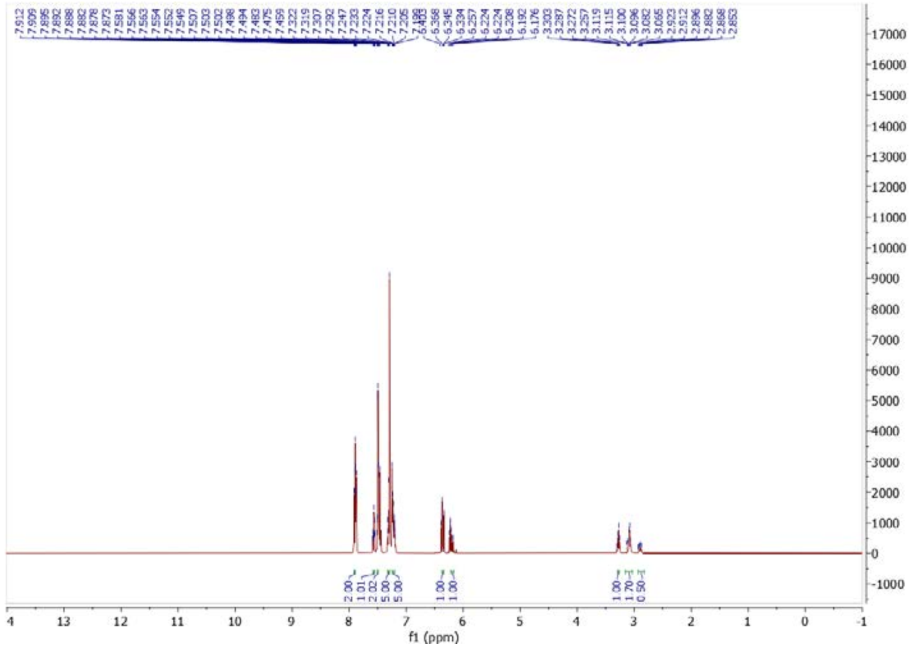

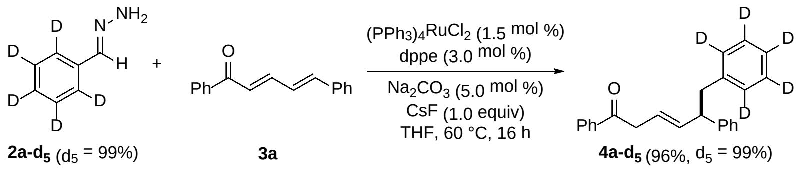

The deuterated hydrazone **2a-d₅** was synthesized according to the literature.[4] Hydrazone **2-d₅** (99% D): **^{1}H NMR** (500 MHz, $CDCl_3$) δ 7.90 (s, 0.01H), 7.75 (s, 1H), 7.59 (s, 0.01H), 7.36 (s, 0.01H), 5.52 (br, 2H). **^{2}H NMR** (77 MHz, $CDCl_3$) δ 7.89, 7.56, 7.38.

The other operations were following the general procedures at 0.1 mmol scale. **4a-d₅** (31.8 mg, 96%). Isolated by preparative TLC. Percent deuterium (% D) incorporation was depicted as the amount of deuterium in place of the combined hydrogen atoms at that site. **4a-d₅**: **^{1}H NMR** (500 MHz, $CDCl_3$): 7.87 (dd, *J* = 8.4, 1.3 Hz, 2H), 7.55 – 7.51 (m, 1H), 7.44 – 7.41 (m, 2H), 7.26 – 7.25 (m, 4H), 7.19 – 7.16 (m, 1H), 6.29 (dd, *J* = 15.9, 8.0 Hz, 1H), 6.15 (dt, *J* = 15.9, 7.2 Hz, 1H), 3.26 (dt, *J* = 8.1, 6.7 Hz, 1H), 3.06 (d, *J* = 7.2 Hz, 2H), 2.86 (d, *J* = 6.7 Hz, 2H). **HRMS** (ESI +ve, *m/z*) Calculated for $C_{24}H_{17}D_5ONa$ $[M+Na]^+$ 354.1877; found: 354.1883. Deuterium incorporation was determined by ^{1}H NMR.

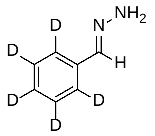

2a-d₅ (99% D)

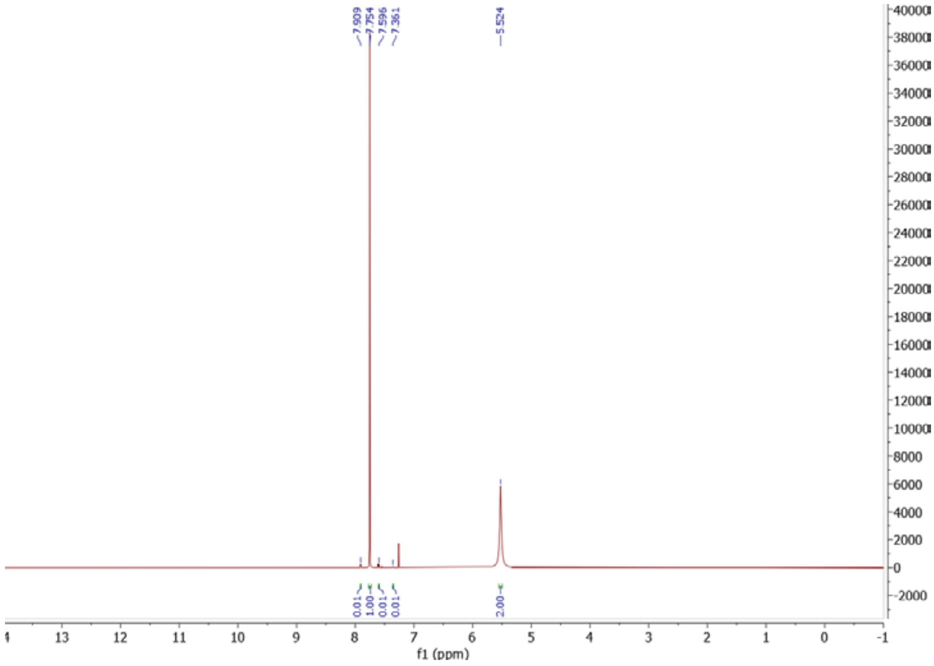

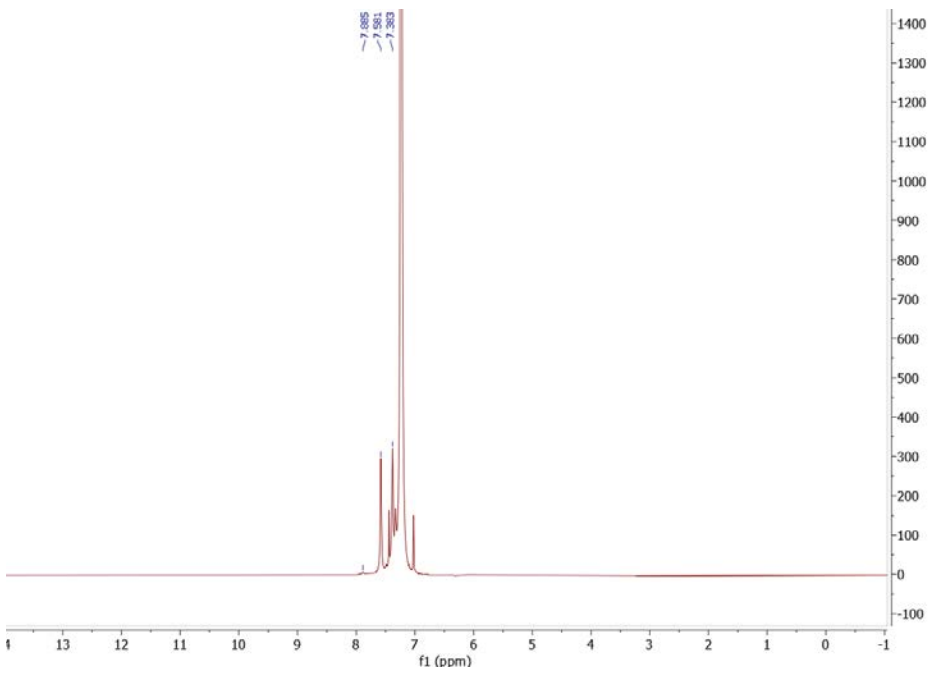

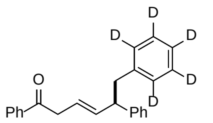

4a-d$_5$ (96%, d$_5$ = 99%)

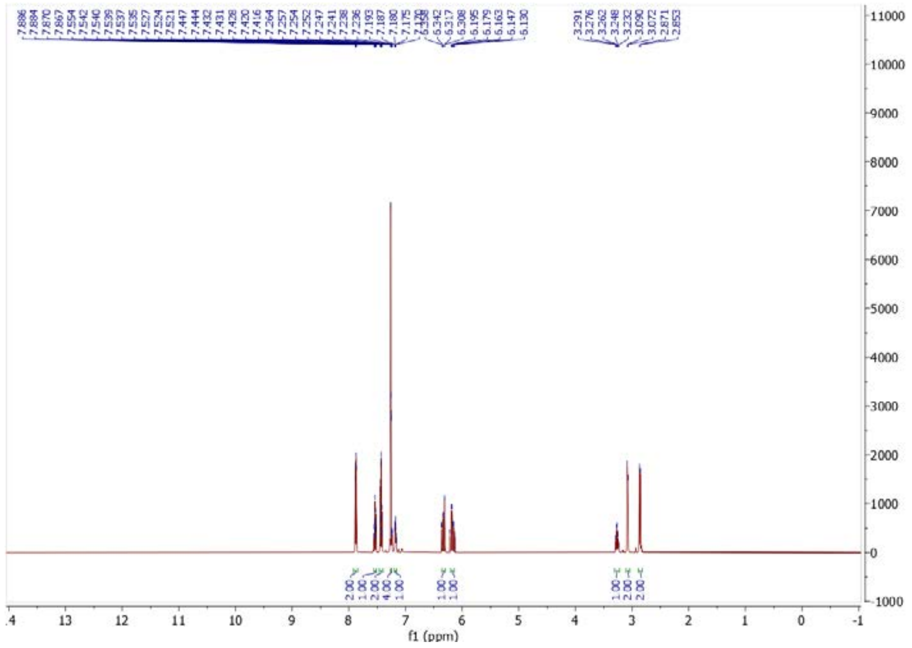

7. ^{13}C-Labelling Study

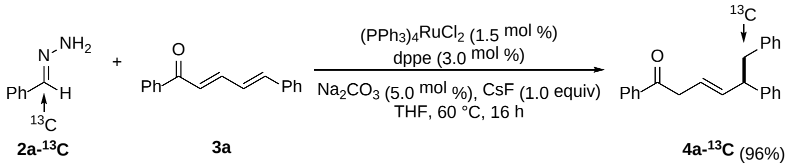

Hydrazone **2a-^{13}C** : **^{1}H NMR** (500 MHz, $CDCl_3$) δ 7.75 (d, *J* = 156 Hz , 1H), 7.59 – 7.53 (m, 2H), 7.37 – 7.33 (m, 2H), 7.32 – 7.27 (m, 1H), 5.52 (br, 2H).

The other operations were following the general procedures at 0.1 mmol scale. **4a-^{13}C** (31.2 mg, 96%). Isolated by preparative TLC. **4a-^{13}C**: **^{1}H NMR** (500 MHz, $CDCl_3$): 7.88 (dd, *J* = 8.4, 1.3 Hz, 2H), 7.56 – 7.52 (m, 1H), 7.45 – 7.42 (m, 2H), 7.35 – 7.27 (m, 5H), 7.24 – 7.16 (m, 5H), 6.29 (dd, *J* = 15.9, 8.1 Hz, 1H), 6.19 – 6.15 (m, 1H), 3.26 (dt, *J* = 8.1, 6.7 Hz, 1H), 3.09 (d, *J* = 7.2 Hz, 2H), 3.00 – 2.72 (m, 2H).

^{13}C NMR (126 MHz, $CDCl_3$) δ 199.2, 139.4, 137.4, 137.2, 132.9, 132.7, 130.2, 129.4, 129.4, 128.5, 128.4, 128.4, 128.3, 128.0, 127.1, 126.2, 126.1, 42.9, 41.4, 40.4. **HRMS** (ESI +ve, *m/z*) Calculated for $C_{23}{}^{13}CH_{22}ONa$ $[M+Na]^+$ 350.1596; found: 350.1603.

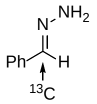

2a-^{13}C

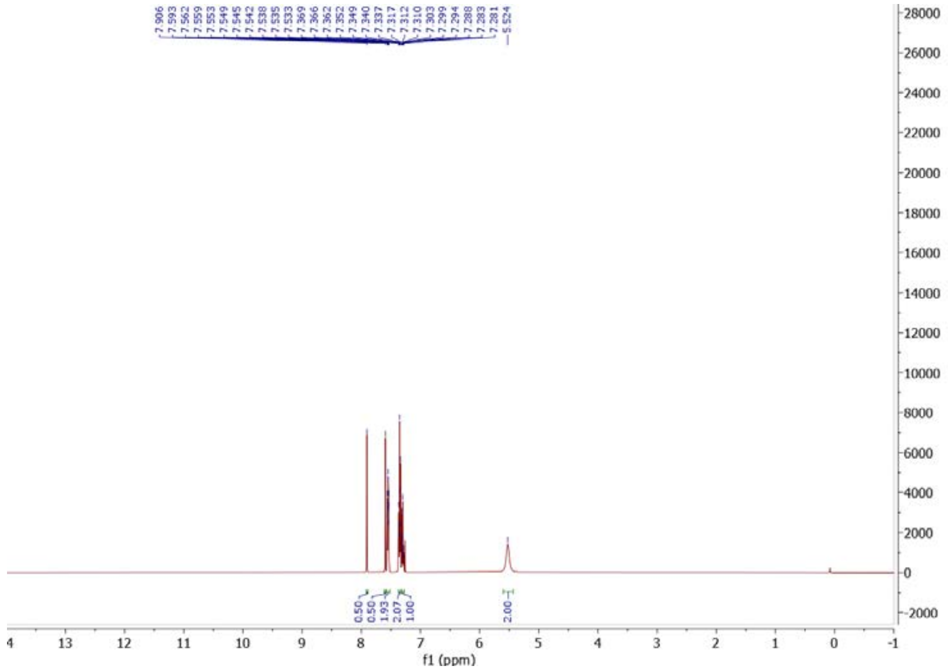

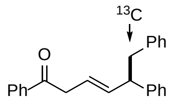

4a-^{13}C

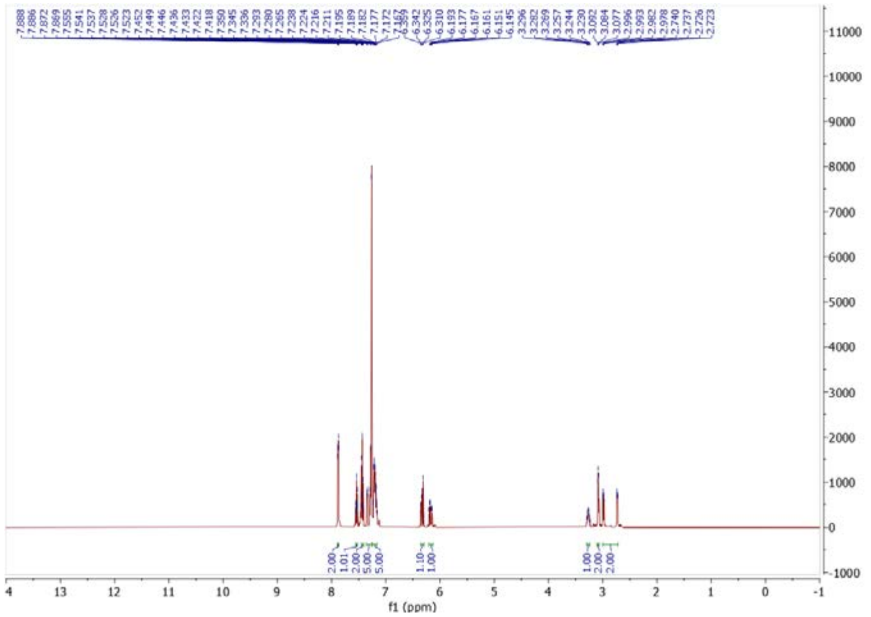

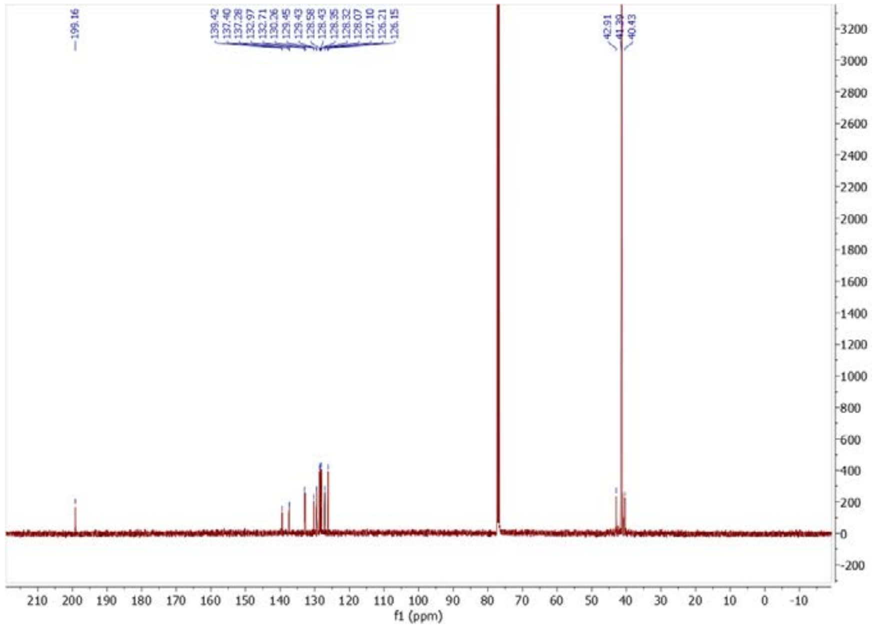

8. Product and Starting Material Characterization

8.1 1,6-conjugate addition electrophilic substrates

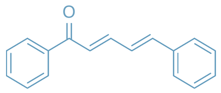

3a

(2*E*,4*E*)-1,5-diphenylpenta-2,4-dien-1-one (**3a**)
Appearance: Yellow solid
^{1}H NMR (500 MHz, $CDCl_3$) δ 8.03 – 7.98 (m, 2H), 7.65 – 7.57 (m, 2H), 7.53 – 7.49 (m, 4H), 7.41 – 7.33 (m, 3H), 7.13 – 7.08 (m, 1H), 7.07 – 7.03 (m, 2H).
^{13}C NMR (126 MHz, $CDCl_3$) δ 190.5, 144.8, 141.9, 138.2, 136.1, 132.6, 129.2, 128.8, 128.6, 128.4, 127.3, 126.9, 125.4.
HRMS (APCI +ve, *m/z*) Calculated for $C_{17}H_{15}O$ $[M+H]^+$ 235.1117; found: 235.1119.

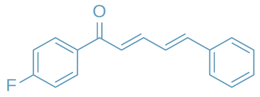

3b

(2*E*,4*E*)-1-(4-fluorophenyl)-5-phenylpenta-2,4-dien-1-one (**3b**)
Appearance: Yellow solid
^{1}H NMR (500 MHz, $CDCl_3$) δ 8.07 – 8.00 (m, 2H), 7.66 – 7.61 (m, 1H), 7.53 – 7.51 (m, 2H), 7.42 – 7.34 (m, 3H), 7.21 – 7.15 (m, 2H), 7.11 – 7.03 (m, 3H).

^{13}C NMR (126 MHz, $CDCl_3$) δ 188.8, 165.5 (d, *J* = 254.1 Hz), 145.1, 142.2, 136.1, 134.5 (d, *J* = 3.0 Hz), 131.0 (d, *J* = 9.1 Hz), 129.3, 128.9, 127.4, 126.8, 124.9, 115.7 (d, *J* = 22.3 Hz).
HRMS (ESI +ve, *m/z*) Calculated for $C_{17}H_{13}FONa$ $[M+Na]^+$ 275.0847; found: 275.0843.

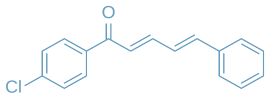

3c

(2*E*,4*E*)-1-(4-chlorophenyl)-5-phenylpenta-2,4-dien-1-one (**3c**)
Appearance: Yellow solid
^{1}H NMR (500 MHz, $CDCl_3$) δ 8.00 – 7.90 (m, 2H), 7.85 – 7.74 (m, 1H), 7.66 – 7.61 (m, 1H), 7.55 – 7.51 (m, 2H), 7.42 – 7.37 (m, 4H), 7.06 – 7.05 (m, 2H), 6.63 – 6.44 (m, 1H).
^{13}C NMR (126 MHz, $CDCl_3$) δ 189.1, 145.3, 142.4, 139.0, 136.5, 136.0, 129.8, 129.3, 128.9, 128.9, 127.3, 126.7, 124.8.
HRMS (APCI +ve, *m/z*) Calculated for $C_{17}H_{14}ClO$ $[M+H]^+$ 269.0725; found: 269.0728.

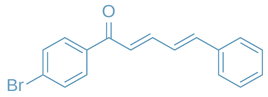

3d

(2*E*,4*E*)-1-(4-bromophenyl)-5-phenylpenta-2,4-dien-1-one (**3d**)
Appearance: Yellow solid
^{1}H NMR (500 MHz, $CDCl_3$) δ 7.9 – 7.83 (m, 2H), 7.72 – 7.70 (m, 1H), 7.67 – 7.65 (m, 2H), 7.57 – 7.53 (m, 2H), 7.43 – 7.37 (m, 4H), 7.08 – 7.05 (m, 1H), 6.58 – 6.51 (m, 1H).
^{13}C NMR (126 MHz, $CDCl_3$) δ 189.3, 145.4, 142.4, 136.9, 136.0, 131.9, 129.9, 129.3, 128.9, 127.7, 127.3, 126.7, 124.7.
HRMS (APCI +ve, *m/z*) Calculated for $C_{17}H_{14}BrO$ $[M+H]^+$ 313.0223; found: 313.0220.

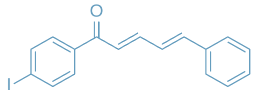

3e

(2*E*,4*E*)-1-(4-iodophenyl)-5-phenylpenta-2,4-dien-1-one (**3e**)
Appearance: Yellow solid
^{1}H NMR (500 MHz, $CDCl_3$) δ 7.92 – 7.84 (m, 2H), 7.74 – 7.69 (m, 2H), 7.66 – 7.61 (m, 1H), 7.56 – 7.52 (m, 2H), 7.47 – 7.29 (m, 4H), 7.08 – 7.01 (m, 2H).
^{13}C NMR (126 MHz, $CDCl_3$) δ 189.6, 145.4, 142.4, 137.9, 137.5, 136.0, 129.8, 129.3, 128.9, 127.3, 126.8, 124.7, 100.4.
HRMS (ESI +ve, *m/z*) Calculated for $C_{17}H_{13}IONa$ $[M+Na]^+$ 382.9903; found: 382.9902.

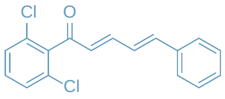

3f

(2*E*,4*E*)-1-(2,6-dichlorophenyl)-5-phenylpenta-2,4-dien-1-one (**3f**)
Appearance: Yellow solid
^{1}H NMR (500 MHz, $CDCl_3$) δ 7.50 – 7.48 (m, 2H), 7.41 – 7.31 (m, 6H), 7.08 – 6.92 (m, 3H), 6.62 – 6.42 (m, 1H).
^{13}C NMR (126 MHz, $CDCl_3$) δ 192.7, 147.7, 143.0, 138.0, 135.7, 131.9, 130.5, 129.7, 129.6, 128.9, 128.2, 127.5, 126.5.
HRMS (ESI +ve, *m/z*) Calculated for $C_{17}H_{12}Cl_2ONa$ $[M+Na]^+$ 325.0167; found: 325.0157.

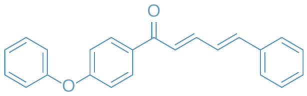

3g

(2*E*,4*E*)-1-(4-phenoxyphenyl)-5-phenylpenta-2,4-dien-1-one (**3g**)
Appearance: Yellow solid
^{1}H NMR (500 MHz, $CDCl_3$) δ 8.09 – 7.95 (m, 2H), 7.66 – 7.61 (m, 1H), 7.59 – 7.47 (m, 2H), 7.47 – 7.32 (m, 5H), 7.25 – 7.22 (m, 1H), 7.22 – 6.90 (m, 7H).
^{13}C NMR (126 MHz, $CDCl_3$) δ 188.8, 161.7, 155.6, 144.4, 141.7, 136.2, 132.8, 130.7, 130.1, 129.2, 128.9, 127.3, 127.0, 125.1, 124.6, 120.1, 117.4.
HRMS (APCI +ve, *m/z*) Calculated for $C_{23}H_{19}O_2$ $[M+H]^+$ 327.1380; found: 327.1367.

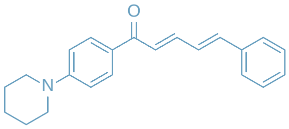

3h

(2*E*,4*E*)-5-phenyl-1-(4-(piperidin-1-yl)phenyl)penta-2,4-dien-1-one (**3h**)
Appearance: Yellow solid
^{1}H NMR (500 MHz, $CDCl_3$) δ 7.99 – 7.94 (m, 2H), 7.63 – 7.58 (m, 1H), 7.53 – 7.51 (m, 2H), 7.41 – 7.32 (m, 3H), 7.19 – 6.88 (m, 5H), 3.42 – 3.40 (m, 4H), 1.69 – 1.61 (m, 6H).
^{13}C NMR (126 MHz, $CDCl_3$) δ 187.8, 154.3, 142.8, 140.5, 136.4, 130.6, 128.8, 128.8, 127.3, 127.3, 127.1, 125.6, 113.3, 48.6, 25.4, 24.3.
HRMS (APCI +ve, *m/z*) Calculated for $C_{22}H_{24}NO$ $[M+H]^+$ 318.1780; found: 318.1790.

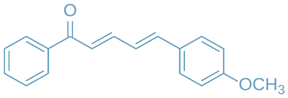

3i

(2*E*,4*E*)-5-(4-methoxyphenyl)-1-phenylpenta-2,4-dien-1-one (**3i**)
Appearance: Yellow solid
^{1}H NMR (500 MHz, $CDCl_3$) δ 8.02 – 7.97 (m, 2H), 7.65 – 7.56 (m, 2H), 7.52 – 7.45 (m, 4H), 7.09 – 6.90 (m, 5H), 3.84 (s, 3H).
^{13}C NMR (126 MHz, $CDCl_3$) δ 190.5, 160.6, 145.4, 141.8, 138.4, 132.5, 128.9, 128.8, 128.5, 128.3, 124.8, 124.2, 114.3, 55.3.
HRMS (ESI +ve, *m/z*) Calculated for $C_{18}H_{16}ONa$ $[M+Na]^+$ 271.1093; found: 271.1105.

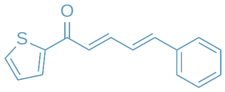

3j

(2*E*,4*E*)-5-phenyl-1-(thiophen-2-yl)penta-2,4-dien-1-one (**3j**)
Appearance: Yellow solid
^{1}H NMR (500 MHz, $CDCl_3$) δ 7.83 (dd, *J* = 3.8, 1.2 Hz, 1H), 7.70 – 7.64 (m, 2H), 7.55 – 7.51 (m, 2H), 7.43 – 7.34 (m, 3H), 7.21 – 7.19 (m, 1H), 7.09-7.00 (m, 3H).
^{13}C NMR (126 MHz, $CDCl_3$) δ 182.1, 145.7, 144.0, 142.1, 136.1, 133.6, 131.5, 129.2, 128.8, 128.2, 127.3, 126.7, 125.0.
HRMS (APCI +ve, *m/z*) calculated for $C_{15}H_{13}OS$ $[M+H]^+$ 241,0682m; found: 241.0672.

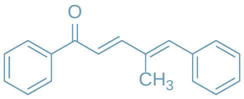

3k

(2*E*,4*E*)-4-methyl-1,5-diphenylpenta-2,4-dien-1-one (**3k**)
Appearance: Yellow solid
^{1}H NMR (500 MHz, $CDCl_3$) δ 8.03 – 8.02 (m, 2H), 7.68 (d, *J* = 15.3 Hz, 1H), 7.62 – 7.59 (m, 1H), 7.54 – 7.51 (m, 2H), 7.44 – 7.40 (m, 4H), 7.35 – 7.32 (m, 1H), 7.09 (d, *J* = 15.3 Hz, 1H), 7.00 (s, 1H), 2.20 (s, 3H).
^{13}C NMR (126 MHz, $CDCl_3$) δ 190.8, 150.2, 140.5, 138.5, 136.7, 134.7, 132.6, 129.5, 128.5, 128.4, 128.4, 127.9, 121.5, 13.9.
HRMS (ESI +ve, *m/z*) calculated for $C_{18}H_{16}ONa$ $[M+Na]^+$ 271.1093; found: 271.1105.

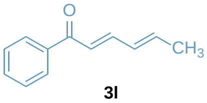

3l

(2*E*,4*E*)-1-phenylhexa-2,4-dien-1-one (**3l**)
Appearance: Yellow solid
^{1}H NMR (500 MHz, $CDCl_3$) δ 7.97 – 7.95 (m, 2H), 7.57 – 7.53 (m, 1H), 7.49 – 7.45 (m, 2H), 7.43 – 7.38 (m, 1H), 6.87 (d, *J* = 15.1 Hz, 1H), 6.37 – 6.23 (m, 2H), 1.90 (d, *J* = 6.4 Hz, 3H).
^{13}C NMR (126 MHz, $CDCl_3$) δ 191.0, 145.3, 141.1, 138.3, 132.5, 130.6, 128.5, 128.4, 123.4, 18.9.
HRMS (ESI +ve, *m/z*) calculated for $C_{12}H_{12}ONa$ $[M+Na]^+$ 195.0780; found: 195.0784.

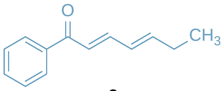

3m

(2*E*,4*E*)-1-phenylhepta-2,4-dien-1-one (**3m**)
Appearance: Yellow solid
^{1}H NMR (500 MHz, $CDCl_3$) δ 7.95 – 7.92 (m, 2H), 7.57 – 7.52 (m, 1H), 7.49 – 7.37 (m, 3H), 6.89 (d, *J* = 14.9 Hz, 1H), 6.35 – 6.23 (m, 2H), 2.28 – 2.21 (m, 2H), 1.08 (t, *J* = 7.4 Hz, 3H).
^{13}C NMR (126 MHz, $CDCl_3$) δ 191.0, 147.9, 145.5, 138.3, 132.5, 128.5, 128.4, 128.2, 123.6, 26.3, 12.9.
HRMS (ESI +ve, *m/z*) Calculated for $C_{13}H_{14}ONa$ $[M+Na]^+$ 209.0937; found: 209.0940.

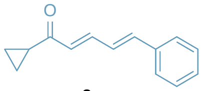

3n

(2*E*,4*E*)-1-cyclopropylhexa-2,4-dien-1-one (**3n**)
Appearance: Yellow solid
^{1}H NMR (500 MHz, $CDCl_3$) δ 7.53 – 7.47 (m, 2H), 7.45 – 7.32 (m, 4H), 7.02 – 6.87 (m, 2H), 6.45 (d, *J* = 15.4 Hz, 1H), 2.20 (tt, *J* = 7.8, 4.5 Hz, 1H), 1.18 – 1.13 (m, 2H), 0.99 – 0.95 (m, 2H).
^{13}C NMR (126 MHz, $CDCl_3$) δ 200.1, 142.0, 141.2, 136.1, 129.8, 129.1, 128.9, 127.2, 126.9, 19.55, 11.2.
HRMS (ESI +ve, *m/z*) calculated for $C_{14}H_{14}ONa$ $[M+Na]^+$ 221.0937; found: 221.0944.

8.2 1,6-conjugate addition products

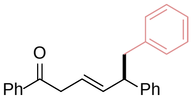

4a

(*E*)-1,5,6-triphenylhex-3-en-1-one (**4a**) (96%, 62.5 mg)
Appearance: Yellow solid
^{1}H NMR (500 MHz, $CDCl_3$) δ 7.88 – 7.86 (m, 2H), 7.56 – 7.52 (m, 1H), 7.46 – 7.41 (m, 2H), 7.29 – 7.26 (m, 5H), 7.24 – 7.12 (m, 5H), 6.28 (dd, *J* = 15.9, 8.1 Hz, 1H), 6.16 (dt, *J* = 15.9, 7.2 Hz, 1H), 3.26 (dt, *J* = 8.1, 6.7 Hz, 1H), 3.08 (d, *J* = 7.2 Hz, 2H), 2.86 (d, *J* = 6.7 Hz 2H).
^{13}C NMR (126 MHz, $CDCl_3$) δ 199.1, 139.6, 137.4, 137.2, 132.9, 132.7, 130.2, 129.4, 128.5, 128.4, 128.3, 128.0, 127.1, 126.2, 126.1, 42.9, 41.3, 40.3.
TLC R_f = 0.55 in hexanes/ethyl acetate 20:1
HRMS (ESI +ve, *m/z*) Calculated for $C_{24}H_{22}ONa$ $[M+Na]^+$ 349.1563; found: 349.1574.

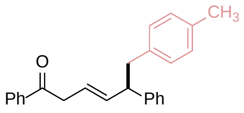

5

(*E*)-1,5-diphenyl-6-(p-tolyl)hex-3-en-1-one (**5**) (76%, 51.6 mg)
Appearance: Yellow solid
^{1}H NMR (500 MHz, $CDCl_3$) δ 7.89 – 7.87 (m, 2H), 7.75 – 7.73 (m, 2H), 7.56 – 7.52 (m, 1H), 7.46 – 7.42 (m, 2H), 7.29 – 7.27 (m, 1H), 7.20 – 7.16 (m, 1H), 7.13 – 7.04 (m, 5H), 6.41 – 6.33 (m, 1H), 6.20 – 6.13 (m, 1H), 3.25 (dt, *J* = 8.0, 7.0 Hz, 1H), 3.08 (d, *J* = 6.7 Hz, 2H), 2.87 – 2.78 (m, 2H), 2.32 (s, 3H).
^{13}C NMR (126 MHz, $CDCl_3$) δ 199.2, 141.6, 137.4, 137.3, 136.4, 135.6, 131.5, 129.3, 129.0, 128.5, 128.5, 128.4, 128.0, 127.0, 126.1, 42.8, 40.9, 40.3, 21.6.
TLC R_f = 0.57 in hexanes/ethyl acetate 20:1
HRMS (ESI +ve, *m/z*) Calculated for $C_{25}H_{24}ONa$ $[M+Na]^+$ 363.1719; found: 363.1729.

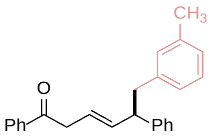

6

(*E*)-1,5-diphenyl-6-(m-tolyl)hex-3-en-1-one (**6**) (68%, 46.2 mg)
Appearance: Yellow solid
^{1}H NMR (500 MHz, $CDCl_3$) δ 7.89 – 7.87 (m, 2H), 7.56 – 7.52 (m, 1H), 7.45 – 7.40 (m, 2H), 7.30 – 7.27 (m, 3H), 7.20 – 7.14 (m, 3H), 7.04 – 7.01 (m, 3H), 6.41 – 6.33 (m, 1H), 6.24 – 6.16 (m, 1H), 3.26 (dt, *J* = 8.1, 6.8 Hz, 1H), 3.08 (d, *J* = 6.8 Hz, 2H), 2.87 – 2.78 (m, 2H), 2.31 (s, 3H).
^{13}C NMR (126 MHz, $CDCl_3$) δ 199.2, 139.5, 138.5, 137.8, 137.4, 137.3, 132.1, 128.8, 128.7, 128.5, 128.4, 128.2, 128.0, 127.0, 126.9, 126.4, 126.1, 42.8, 41.3, 40.3, 21.4.
TLC R_f = 0.57 in hexanes/ethyl acetate 20:1
HRMS (ESI +ve, *m/z*) Calculated for $C_{25}H_{24}ONa$ $[M+Na]^+$ 363.1719; found: 363.1723.

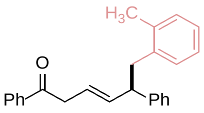

7

(*E*)-1,5-diphenyl-6-(o-tolyl)hex-3-en-1-one (**7**) (56%, 38.0 mg)
Appearance: Yellow solid
^{1}H NMR (500 MHz, $CDCl_3$) δ 7.88 (dd, *J* = 8.3, 1.3 Hz, 2H), 7.57 – 7.53 (m, 1H), 7.45 – 7.43 (m, 2H), 7.26 – 7.23 (m, 4H), 7.17 – 7.09 (m, 5H), 6.36 – 6.28 (m, 1H), 6.20 – 6.09 (m, 1H), 3.25 (dt, *J* = 8.1, 6.8 Hz, 1H), 3.14 (d, *J* = 6.7, 2H), 2.89 – 2.82 (m, 2H), 2.36 (s, 3H).
^{13}C NMR (126 MHz, $CDCl_3$) δ 199.1, 138.5, 137.9, 137.3, 137.2, 136.4, 133.1, 130.9, 128.5, 128.4, 128.2, 128.0, 127.4, 127.1, 126.3, 126.1, 125.7, 43.2, 39.4, 38.7, 19.6.

TLC R_f = 0.57 in hexanes/ethyl acetate 20:1

HRMS (ESI +ve, *m/z*) Calculated for $C_{25}H_{24}ONa$ $[M+Na]^+$ 363.1719; found: 363.1721.

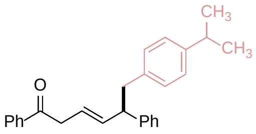

8

(*E*)-6-(3-isopropylphenyl)-1,5-diphenylhex-3-en-1-one (**8**) (50%, 36.8 mg)

appearance: Light yellow solid

^{1}H NMR δ 7.87 – 7.85 (m, 2H), 7.55 – 7.52 (m, 1H), 7.44 – 7.41 (m, 2H), 7.29 – 7.24 (m, 4H), 7.20 – 7.14 (m, 5H), 6.40 – 6.33 (m, 1H), 6.24 – 6.17 (m, 1H), 3.25 (dt, *J* = 8.0, 7.2 Hz, 1H), 3.06 (d, *J* = 7.0 Hz, 2H), 2.91 – 2.85 (m, 2H), 2.81 – 2.76 (m, 1H), 1.23 (d, *J* = 6.9 Hz, 6H).

^{13}C NMR (126 MHz, $CDCl_3$) δ 199.2, 146.7, 137.4, 137.3, 136.8, 132.9, 132.9, 130.0, 129.3, 128.5, 128.4, 128.0, 127.0, 126.3, 126.1, 42.8, 40.9, 40.3, 33.7, 24.0.

HRMS (ESI +ve, *m/z*) calculated for $C_{27}H_{28}ONa$ $[M+Na]^+$ 391.2032; found: 391.2039.

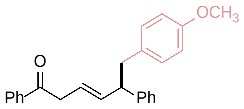

9

(*E*)-6-(4-methoxyphenyl)-1,5-diphenylhex-3-en-1-one (**9**) (62%, 44.2 mg)

Appearance: Light yellow solid

^{1}H NMR (500 MHz, $CDCl_3$) δ 7.88 (dd, *J* = 8.4, 1.3 Hz, 2H), 7.56 – 7.52 (m, 1H), 7.45 – 7.42 (m, 2H), 7.28 – 7.27 (m, 4H), 7.20 – 7.16 (m, 1H), 7.13 – 7.11 (m, 2H), 6.84 – 6.81 (m, 2H), 6.38 – 6.31 (m, 1H), 6.22 – 6.14 (m, 1H), 3.78 (s, 3H), 3.22 (dt, *J* = 8.1, 7.1 Hz, 1H), 3.08 (d, *J* = 7.1 Hz, 2H), 2.84 – 2.77 (m, 2H).

^{13}C NMR (126 MHz, $CDCl_3$) δ 199.2, 158.0, 137.4, 137.3, 132.9, 132.8, 131.6, 130.3, 130.1, 128.5, 128.4, 128.0, 127.0, 126.1, 113.7, 55.2, 42.8, 40.5, 40.5.

TLC R_f = 0.57 in hexane/ethyl acetate 20:1

HRMS (APCI +ve, *m/z*) Calculated for $C_{25}H_{25}O_2$ $[M+H]^+$ 357.1849; found: 357.1846.

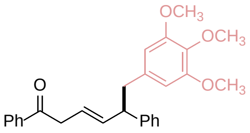

10

(*E*)-1,5-diphenyl-6-(3,4,5-trimethoxyphenyl)hex-3-en-1-one (**10**) (57%, 47.4 mg)

Appearance: Bright yellow solid

^{1}H NMR (500 MHz, $CDCl_3$) δ 7.89 (dd, *J* = 8.4, 1.4 Hz, 2H), 7.57 – 7.53 (m, 1H), 7.47 – 7.41 (m, 2H), 7.37 – 7.26 (m, 4H), 7.21 – 7.16 (m, 1H), 6.40 (s, 2H), 6.38 – 6.34 (m, 1H), 6.21 (dt, *J* = 15.9, 7.1 Hz, 1H), 3.82 (s, 3H), 3.76 (s, 6H), 3.27 (dt, *J* = 7.9, 6.9 Hz, 1H), 3.09 (d, *J* = 7.1 Hz, 2H), 2.81 – 2.79 (m, 2H).

^{13}C NMR (126 MHz, $CDCl_3$) δ 199.1, 152.9, 137.3, 137.2, 136.3, 135.2, 133.0, 132.7, 130.2, 128.6, 128.5, 128.0, 127.2, 126.0, 106.3, 60.8, 56.0, 42.6, 41.5, 40.1.

TLC R_f = 0.57 in hexane/ethyl acetate 20:1

HRMS (APCI +ve, *m/z*) Calculated for $C_{27}H_{29}O_4$ $[M+H]^+$ 417.2060; found: 417.2059.

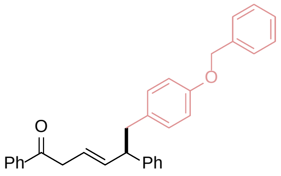

11

(*E*)-6-(4-(benzyloxy)phenyl)-1,5-diphenylhex-3-en-1-one (**11**) (56%, 48.4 mg)
Appearance: Light yellow solid
^{1}H NMR (500 MHz, $CDCl_3$) δ 7.88 (dd, *J* = 8.4, 1.3 Hz, 2H), 7.64 – 7.49 (m, 3H), 7.45 – 7.40 (m, 5H), 7.36 – 7.27 (m, 4H), 7.20 – 7.17 (m, 1H), 7.14 – 7.10 (m, 2H), 6.91 – 6.87 (m, 2H), 6.40 – 6.31 (m, 2H), 6.23 – 6.14 (m, 2H), 5.04 (s, 2H), 3.22 (dt, *J* = 8.1, 7.0 Hz, 1H), 3.08 (d, *J* = 6.8 Hz, 2H), 2.84 – 2.76 (m, 2H).
^{13}C NMR (126 MHz, $CDCl_3$) δ 199.2, 157.3, 144.8, 141.9, 137.4, 137.3, 137.1, 132.9, 132.8, 131.9, 130.4, 130.2, 128.5, 128.4, 128.0, 127.9, 127.5, 127.0, 126.1, 114.7, 70.0, 42.8, 40.5.
TLC R_f = 0.66 in hexane/ethyl acetate 20:1
HRMS (APCI +ve, *m/z*) Calculated for $C_{31}H_{29}O_2$ $[M+H]^+$ 433.2162; found: 433.2156.

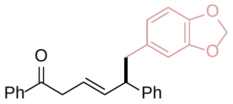

12

(*E*)-6-(benzo[d][1,3]dioxol-5-yl)-1,5-diphenylhex-3-en-1-one (**12**) (62%, 45.9 mg)
^{1}H NMR (500 MHz,) δ 7.89 (dd, *J* = 8.4, 1.3 Hz, 2H), 7.56 – 7.53 (m, 1H), 7.46 – 7.42 (m, 2H), 7.27 (m, 4H), 7.20 – 7.16 (m, 1H), 6.72 – 6.70 (m, 2H), 6.65 (dd, *J* = 7.9, 1.7 Hz, 1H), 6.38 – 6.32 (m, 1H), 6.21 – 6.13 (m, 1H), 5.92 (s, 2H), 3.20 (dt, *J* = 8.1, 7.0 Hz, 1H), 3.07 (d, *J* = 7.4 Hz, 2H), 2.77 (d, *J* = 7.1 Hz, 2H).
^{13}C NMR (126 MHz, $CDCl_3$) δ 199.1, 147.5, 145.9, 137.3, 137.2, 133.4, 132.9, 132.6, 130.2, 128.5, 128.4, 128.0, 127.1, 126.1, 122.3, 109.6, 108.0, 100.8, 42.8, 41.1, 40.5.
HRMS (ESI +ve, *m/z*) calculated for $C_{25}H_{22}O_3Na$ $[M+Na]^+$ 393.1461; found: 393.1461.

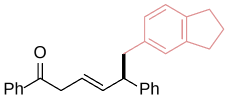

13

(*E*)-6-(2,3-dihydro-1H-inden-5-yl)-1,5-diphenylhex-3-en-1-one (**13**) (66%, 48.3 mg)
Appearance: Light yellow solid
^{1}H NMR (500 MHz, $CDCl_3$) δ 7.88 – 7.86 (m, 2H), 7.55 – 7.52 (m, 1H), 7.44 – 7.41 (m, 2H), 7.31 – 7.27 (m, 4H), 7.20 – 7.08 (m, 4H), 6.40 – 6.33 (m, 1H), 6.26 – 6.17 (m, 1H), 3.25 (dt, *J* = 7.4, 6.9 Hz, 1H), 3.07 (d, *J* = 7.4, 2H), 2.98 – 2.94 (m, 4H), 2.87 – 2.84 (m, 2H), 2.15 – 2.07 (m, 2H).
^{13}C NMR (126 MHz, $CDCl_3$) δ 199.3, 145.0, 144.4, 142.0, 137.5, 137.3, 137.2, 133.1, 132.9, 128.5, 128.4, 128.0, 127.2, 126.1, 125.4, 124.6, 42.8, 41.2, 40.4, 33.0, 32.5, 25.4.
TLC R_f = 0.45 in hexane/ethyl acetate 20:1
HRMS (APCI +ve, *m/z*) Calculated for $C_{27}H_{27}O$ $[M+H]^+$ 367.1900; found: 367.1904.

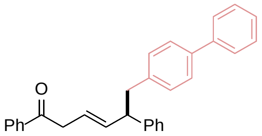

14

(*E*)-6-([1,1'-biphenyl]-4-yl)-1,5-diphenylhex-3-en-1-one (**14**) (88%, 70.7 mg)
Appearance: Light yellow solid

^{1}H NMR (500 MHz, $CDCl_3$) δ 7.90 – 7.88 (m, 2H), 7.59 – 7.51 (m, 5H), 7.45 – 7.40 (m, 5H), 7.34 – 7.28 (m, 6H), 7.20 – 7.17 (m, 1H), 6.40 – 6.34 (m, 1H), 6.21 (dt, *J* = 15.9, 7.1 Hz, 1H), 3.30 (dt, *J* = 7.2, 6.7 Hz, 1H), 3.12 (d, *J* = 7.1 Hz, 2H), 2.91 – 2.89 (d, *J* = 6.7 Hz, 2H).
^{13}C NMR (126 MHz, $CDCl_3$) δ 199.1, 140.9, 139.1, 138.7, 137.3, 137.2, 133.0, 132.6, 130.3, 129.8, 128.7, 128.5, 128.4, 128.0, 127.1, 127.0, 127.0, 127.0, 126.1, 42.9, 41.0, 40.2.
TLC R_f = 0.47 in hexane/ethyl acetate 20:1
HRMS (APCI +ve, *m/z*) Calculated for $C_{30}H_{27}O$ $[M+H]^+$ 403.2056; found: 403.2055.

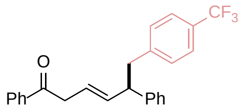

15

(*E*)-1,5-diphenyl-6-(4-(trifluoromethyl)phenyl)hex-3-en-1-one (**15**) (33%, 26.1 mg)
Appearance: Light yellow solid
^{1}H NMR (500 MHz, $CDCl_3$) δ 7.90 (dd, *J* = 8.4, 1.3 Hz, 2H), 7.60 – 7.39 (m, 6H), 7.35 – 7.32 (m, 2H), 7.29 – 7.27 (m, 3H), 7.22 – 7.18 (m, 1H), 6.31 (dd, *J* = 15.9, 6.6 Hz, 1H), 6.20 – 6.12 (m, 1H), 3.29 (dt, *J* = 7.9, 6.6 Hz, 1H), 3.12 (d, *J* = 6.6 Hz, 2H), 2.98 – 2.85 (m, 2H).
^{13}C NMR (126 MHz, $CDCl_3$) δ 198.7, 143.8, 137.2, 137.1, 133.1, 131.9, 130.7, 129.7, 128.8, 128.6 (q, *J* = 20.5 Hz), 128.5, 128.4, 128.0, 127.3, 126.1 (q, *J* = 238.4 Hz), 125.1 (q, *J* = 3.7 Hz), 42.9, 41.0, 40.1.
TLC R_f = 0.70 in hexane/ethyl acetate 20:1
HRMS (APCI +ve, *m/z*) Calculated for $C_{25}H_{22}F_3O$ $[M+H]^+$ 395.1617; found: 395.1634.

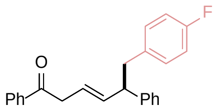

16

(*E*)-6-(4-fluorophenyl)-1,5-diphenylhex-3-en-1-one (**16**) (69%, 47.3 mg)
Appearance: Light yellow solid
^{1}H NMR (500 MHz, $CDCl_3$) δ 7.90 (dd, *J* = 8.3, 1.4 Hz, 2H), 7.86 – 7.82 (m, 1H), 7.58 – 7.53 (m, 1H), 7.48 – 7.43 (m, 2H), 7.31 – 7.27 (m, 2H), 7.21 – 7.15 (m, 4H), 6.96 – 6.94 (m, 2H), 6.33 – 6.29 (m, 1H), 6.21 – 6.12 (m, 1H), 3.23 (dt, *J* = 8.7, 6.8 Hz, 1H), 3.09 (d, *J* = 6.6 Hz, 2H), 2.88 – 2.79 (m, 2H).
^{13}C NMR (126 MHz, $CDCl_3$) δ 198.9, 161.0 (d, *J* = 246.5 Hz), 139.3, 137.2 (d, *J* = 5.0 Hz) , 135.2 (d, *J* = 3.3 Hz),, 133.0, 132.3, 130.5, 128.6, 128.4, 128.3, 128.0, 127.2, 126.1, 115.0 (d, *J* = 20.9 Hz), 42.8, 40.5, 40.4.
TLC R_f = 0.60 in hexane/ethyl acetate 20:1
HRMS (APCI +ve, *m/z*) Calculated for $C_{24}H_{22}FO$ $[M+H]^+$ 345.1649; found: 345.1647.

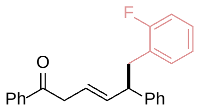

17

(*E*)-6-(2-fluorophenyl)-1,5-diphenylhex-3-en-1-one (**17**) (87%, 59.8 mg)
Appearance: Light yellow solid
^{1}H NMR (500 MHz, $CDCl_3$) δ 7.91 – 7.89 (m, 2H), 7.56 – 7.53 (m, 1H), 7.46 – 7.43 (m, 2H), 7.25 – 7.12 (m, 4H), 7.07 – 6.98 (m, 2H), 6.37 – 6.30 (m, 1H), 6.21 – 6.14 (m, 1H), 3.30 (dt, *J* = 7.4, 6.4 Hz, 1H), 3.14 (d, *J* = 7.1 Hz, 2H), 2.92 (d, *J* = 6.4 Hz, 2H).
^{13}C NMR (126 MHz, $CDCl_3$) δ 198.9, 161.3 (d, *J* = 252.5 Hz), 139.2, 137.3 (d, *J* = 14.6 Hz), 132.9, 132.3, 131.6 (d, *J* = 4.7 Hz), 130.5, 128.5, 128.4, 128.0 (d, *J* = 8.3 Hz), 127.9, 127.1, 126.5, 126.1, 123.9 (d, *J* = 4.3 Hz), 115.3 (d, *J* = 22.5 Hz), 43.1, 39.7, 34.2.
TLC R_f = 0.60 in hexane/ethyl acetate 20:1
HRMS (APCI +ve, *m/z*) Calculated for $C_{24}H_{22}FO$ $[M+H]^+$ 345.1649; found: 345.1647.

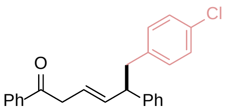

18

(*E*)-6-(4-chlorophenyl)-1,5-diphenylhex-3-en-1-one (**18**) (88%, 63.4 mg)
Appearance: Light yellow solid
^{1}H NMR (500 MHz, $CDCl_3$) δ 7.91 – 7.89 (m, 2H), 7.57 – 7.54 (m, 1H), 7.47 – 7.40 (m, 3H), 7.25 – 7.24 (m, 4H), 7.23 – 7.05 (m, 4H), 6.36 – 6.28 (m, 1H), 6.18 – 6.11 (m, 1H), 3.28 – 3.20 (m, 1H), 3.09 (d, *J* = 6.8 Hz, 2H), 2.88 – 2.77 (m, 2H).
^{13}C NMR (126 MHz, $CDCl_3$) δ 198.9, 138.1, 137.2, 133.1, 132.2, 131.9, 130.6, 129.7, 129.1, 128.6, 128.4, 128.4, 128.0, 127.2, 126.1, 42.8, 40.6, 40.2.
TLC R_f = 0.65 in hexane/ethyl acetate 20:1
HRMS (ESI +ve, *m/z*) Calculated for $C_{25}H_{21}ClONa$ $[M+Na]^+$ 383.1173; found: 383.1165.

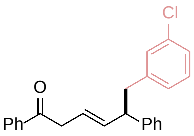

19

(*E*)-6-(3-chlorophenyl)-1,5-diphenylhex-3-en-1-one (**19**) (69%, 49.7 mg)
Appearance: Light yellow solid
^{1}H NMR (500 MHz, $CDCl_3$) δ 7.91 – 7.88 (m, 2H), 7.57 – 7.53 (m, 1H), 7.46 – 7.42 (m, 2H), 7.32 – 7.27 (m, 3H), 7.23 – 7.18 (m, 4H), 7.12 – 7.02 (m, 2H), 6.36 – 6.31 (m, 1H), 6.18 – 6.11 (m, 1H), 3.26 (dt, J = 8.2, 6.9 Hz, 1H), 3.09 (d, *J* = 6.7 Hz, 2H), 2.88 – 2.78 (m, 2H).
^{13}C NMR (126 MHz, $CDCl_3$) δ 198.8, 141.7, 137.2, 137.2, 134.1, 133.1, 132.1, 130.6, 129.5, 129.4, 128.6, 128.4, 128.0, 127.6, 127.2, 126.4, 126.1, 42.9, 40.9, 40.1.
TLC R_f = 0.65 in hexane/ethyl acetate 20:1
HRMS (ESI +ve, *m/z*) Calculated for $C_{25}H_{21}ClONa$ $[M+Na]^+$ 383.1173; found: 383.1179.

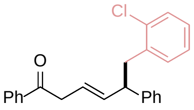

20

(*E*)-6-(2-chlorophenyl)-1,5-diphenylhex-3-en-1-one (**20**) (73%, 52.5 mg)
Appearance: Light yellow solid
^{1}H NMR (500 MHz, $CDCl_3$) δ 7.90 (dd, *J* = 7.7, 2.1 Hz, 2H), 7.56 – 7.53 (m, 1H), 7.46 – 7.43 (m, 2H), 7.34 (dd, *J* = 7.6, 1.7 Hz, 1H), 7.25 – 7.11 (m, 8H), 6.32 – 6.26 (m, 1H), 6.21 – 6.15 (m, 1H), 3.36 (dt, *J* = 7.7, 6.7 Hz, 1H), 3.14 (d, *J* = 6.2 Hz, 2H), 3.05 – 2.96 (m, 2H).
^{13}C NMR (126 MHz, $CDCl_3$) δ 198.9, 137.4, 137.3, 137.2, 134.3, 133.0, 132.2, 131.5, 130.5, 129.5, 128.5, 128.4, 128.1, 127.7, 127.1, 126.6, 126.2, 43.2, 39.5, 38.6.
TLC R_f = 0.65 in hexane/ethyl acetate 20:1
HRMS (ESI +ve, *m/z*) Calculated for $C_{25}H_{21}ClONa$ $[M+Na]^+$ 383.1173; found: 383.1173.

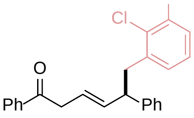

21

(*E*)-6-(2,3-dichlorophenyl)-1,5-diphenylhex-3-en-1-one (**21**) (46%, 36.3 mg)
Appearance: Light yellow solid
^{1}H NMR (500 MHz, $CDCl_3$) δ 7.90 (dd, *J* = 8.4, 1.3 Hz, 2H), 7.57 – 7.54 (m, 1H), 7.46 – 7.43 (m, 2H), 7.32 – 7.30 (m, 1H), 7.26 – 7.24 (m, 4H), 7.20 – 7.07 (m, 3H), 6.32 – 6.26 (m, 1H), 6.20 – 6.13 (m, 1H), 3.36 (dt, *J* = 7.7, 7.1 Hz, 1H), 3.17 – 2.97 (m, 4H).
^{13}C NMR (126 MHz, $CDCl_3$) δ 198.7, 139.9, 137.1, 137.1, 133.2, 133.0, 132.5, 131.8, 130.8, 129.6, 128.6, 128.5, 128.4, 128.1, 127.2, 126.9, 126.2, 43.3, 39.5, 39.3.
TLC R_f = 0.65 in hexane/ethyl acetate 20:1
HRMS (APCI +ve, *m/z*) Calculated for $C_{24}H_{21}Cl_2O$ $[M+H]^+$ 395.0964; found: 395.0967.

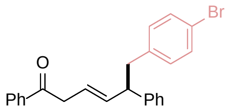

22

(*E*)-6-(4-bromophenyl)-1,5-diphenylhex-3-en-1-one (**22**) (86%, 69.5 mg)
Appearance: Bright yellow solid
^{1}H NMR (500 MHz, $CDCl_3$) δ 7.89 (dd, *J* = 8.3, 1.3 Hz, 2H), 7.58 – 7.54 (m, 1H), 7.47 – 7.43 (m, 2H), 7.40 – 7.37 (m, 2H), 7.28 – 7.26 (m, 3H), 7.25 – 7.18 (m, 2H), 7.10 – 7.07 (m, 2H), 6.35 – 6.29 (m, 1H), 6.18 – 6.11 (m, 1H), 3.24 (dt, *J* = 8.2, 7.0 Hz, 1H), 3.09 (m, *J* = 6.7 Hz, 2H), 2.89 – 2.75 (m, 2H).
^{13}C NMR (126 MHz, $CDCl_3$) δ 198.8, 138.6, 137.2, 133.1, 132.1, 131.3, 131.1, 130.6, 128.6, 128.5, 128.0, 127.2, 126.1, 125.5, 120.0, 42.8, 40.7, 40.1.
TLC R_f = 0.70 in hexane/ethyl acetate 20:1
HRMS (APCI +ve, *m/z*) Calculated for $C_{24}H_{22}BrO$ $[M+H]^+$ 405.0848; found: 405.0848.

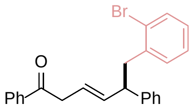

23

(*E*)-6-(2-bromophenyl)-1,5-diphenylhex-3-en-1-one (**23**) (93%, 74.9 mg)
Appearance: Light yellow solid
^{1}H NMR (500 MHz, $CDCl_3$) δ 7.90 (dd, *J* = 8.4, 1.3 Hz, 2H), 7.56 – 7.48 (m, 3H), 7.46 – 7.42 (m, 2H), 7.25 – 7.15 (m, 6H), 7.07 – 7.03 (m, 1H), 6.34 – 6.26 (m, 1H), 6.21 – 6.16 (m, 1H), 3.37 (dt, *J* = 7.3, 6.6 Hz, 1H), 3.15 (d, *J* = 6.3 Hz, 2H), 3.06 – 2.96 (m, 2H).
^{13}C NMR (126 MHz, $CDCl_3$) δ 198.9, 139.2, 137.3, 137.1, 132.9, 132.9, 132.1, 131.6, 130.5, 128.5, 128.4, 128.1, 127.9, 127.2, 127.1, 126.2, 120.3, 43.2, 41.1, 39.6.
TLC R_f = 0.70 in hexane/ethyl acetate 20:1
HRMS (ESI +ve, *m/z*) Calculated for $C_{24}H_{21}BrONa$ $[M+Na]^+$ 427.0668; found: 427.0652.

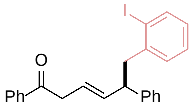

24

(*E*)-6-(2-iodophenyl)-1,5-diphenylhex-3-en-1-one (**24**) (78%, 70.5 mg)
Appearance: Light yellow solid
^{1}H NMR (500 MHz, $CDCl_3$) δ 7.90 (dd, *J* = 8.3, 1.3 Hz, 2H), 7.82 – 7.80 (m, 1H), 7.56 – 7.53 (m, 1H), 7.46 – 7.43 (m, 2H), 7.26 – 7.22 (m, 6H), 7.19 – 7.16 (m, 1H), 6.89 – 6.86 (m, 1H), 6.27 – 6.15 (m, 2H), 3.33 (dt, *J* = 7.8, 6.8 Hz, 1H), 3.16 (d, *J* = 7.4 Hz, 2H), 3.05 – 2.95 (m, 2H).
^{13}C NMR (126 MHz, $CDCl_3$) δ 198.9, 142.4, 139.6, 137.2, 137.1, 133.0, 131.9, 130.7, 130.6, 128.8, 128.6, 128.4, 128.1, 128.1, 127.1, 126.2, 101.4, 45.6, 43.2, 39.9.

TLC R_f = 0.70 in hexane/ethyl acetate 20:1
HRMS (APCI +ve, *m/z*) Calculated for $C_{24}H_{22}IO$ $[M+H]^+$ 453.0710; found: 453.0713.

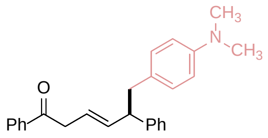

25

(*E*)-6-(4-(dimethylamino)phenyl)-1,5-diphenylhex-3-en-1-one (**25**) (70%, 51.7 mg)
Appearance: Light yellow solid
^{1}H NMR (500 MHz, $CDCl_3$) δ 7.87 (dd, *J* = 8.4, 1.4 Hz, 2H), 7.54 – 7.51 (m, 1H), 7.44 – 7.41 (m, 2H), 7.31 – 7.27 (m, 3H), 7.26– 7.25 (m, 1H), 7.19 – 7.16 (m, 1H), 7.10 – 7.07 (m, 2H), 6.70 – 6.68 (m, 2H), 6.39 – 6.33 (m, 1H), 6.25 – 6.17 (m, 1H), 3.21 (dt, *J* = 8.4, 6.4 Hz, 1H), 3.08 (d, *J* = 7.8 Hz, 2H), 2.91 (s, 6H), 2.84 – 2.71 (m, 2H).
^{13}C NMR (126 MHz, $CDCl_3$) δ 199.4, 148.8, 140.6, 137.5, 137.3, 133.2, 132.8, 130.0, 129.9, 128.5, 128.4, 128.1, 126.9, 126.1, 112.8, 42.8, 40.8, 40.5, 40.4.
TLC R_f = 0.59 in hexane/ethyl acetate 20:1
HRMS (APCI +ve, *m/z*) Calculated for $C_{26}H_{28}NO$ $[M+H]^+$ 370.1093; found: 370.1091.

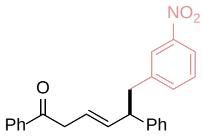

26

(*E*)-6-(3-nitrophenyl)-1,5-diphenylhex-3-en-1-one (**26**) (40%, 29.6 mg)
Appearance: Light yellow solid
^{1}H NMR (500 MHz, $CDCl_3$) δ 8.10 – 8.04 (m, 2H), 7.92 (dd, *J* = 8.4, 1.3 Hz, 2H), 7.59 – 7.54 (m, 2H), 7.48 – 7.42 (m, 3H), 7.25 – 7.23 (m, 4H), 7.21 – 7.16 (m, 1H), 6.35 – 6.25 (m, 1H), 6.17 – 6.09 (m, 1H), 3.33 (dt, *J* = 7.5, 6.2 Hz, 1H), 3.16 (d, *J* = 6.9 Hz, 2H), 3.11 – 2.86 (m, 2H).
^{13}C NMR (126 MHz, $CDCl_3$) δ 198.5, 148.2, 141.8, 137.0, 136.9, 135.6, 133.2, 131.4, 131.3, 129.1, 128.7, 128.5, 128.0, 127.4, 126.1, 124.1, 121.4, 43.1, 40.8, 40.2.
TLC R_f = 0.45 in hexane/ethyl acetate 20:1
HRMS (ESI +ve, *m/z*) Calculated for $C_{24}H_{21}NO_3Na$ $[M+Na]^+$ 394.1414; found: 394.1409.

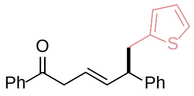

27

(*E*)-1,5-diphenyl-6-(thiophen-2-yl)hex-3-en-1-one (**27**) (31%, 20.6 mg)
Appearance: Light yellow solid
^{1}H NMR (500 MHz, $CDCl_3$) δ 7.90 (dd, *J* = 8.4, 1.4 Hz, 2H), 7.56 – 7.53 (m, 1H), 7.50 – 7.42 (m, 2H), 7.31 – 7.28 (m, 4H), 7.21 – 7.17 (m, 1H), 7.14 – 7.10 (m, 3H), 6.48 – 6.39 (m, 1H), 6.27 – 6.18 (m, 1H), 3.29 (dt, *J* = 8.1, 6.1 Hz, 1H), 3.15 – 3.09 (m, 4H).
^{13}C NMR (126 MHz, $CDCl_3$) δ 198.8, 141.8, 137.2, 133.0, 132.4, 130.8, 130.0, 128.6, 128.4, 128.0, 127.8, 127.2, 126.7, 126.2, 123.8, 42.7, 40.3, 35.2.
TLC R_f = 0.65 in hexane/ethyl acetate 20:1
HRMS (APCI +ve, *m/z*) Calculated for $C_{22}H_{21}OS$ $[M+H]^+$ 333.1307; found: 333.1305.

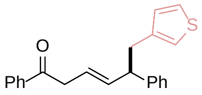

28

(*E*)-1,5-diphenyl-6-(thiophen-3-yl)hex-3-en-1-one (**28**) (27%, 17.9 mg)
Appearance: Light yellow solid
^{1}H NMR (500 MHz, $CDCl_3$) δ 7.89 (dd, *J* = 8.3, 1.3 Hz, 2H), 7.56 – 7.53 (m, 1H), 7.46 – 7.42 (m, 2H), 7.30 – 7.28 (m, 3H), 7.25 – 7.24 (m, 2H), 7.20 – 7.17 (m, 1H), 6.99 – 6.97 (m, 2H), 6.42 – 6.34 (m, 1H), 6.22 – 6.15 (m, 1H), 3.29 (dt, *J* = 7.1, 6.2 Hz, 1H), 3.09 (d, *J* = 7.4 Hz, 2H), 2.90 (d, *J* = 6.9 Hz, 2H).
^{13}C NMR (126 MHz, $CDCl_3$) δ 199.0, 139.8, 137.3, 137.2, 133.0, 132.7, 130.3, 128.8, 128.6, 128.4, 128.0, 127.1, 126.1, 125.3, 121.7, 42.9, 39.6, 35.6.
TLC R_f = 0.65 in hexane/ethyl acetate 20:1
HRMS (APCI +ve, *m/z*) Calculated for $C_{22}H_{21}OS$ $[M+H]^+$ 333.1307; found: 333.1303.

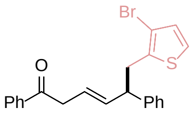

29

(*E*)-6-(3-bromothiophen-2-yl)-1,5-diphenylhex-3-en-1-one (**29**) (33%, 27.0 mg)
Appearance: Light yellow solid
^{1}H NMR (500 MHz, $CDCl_3$) δ 7.93 – 7.91 (m, 2H), 7.57 – 7.54 (m, 1H), 7.47 – 7.43 (m, 2H), 7.31 – 7.26 (m, 3H), 7.21 – 7.17 (m, 1H), 7.15 – 7.12 (m, 1H), 7.11 – 7.08 (m, 1H), 6.90 – 6.89 (m, 1H), 6.45 – 6.37 (m, 1H), 6.22 – 6.16 (m, 1H), 3.36 (dt, *J* = 7.4, 6.8 Hz, 1H), 3.18 (d, *J* = 7.4 Hz, 2H), 3.11 – 3.05 (m, 2H).
^{13}C NMR (126 MHz, $CDCl_3$) δ 198.5, 137.2, 137.1, 136.6, 133.0, 131.6, 131.1, 129.8, 128.6, 128.4, 128.1, 127.2, 126.2, 124.0, 110.1, 42.9, 40.0, 34.2.
TLC R_f = 0.68 in hexane/ethyl acetate 20:1
HRMS (APCI +ve, *m/z*) Calculated for $C_{22}H_{20}BrOS$ $[M+H]^+$ 411.1304; found: 411.1300.

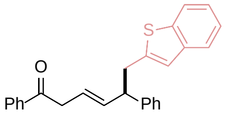

30

(*E*)-6-(benzo[b]thiophen-2-yl)-1,5-diphenylhex-3-en-1-one (**30**) (67%, 51.2 mg)
Appearance: Light yellow solid
^{1}H NMR (500 MHz, $CDCl_3$) δ 7.93 – 7.91 (m, 2H), 7.78 – 7.76 (m, 1H), 7.67 – 7.65 (m, 1H), 7.57 – 7.53 (m, 1H), 7.46 – 7.40 (m, 2H), 7.33 – 7.28 (m, 7H), 7.22 – 7.19 (m, 1H), 6.51 – 6.46 (m, 1H), 6.31 – 6.22 (m, 1H), 3.42 (dt, *J* = 8.0, 6.8 Hz, 1H), 3.20 – 3.13 (m, 4H).
^{13}C NMR (126 MHz, $CDCl_3$) δ 198.7, 143.1, 140.0, 139.7, 137.2, 137.1, 133.0, 132.0, 130.9, 128.6, 128.5, 128.1, 127.3, 126.2, 124.1, 123.6, 122.9, 122.4, 122.1, 42.7, 39.8, 36.1.
TLC R_f = 0.66 in hexane/ethyl acetate 20:1
HRMS (APCI +ve, *m/z*) Calculated for $C_{26}H_{23}OS$ $[M+H]^+$ 383.1307; found: 383.1301.

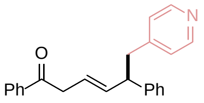

31

(*E*)-1,5-diphenyl-6-(pyridin-4-yl)hex-3-en-1-one (**31**) (86%, 56.2 mg)
Appearance: Light yellow solid
^{1}H NMR (500 MHz, $CDCl_3$) δ 8.51 – 8.50 (m, 2H), 7.93 – 7.91 (m, 2H), 7.60 – 7.56 (m, 1H), 7.50 – 7.45 (m, 2H), 7.41 – 7.27 (m, 2H), 7.25 – 7.18 (m, 5H), 6.35 – 6.28 (m, 1H), 6.16 – 6.08 (m, 1H), 3.32 – 3.28 (m, 1H), 3.16 (d, *J* = 7.5 Hz, 2H), 3.00 – 2.78 (m, 2H).
^{13}C NMR (126 MHz, $CDCl_3$) δ 198.4, 150.0 148.2, 137.0, 136.7, 133.3, 131.3, 131.1, 128.7, 128.5, 128.0, 127.5, 126.1, 125.2, 43.0, 40.6, 39.5.

TLC R$_f$ **=** 0.59 in hexane/ethyl acetate 20:1
HRMS (APCI +ve, *m/z*) Calculated for $C_{23}H_{22}NO$ [M+H]$^+$ 328.1823; found: 328.1803.

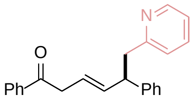

32

(*E*)-1,5-diphenyl-6-(pyridin-2-yl)hex-3-en-1-one (**32**) (76%, 49.7 mg)
Appearance: Light yellow solid
^{1}H NMR (500 MHz, $CDCl_3$) δ 8.54 – 8.52 (m, 1H), 7.92 – 7.90 (m, 2H), 7.61 – 7.58 (m, 1H), 7.55 – 7.52 (m, 1H), 7.45 – 7.42 (m, 2H), 7.25 – 7.21 (m, 5H), 7.18 – 7.15 (m, 1H), 7.13 – 7.10 (m, 1H), 6.36 – 6.31 (m, 1H), 6.25 – 6.18 (m, 1H), 3.49 (dt, *J* = 8.6, 7.0 Hz, 1H), 3.24 – 3.07 (m, 4H).
^{13}C NMR (126 MHz, $CDCl_3$) δ 198.9, 159.7, 149.0, 137.3, 137.2, 136.5, 132.9, 132.3, 130.5, 128.5, 128.4, 128.1, 127.1, 126.1, 123.9, 121.4, 43.3, 43.3, 39.4.
TLC R$_f$ **=** 0.59 in hexane/ethyl acetate 20:1
HRMS (APCI +ve, *m/z*) Calculated for $C_{23}H_{22}NO$ [M+H]$^+$ 328.1823; found: 328.1803.

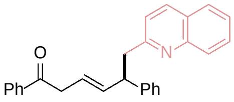

33

(*E*)-1,5-diphenyl-6-(quinolin-2-yl)hex-3-en-1-one (**33**) (45%, 33.9 mg)
Appearance: Light yellow solid
^{1}H NMR (500 MHz, $CDCl_3$) δ 8.08 – 7.98 (m, 2H), 7.92 – 7.90 (m, 2H), 7.78 – 7.76 (m, 1H), 7.69 – 7.66 (m, 1H), 7.54 – 7.36 (m, 6H), 7.24 – 7.22 (m, 3H), 7.19 – 7.14 (m, 1H), 6.44 – 6.36 (m, 1H), 6.30 – 6.25 (m, 1H), 3.65 (dt, *J* = 8.9, 7.1 Hz, 1H), 3.31 – 3.13 (m, 4H).
^{13}C NMR (126 MHz, $CDCl_3$) δ 199.0, 160.4, 147.8, 137.2, 137.2, 136.3, 132.9, 132.4, 130.4, 129.4, 128.8, 128.5, 128.4, 128.1, 127.5, 127.1, 126.8, 126.2, 125.9, 122.1, 44.2, 43.3, 39.1.
TLC R$_f$ **=** 0.59 in hexane/ethyl acetate 20:1
HRMS (ESI +ve, *m/z*) Calculated for $C_{27}H_{23}NONa$ [M+Na]$^+$ 400.1672; found: 400.1675**.**

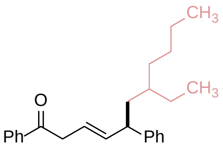

34

(*E*)-7-ethyl-1,5-diphenylundec-3-en-1-one (**34**) (27%, 18.8 mg)
Appearance: Light yellow solid
^{1}H NMR (500 MHz, $CDCl_3$) δ 7.98 (dd, *J* = 8.3, 1.4 Hz, 2H), 7.70 – 7.65 (m, 3H), 7.48 – 7.43 (m, 4H), 7.32 – 7.28 (s, 1H), 6.19 – 6.14 (m, 1H), 6.05 – 6.02 (dt, *J* = 16.0, 6.0 Hz, 1H), 3.14 (d, *J* = 7.8 Hz, 2H), 2.81 – 2.76 (m, 1H), 1.29 – 1.25 (m, 11H), 0.89 – 0.86 (m, 6H).
^{13}C NMR (126 MHz, $CDCl_3$) δ 200.1, 139.7, 136.6, 133.6, 132.1, 128.7, 128.5, 128.4, 128.1, 127.7, 126.5, 45.2, 44.4, 39.2, 36.0, 33.1, 31.7, 29.3, 22.4, 14.0, 10.9.
TLC R$_f$ **=** 0.30 in hexane/ethyl acetate 20:1
HRMS (APCI +ve, *m/z*) Calculated for $C_{25}H_{33}O$ [M+H]$^+$ 349.2212; found: 349.2218.

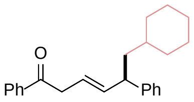

35

(*E*)-6-cyclohexyl-1,5-diphenylhex-3-en-1-one (**35**) (34%, 22.6 mg)

Appearance: Light yellow solid

^{1}H NMR (500 MHz, $CDCl_3$) δ 7.99 – 7.97 (m, 2H), 7.76 – 7.64 (m, 3H), 7.49 – 7.43 (m, 4H), 7.34 – 7.30 (m, 1H), 6.25 – 6.17 (m, 1H), 5.96 – 5.91 (m, 1H), 3.20 (d, J = 7.5 Hz, 2H), 2.73 – 2.62 (m, 1H), 1.51 – 1.45 (m, 2H), 1.31 – 1.16 (m, 11H).

^{13}C NMR (126 MHz, $CDCl_3$) δ 200.1, 140.3, 136.6, 133.7, 132.1, 128.7, 128.6, 128.4, 128.1, 127.7, 126.5, 45.2, 42.7, 38.1, 33.4, 25.7, 25.3, 25.0.

TLC R_f = 0.32 in hexane/ethyl acetate 20:1

HRMS (APCI +ve, *m/z*) Calculated for $C_{24}H_{29}O$ $[M+H]^+$ 333.2056; found: 333.2060.

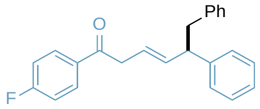

36

(*E*)-1-(4-fluorophenyl)-5,6-diphenylhex-3-en-1-one (**36**) (49%, 33.7 mg)

Appearance: Light yellow solid

^{1}H NMR (500 MHz, $CDCl_3$) δ 7.90 – 7.87 (m, 2H), 7.52 – 7.46 (m, 2H), 7.30 – 7.27 (m, 4H), 7.21 – 7.17 (m, 4H), 7.12 – 7.07 (m, 2H), 6.37 – 6.31 (m, 1H), 6.19 – 6.13 (m, 1H), 3.26 (d, *J* = 7.7, 6.3 Hz, 1H), 3.05 (d, *J* = 7.4 Hz, 2H), 2.90 – 2.81 (m, 2H).

^{13}C NMR (126 MHz, $CDCl_3$) δ 197.4, 165.7 (d, *J* = 250.9 Hz), 139.5, 137.3, 134.1, 132.5, 130.7 (d, *J* = 9.3 Hz), 130.3, 128.8, 128.5, 128.4, 128.3, 126.3, 126.2, 115.5 (d, *J* = 22.5 Hz), 42.7, 41.3, 40.3.

TLC R_f = 0.60 in hexane/ethyl acetate 20:1

HRMS (APCI +ve, *m/z*) Calculated for $C_{24}H_{22}FO$ $[M+H]^+$ 345.1649; found: 345.1647.

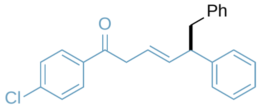

37

(*E*)-1-(4-chlorophenyl)-5,6-diphenylhex-3-en-1-one (**37**) (46%, 33.1 mg)

Appearance: Light yellow solid

^{1}H NMR (500 MHz, $CDCl_3$) δ 7.81 – 7.79 (m, 2H), 7.48 – 7.44 (m, 2H), 7.41 – 7.39 (m, 2H), 7.30 – 7.27 (m, 4H), 7.22 – 7.17 (m, 4H), 6.37 – 6.31 (m, 1H), 6.21 – 6.13 (m, 1H), 3.25 (dt, *J* = 7.3, 6.7 Hz, 1H), 3.03 (d, *J* = 7.5 Hz, 2H), 2.90 – 2.80 (m, 2H).

^{13}C NMR (126 MHz, $CDCl_3$) δ 197.8, 139.4, 137.2, 135.5, 134.1, 132.4, 129.5, 129.4, 128.8, 128.5, 128.4, 128.3, 127.2, 126.2, 126.1, 42.8, 41.3, 40.4.

TLC R_f = 0.65 in hexane/ethyl acetate 20:1

HRMS (ESI +ve, *m/z*) Calculated for $C_{24}H_{21}ClONa$ $[M+Na]^+$ 383.1173; found: 383.1179.

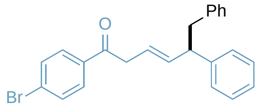

38

(*E*)-1-(4-bromophenyl)-5,6-diphenylhex-3-en-1-one (**38**) (46%, 37.2 mg)

Appearance: Light yellow solid

^{1}H NMR (500 MHz, $CDCl_3$) δ 7.89 (dd, *J* = 7.4, 2.0 Hz, 2H), 7.76 – 7.73 (m, 2H), 7.60 – 7.58 (m, 2H), 7.52 – 7.47 (m, 2H), 7.32 – 7.29 (m, 2H), 7.24 – 7.19 (m, 4H), 6.39 – 6.33 (m, 1H), 6.23 – 6.15 (m, 1H), 3.26 (dt, *J* = 8.2, 6.5 Hz, 1H), 3.05 (d, *J* = 7.8 Hz, 2H), 2.92 – 2.83 (m, 2H).

^{13}C NMR (126 MHz, $CDCl_3$) δ 198.0, 139.4, 135.9, 132.4, 131.8, 130.4, 129.6, 129.4, 128.8, 128.5, 128.4, 128.3, 127.2, 126.3, 126.1, 42.8, 41.3, 40.4.

TLC R_f = 0.70 in hexane/ethyl acetate 20:1

HRMS (APCI +ve, *m/z*) Calculated for $C_{24}H_{22}BrO$ $[M+H]^+$ 405.0848; found: 405.0848.

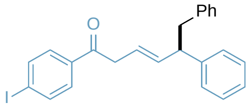

39

(*E*)-1-(4-iodophenyl)-5,6-diphenylhex-3-en-1-one (**39**) (46%, 41.6 mg)
Appearance: Light yellow solid
^{1}H NMR (500 MHz, $CDCl_3$) δ 7.82 – 7.78 (m, 2H), 7.57 – 7.55 (m, 2H), 7.48 – 7.44 (m, 6H), 7.30 – 7.27 (m, 2H), 7.21 – 7.18 (m, 2H), 6.36 – 6.29 (m, 1H), 6.18 – 6.12 (m, 1H), 3.23 (dt, *J* = 8.1, 7.1 Hz, 1H), 3.03 (d, *J* = 7.6 Hz, 2H), 2.89 – 2.80 (m, 2H).
^{13}C NMR (126 MHz, $CDCl_3$) δ 198.4, 139.4, 137.8, 136.4, 134.1, 132.4, 131.2, 130.4, 128.8, 128.5, 128.4, 128.3, 127.2, 126.1, 100.9, 42.7, 41.3, 40.4.
TLC R_f = 0.70 in hexane/ethyl acetate 20:1
HRMS (ESI +ve, *m/z*) Calculated for $C_{24}H_{21}IONa$ [M+Na]$^+$ 475.0529; found: 475.0525.

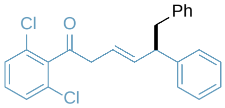

40

(*E*)-1-(2,6-dichlorophenyl)-5,6-diphenylhex-3-en-1-one (**40**) (71%, 56.0 mg)
Appearance: Light yellow solid
^{1}H NMR (500 MHz, $CDCl_3$) δ 7.31 – 7.28 (m, 1H), 7.27 – 7.20 (m, 12H), 6.34 (dd, *J* = 15.9, 8.0 Hz, 1H), 6.15 (dt, *J* = 15.9, 7.1 Hz, 1H), 3.33 – 3.25 (m, 1H), 3.01 (d, *J* = 7.0 Hz, 2H), 2.98 – 2.80 (m, 2H).
^{13}C NMR (126 MHz, $CDCl_3$) δ 200.7, 139.6, 139.5, 137.4, 132.2, 130.5, 130.5, 130.4, 129.4, 128.4, 128.3, 128.1, 127.1, 126.2, 126.1, 48.0, 41.1, 38.9.
TLC R_f = 0.65 in hexane/ethyl acetate 20:1
HRMS (APCI +ve, *m/z*) Calculated for $C_{24}H_{21}Cl_2O$ [M+H]$^+$ 395.0964; found: 395.0967.

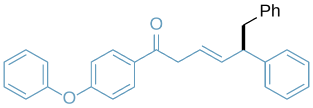

41

(*E*)-1-(4-phenoxyphenyl)-5,6-diphenylhex-3-en-1-one (**41**) (48%, 40.1 mg)
Appearance: Light yellow solid
^{1}H NMR (500 MHz, $CDCl_3$) δ 7.88 – 7.85 (m, 2H), 7.41 – 7.37 (m, 2H), 7.29 – 7.27 (m, 5H), 7.22 – 7.16 (m, 6H), 7.07 – 7.04 (m, 2H), 6.98 – 6.95 (m, 2H), 6.37 – 6.31 (m, 1H), 6.17 (dt, *J* = 15.9, 6.9 Hz, 1H), 3.26 (dt, *J* = 8.3, 7.2 Hz, 1H), 3.03 (d, *J* = 6.9 Hz, 2H), 2.86 (d, *J* = 7.2 Hz, 2H).
^{13}C NMR (126 MHz, $CDCl_3$) δ 197.6, 161.8, 155.5, 139.6, 137.4, 132.7, 131.9, 130.3, 130.2, 130.0, 129.4, 128.4, 128.3, 127.1, 126.2, 126.1, 124.5, 120.1, 117.3, 42.6, 41.4, 40.4.
TLC R_f = 0.66 in hexane/ethyl acetate 20:1
HRMS (APCI +ve, *m/z*) Calculated for $C_{30}H_{27}O_2$ [M+H]$^+$ 419.2005; found: 419.2003.

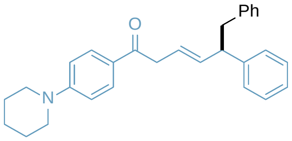

42

(*E*)-5,6-diphenyl-1-(4-(piperidin-1-yl)phenyl)hex-3-en-1-one (**42**) (52%, 42.5 mg)
Appearance: Light yellow solid

^{1}H NMR (500 MHz, $CDCl_3$) δ 7.81 – 7.79 (m, 2H), 7.28 – 7.27 (m, 1H), 7.25 – 7.13 (m, 9H), 6.84 – 6.81 (m, 2H), 6.34 – 6.29 (m, 1H), 6.20 – 6.15 (m, 1H), 3.37 – 3.33 (m, 4H), 3.25 (dt, J = 7.3, 6.2 Hz, 1H), 2.98 (d, J = 7.5 Hz, 2H), 2.88 – 2.80 (m, 2H), 1.67 – 1.64 (m, 6H).
^{13}C NMR (126 MHz, $CDCl_3$) δ 197.1, 154.3, 139.8, 137.6, 133.2, 130.2, 129.8, 129.5, 128.3, 128.2, 126.9, 126.6, 126.1, 126.0, 113.2, 48.6, 42.2, 41.4, 40.6, 25.3, 24.3.
TLC R_f = 0.60 in hexane/ethyl acetate 20:1
HRMS (APCI +ve, *m/z*) Calculated for $C_{29}H_{32}NO$ [M+H]$^+$ 410.2478; found: 410.2477.

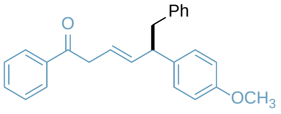

43

(*E*)-5-(4-methoxyphenyl)-1,6-diphenylhex-3-en-1-one (**43**) (78%, 55.5 mg)
Appearance: Light yellow solid
^{1}H NMR (500 MHz, $CDCl_3$) δ 7.87 (dd, J = 8.4, 1.4 Hz, 2H), 7.55 – 7.49 (m, 2H), 7.45 – 7.40 (m, 2H), 7.30 – 7.27 (m, 2H), 7.22 – 7.19 (m, 4H), 6.83 – 6.79 (m, 2H), 6.26 (dd, J = 15.9, 8.0 Hz, 1H), 6.02 (dt, J = 15.9, 7.1 Hz, 1H), 3.78 (s, 3H), 3.24 (dt, J = 8.0, 7.0 Hz, 1H), 3.06 (d, J = 7.1 Hz, 2H), 2.85 – 2.83 (m, 2H).
^{13}C NMR (126 MHz, $CDCl_3$) δ 199.2, 158.8, 139.7, 137.3, 132.9, 130.5, 130.2, 129.5, 129.4, 128.5, 128.3, 128.0, 127.2, 126.1, 113.8, 55.2, 43.0, 41.5, 40.3.
TLC R_f = 0.60 in hexane/ethyl acetate 20:1
HRMS (APCI +ve, *m/z*) Calculated for $C_{25}H_{25}O_2$ [M+H]$^+$ 357.1852; found: 357.1849.

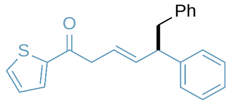

44

(*E*)-5,6-diphenyl-1-(thiophen-2-yl)hex-3-en-1-one (**44**) (42%, 27.9 mg)
Appearance: Light yellow solid
^{1}H NMR (500 MHz, $CDCl_3$) δ 7.63 – 7.60 (m, 2H), 7.29 – 7.27 (m, 6H), 7.22 – 7.17 (m, 4H), 7.11 – 7.09 (m, 1H), 6.35 (dd, J = 15.9, 8.0 Hz, 1H), 6.16 (dt, J = 15.9, 7.1 Hz, 1H), 3.30 – 3.22 (m, 1H), 3.01 (d, J = 7.1 Hz, 2H), 2.86 (d, J = 7.2 Hz, 2H).
^{13}C NMR (126 MHz, $CDCl_3$) δ 191.9, 144.7, 139.4, 137.3, 133.6, 132.4, 131.8, 130.4, 129.4, 128.4, 128.3, 128.0, 127.1, 126.2, 126.1, 43.7, 41.3, 40.7.
TLC R_f = 0.60 in hexane/ethyl acetate 20:1
HRMS (APCI +ve, *m/z*) Calculated for $C_{22}H_{21}OS$ [M+H]$^+$ 333.1307; found: 333.13059.

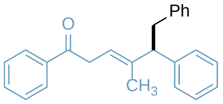

45

(*E*)-4-methyl-1,5,6-triphenylhex-3-en-1-one (**45**) (74%, 50.3 mg)
Appearance: Light yellow solid
^{1}H NMR (500 MHz, $CDCl_3$) δ 7.89 (dd, J = 8.4, 1.3 Hz, 2H), 7.56 – 7.53 (m, 1H), 7.47 – 7.43 (m, 2H), 7.30 – 7.27 (m, 2H), 7.26 – 7.13 (m, 6H), 7.06 – 7.03 (m, 2H), 6.12 (t, J = 6.8 Hz, 1H), 3.22 – 3.08 (m, 3H), 2.88 (d, J = 6.9 Hz, 2H), 1.86 (s, 3H).
^{13}C NMR (126 MHz, $CDCl_3$) δ 199.4, 140.0, 139.0, 138.0, 137.3, 132.9, 129.2, 128.8, 128.5, 128.2, 128.0, 127.9, 126.9, 126.1, 126.0, 47.0, 42.1, 40.1, 15.3.
TLC R_f = 0.60 in hexane/ethyl acetate 20:1
HRMS (ESI +ve, *m/z*) Calculated for $C_{25}H_{24}ONa$ [M+Na]$^+$ 363.1719; found: 363.1709.

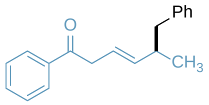

46

(*E*)-5-methyl-1,6-diphenylhex-3-en-1-one (**46**) (63%, 33.1 mg)
Appearance: Light yellow solid
^{1}H NMR (500 MHz, $CDCl_3$) 7.89 – 7.87 (m, 2H), 7.59 – 7.55 (m, 1H), 7.48 – 7.45 (m, 2H), 7.33 – 7.31 (m, 2H), 7.24 – 7.21 (m, 3H), 5.65 – 5.56 (m, 2H), 3.28 (d, *J* = 7.8 Hz, 2H), 3.10 – 3.03 (m, 1H), 2.78 – 2.75 (m, 2H), 1.60 (d, *J* = 5.0 Hz, 3H).
^{13}C NMR (126 MHz, $CDCl_3$) δ 199.6, 140.0, 137.4, 133.5, 132.8, 129.4, 128.5, 128.2, 128.1, 126.0, 125.5, 43.1, 41.6, 40.1, 17.9.
TLC R_f = 0.50 in hexane/ethyl acetate 20:1
HRMS (ESI +ve, *m/z*) Calculated for $C_{19}H_{20}ONa$ [M+Na]$^+$ 287.1406; found: 287.1407.

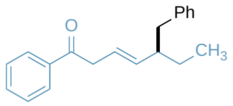

47

(*E*)-5-benzyl-1-phenylhept-3-en-1-one (**47**) (58%, 32.2 mg)
Appearance: Light yellow solid
^{1}H NMR (500 MHz, $CDCl_3$) δ 7.86 – 7.84 (m, 2H), 7.55 – 7.51 (m, 1H), 7.44 – 7.41 (m, 2H), 7.28 – 7.27 (m, 2H), 7.20 – 7.16 (m, 3H), 5.69 – 5.68 (m, 2H), 3.24 (d, *J* = 7.2 Hz, 2H), 3.04 – 2.97 (m, 1H), 2.73 (d, *J* = 7.0 Hz, 2H), 1.94 – 1.88 (m, 2H), 0.85 (t, *J* = 7.3 Hz, 3H).
^{13}C NMR (126 MHz, $CDCl_3$) δ 199.7, 140.0, 137.5, 132.8, 132.8, 131.2, 129.5, 128.5, 128.2, 128.1, 126.0, 43.2, 41.7, 40.2, 25.5, 13.7.
TLC R_f = 0.50 in hexane/ethyl acetate 20:1
HRMS (ESI +ve, *m/z*) Calculated for $C_{20}H_{22}ONa$ [M+Na]$^+$ 301.1562; found: 301.1566.

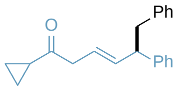

48

(*E*)-1-cyclopropyl-5-methyl-6-phenylhex-3-en-1-one (**48**) (44%, 25.1 mg)
Appearance: Light yellow solid
^{1}H NMR (500 MHz, $CDCl_3$) δ 7.34 – 7.27 (m, 6H), 7.21 – 7.18 (m, 4H), 6.13 (dd, *J* = 15.9, 8.0 Hz, 1H), 5.93 (dt, *J* = 15.9, 7.2 Hz, 1H), 3.12 (dt, *J* = 7.2, 6.6 Hz, 1H), 2.78 (d, *J* = 7.3 Hz, 2H), 2.68 – 2.66 (m, 2H), 1.92 – 1.88 (m, 1H), 1.00 – 0.97 (m, 2H), 0.84 – 0.81 (m, 2H).
^{13}C NMR (126 MHz, $CDCl_3$) δ 209.7, 139.6, 137.4, 132.7, 130.2, 129.4, 128.5, 128.3, 127.1, 126.1, 126.1, 48.1, 41.4, 40.3, 21.0, 10.8.
TLC R_f = 0.50 in hexane/ethyl acetate 20:1
HRMS (APCI +ve, *m/z*) Calculated for $C_{21}H_{23}O$ [M+H]$^+$ 291.1743; found: 291.1740.

9. ^{1}H and ^{13}C NMR Spectra

9.1 1,6-conjugate addition electrophilic substrates

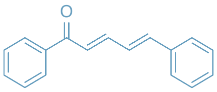

3a

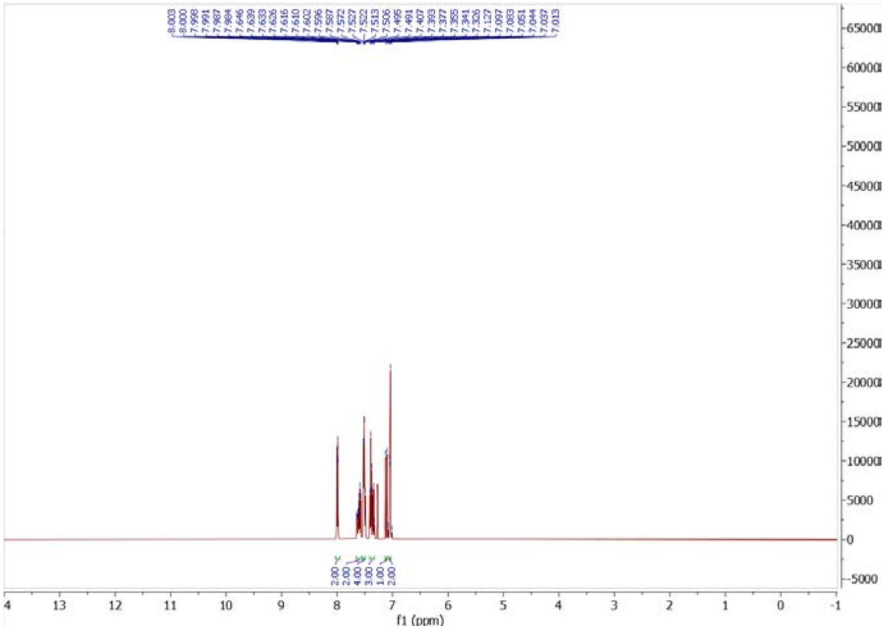

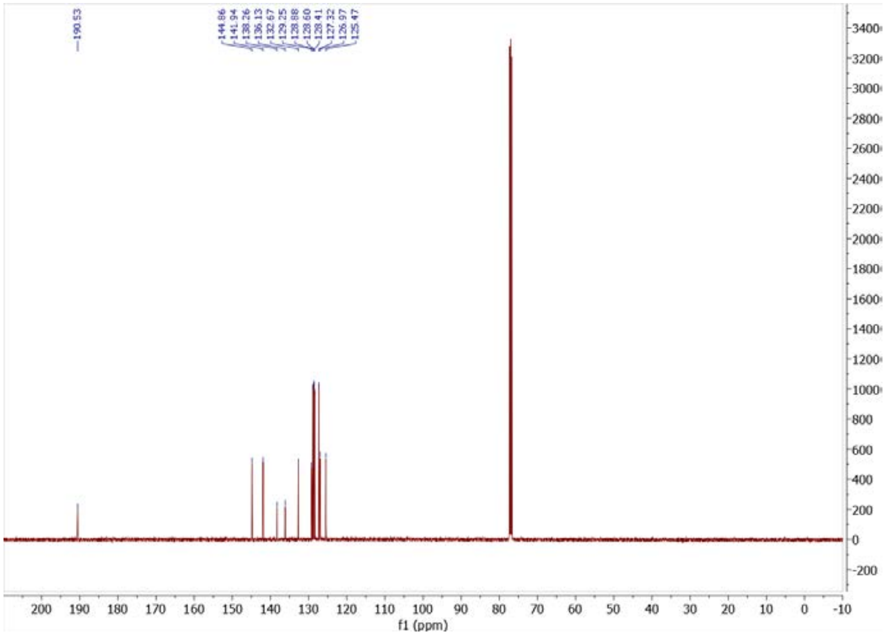

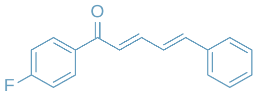

3b

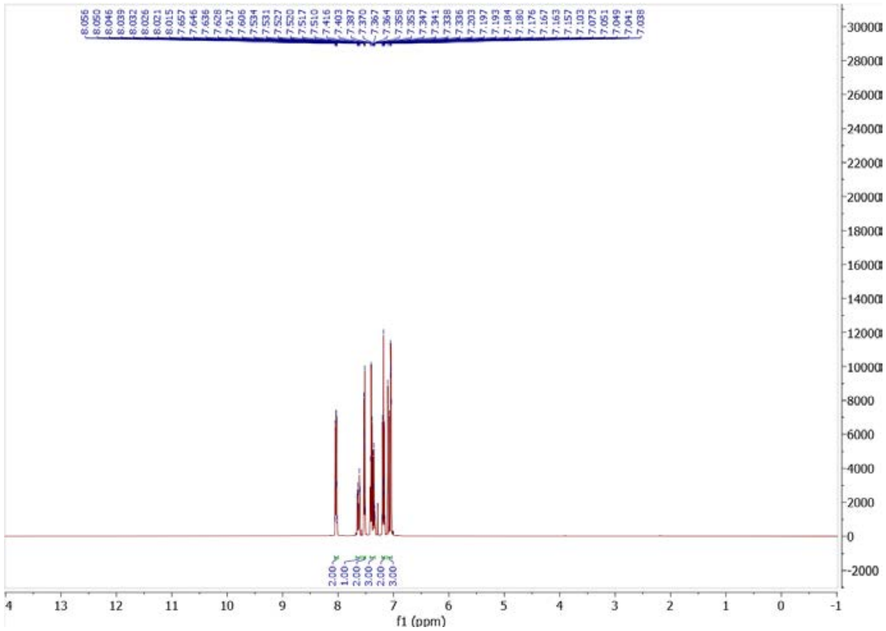

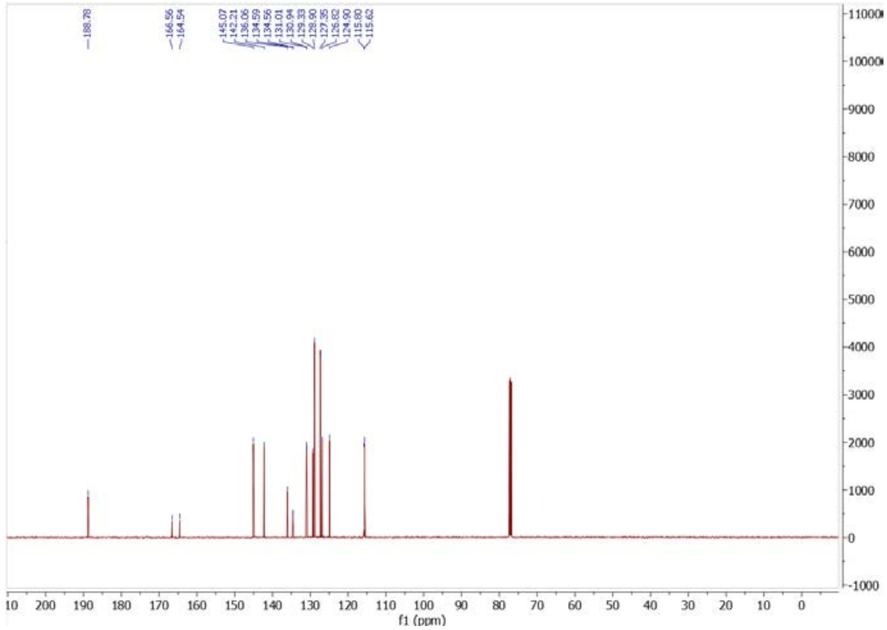

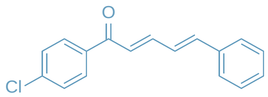
O
Cl

3c

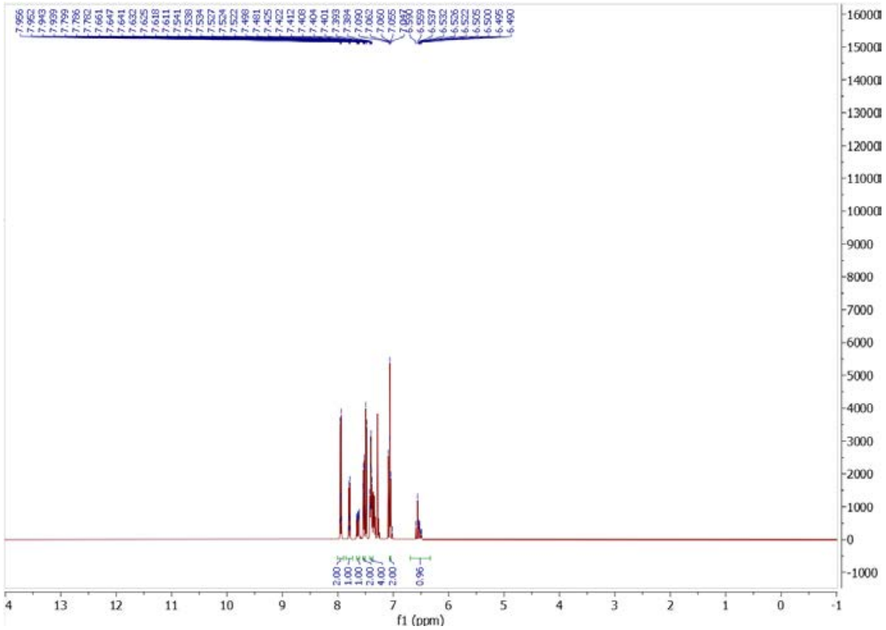
7.966
7.962
7.943
7.939
7.799
7.786
7.782
7.661
7.647
7.641
7.632
7.625
7.618
7.611
7.541
7.538
7.534
7.527
7.524
7.522
7.498
7.481
7.425
7.422
7.412
7.408
7.404
7.401
7.393
7.384
7.090
7.062
7.060
7.055
6.559
6.537
6.532
6.526
6.522
6.505
6.500
6.495
6.490
2.00
1.00
1.00
2.00
4.00
2.00
0.96
f1 (ppm)

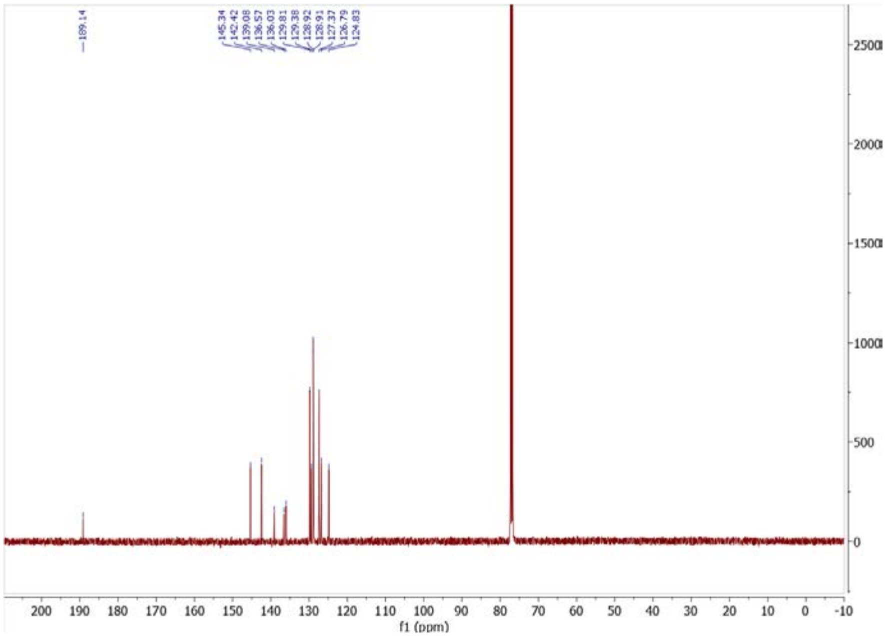
189.14
145.34
142.42
139.08
136.57
136.03
129.81
129.38
128.92
128.91
127.37
126.79
124.83
f1 (ppm)

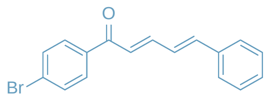
O
Br

3d

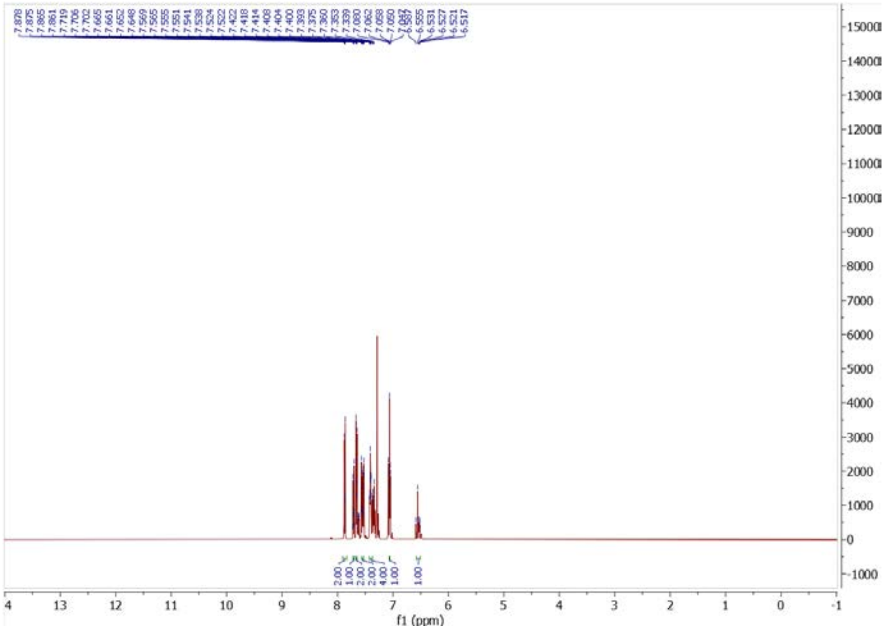
f1 (ppm)

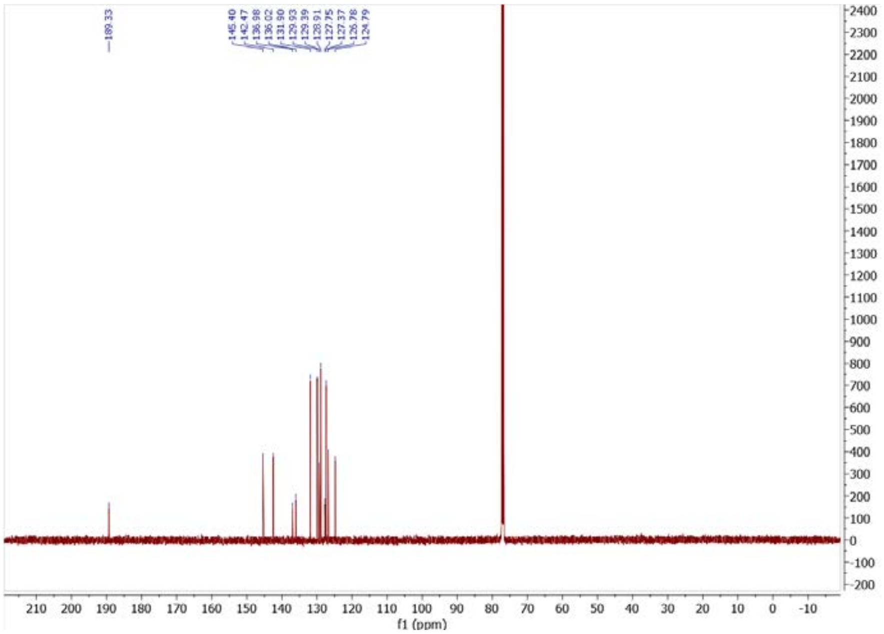
189.33
145.40
142.47
136.98
136.02
131.90
129.93
129.39
128.91
127.75
127.37
126.78
124.79
f1 (ppm)

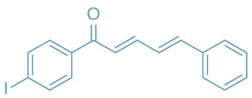
O
I

3e

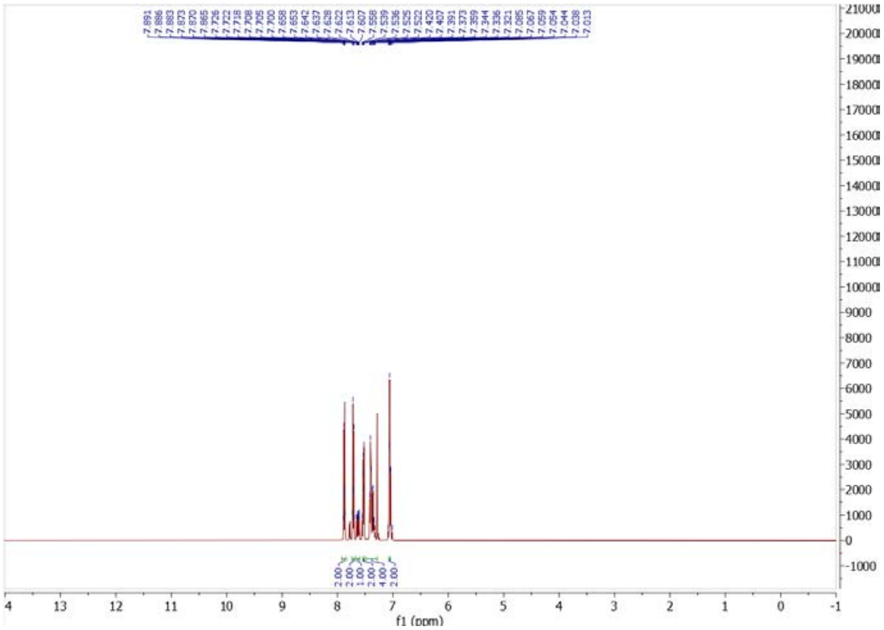
f1 (ppm)

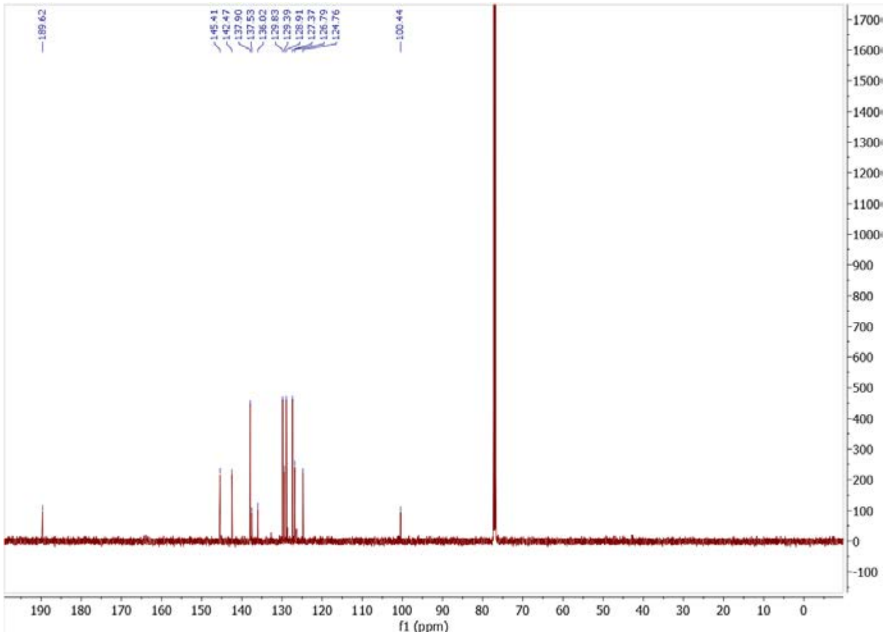
189.62
145.41
142.47
137.90
137.53
136.02
129.83
129.39
128.91
127.37
126.79
124.76
100.44
f1 (ppm)

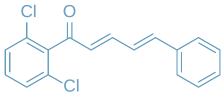

3f

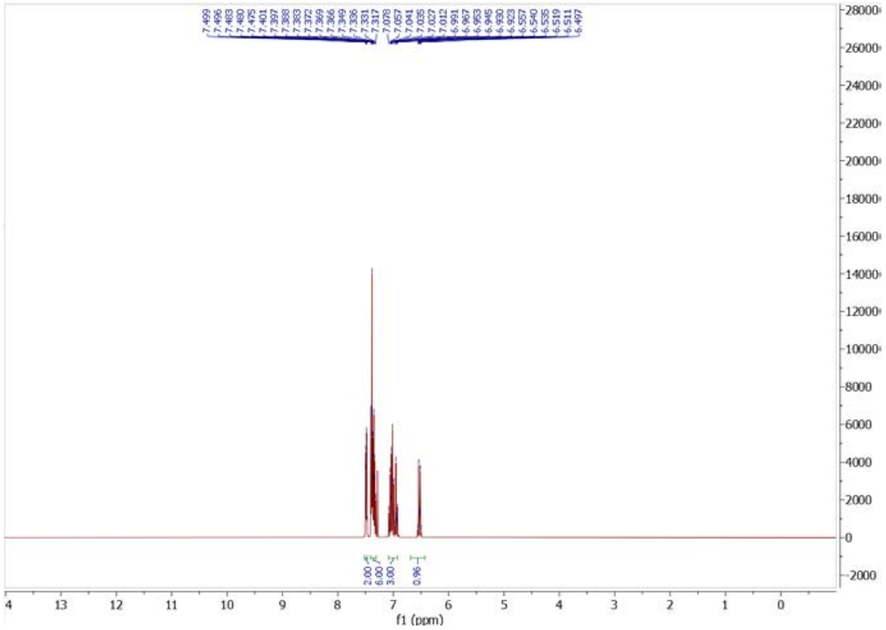

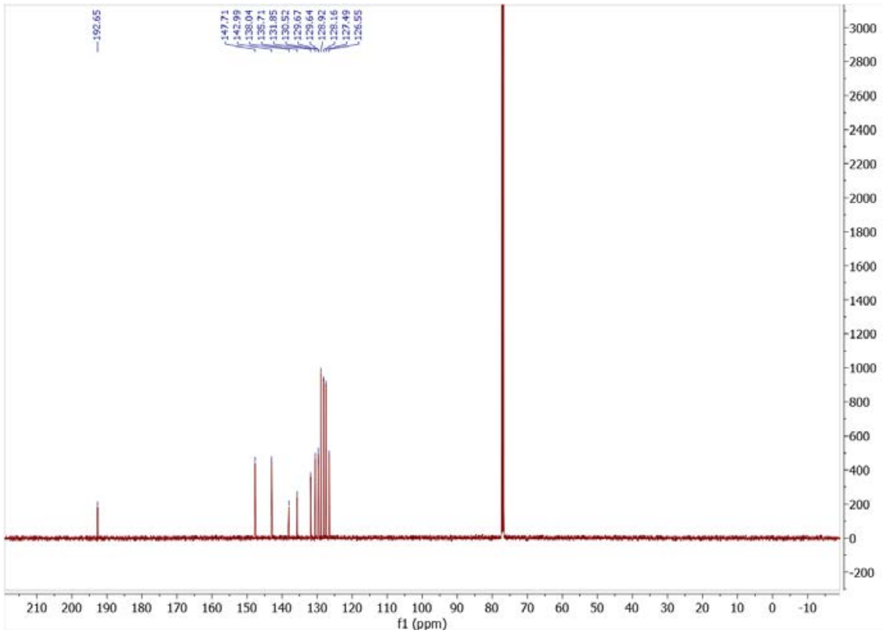

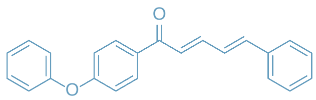
O
O

3g

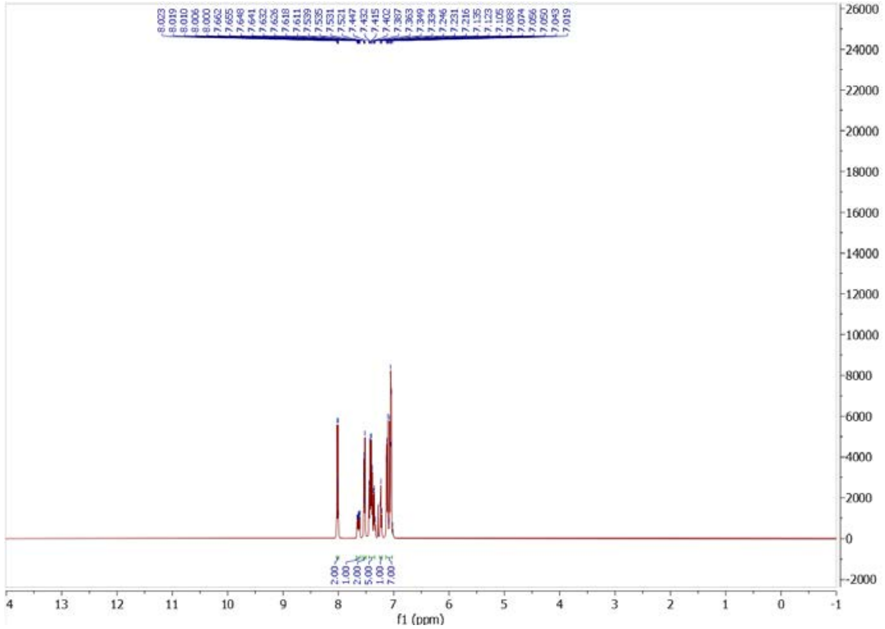
8.023 8.019 8.010 8.006 8.000 7.662 7.655 7.648 7.641 7.632 7.626 7.618 7.611 7.539 7.535 7.531 7.521 7.447 7.432 7.415 7.402 7.387 7.363 7.349 7.334 7.246 7.231 7.216 7.135 7.123 7.105 7.088 7.074 7.056 7.050 7.043 7.019
2.00 1.00 2.00 5.00 1.00 7.00
26000 24000 22000 20000 18000 16000 14000 12000 10000 8000 6000 4000 2000 0 -2000
4 13 12 11 10 9 8 7 6 5 4 3 2 1 0 -1
f1 (ppm)

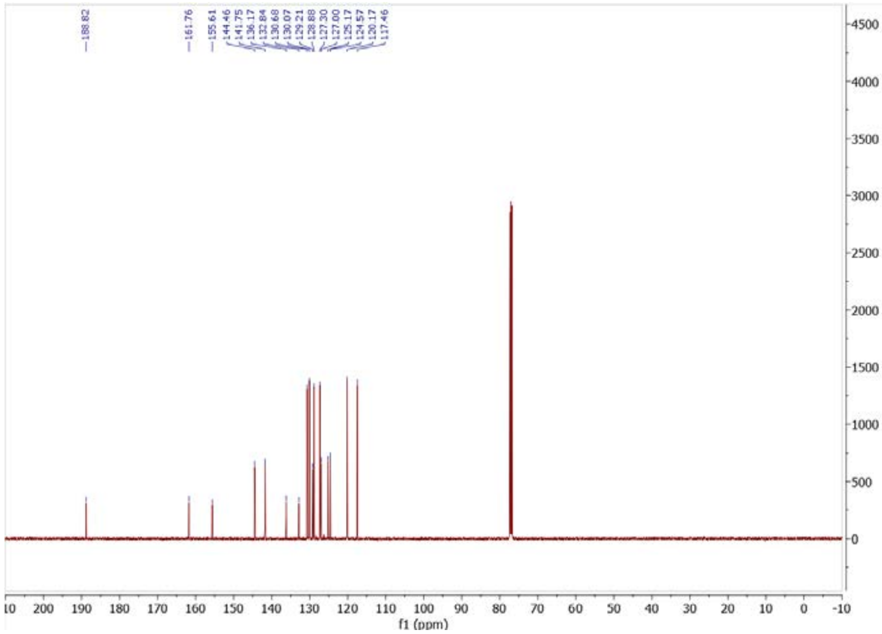
188.82
161.76 155.61 144.46 141.75 136.17 132.84 130.68 130.07 129.21 128.88 127.30 127.00 125.17 124.57 120.17 117.46
4500 4000 3500 3000 2500 2000 1500 1000 500 0
10 200 190 180 170 160 150 140 130 120 110 100 90 80 70 60 50 40 30 20 10 0 -10
f1 (ppm)

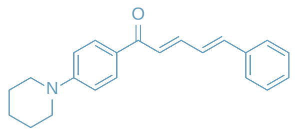

3h

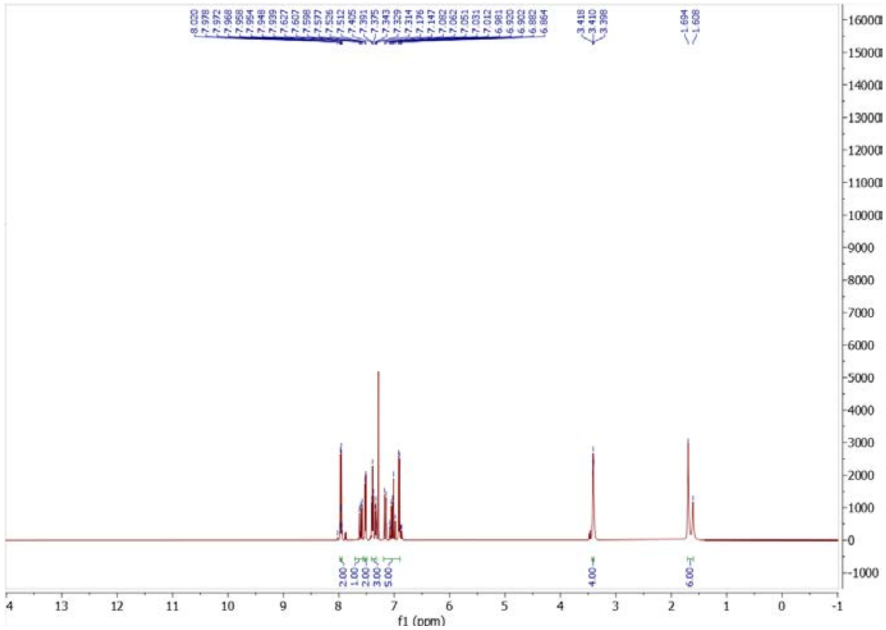

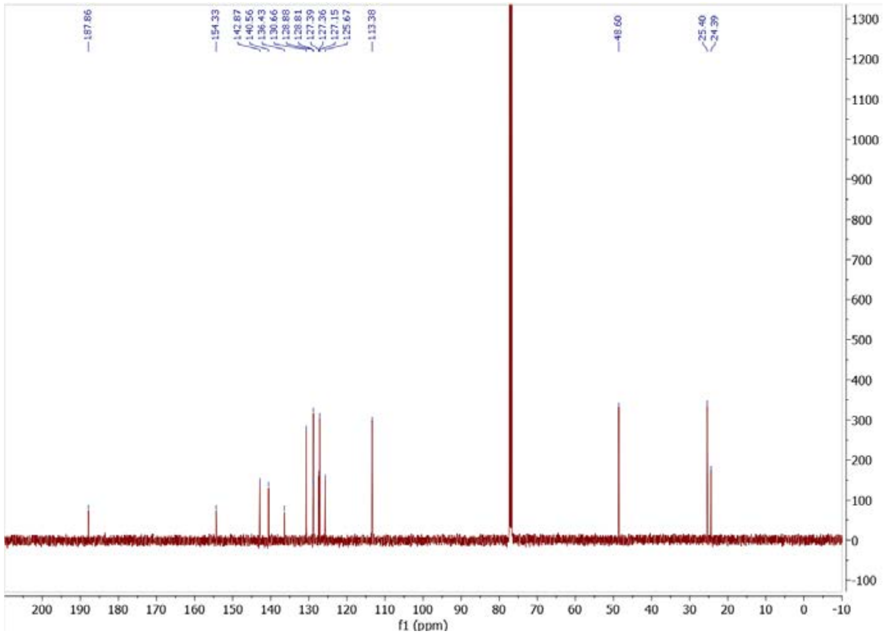

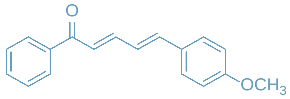

3i

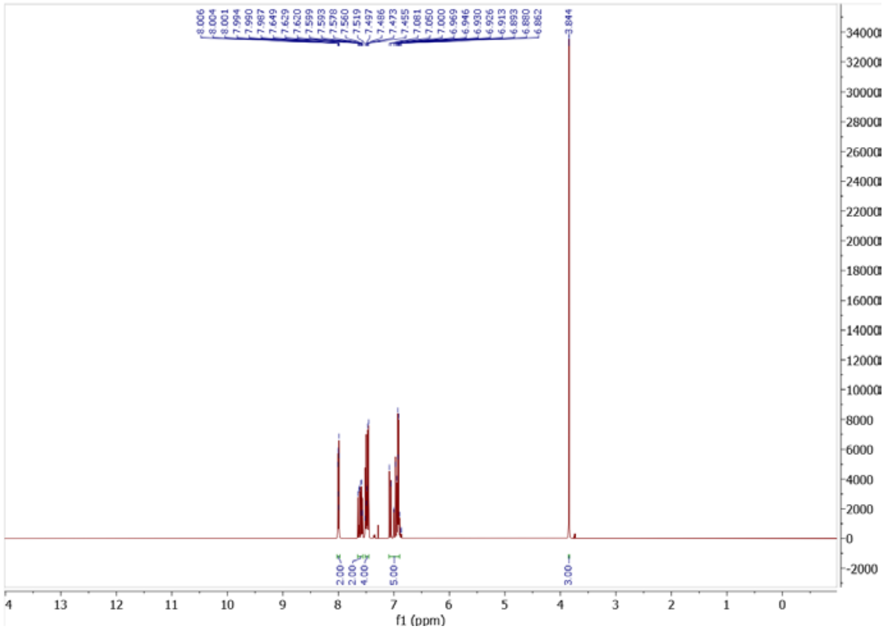

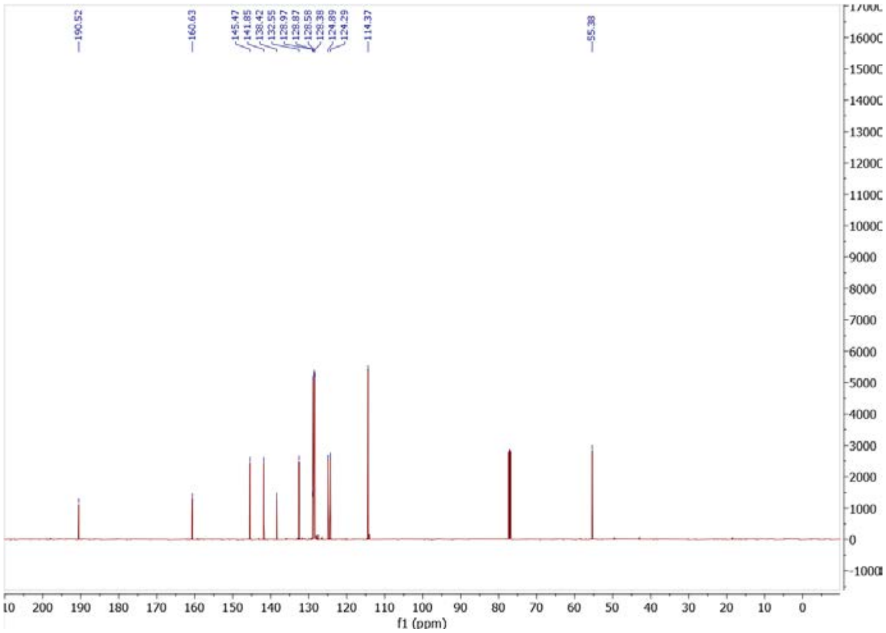

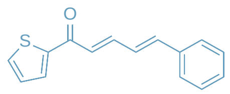
O
S

3j

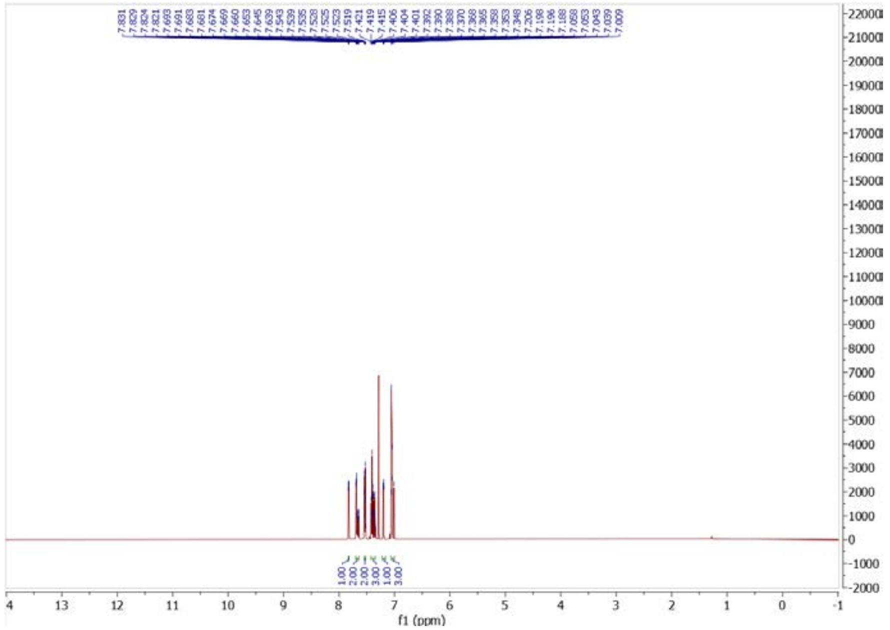
f1 (ppm)

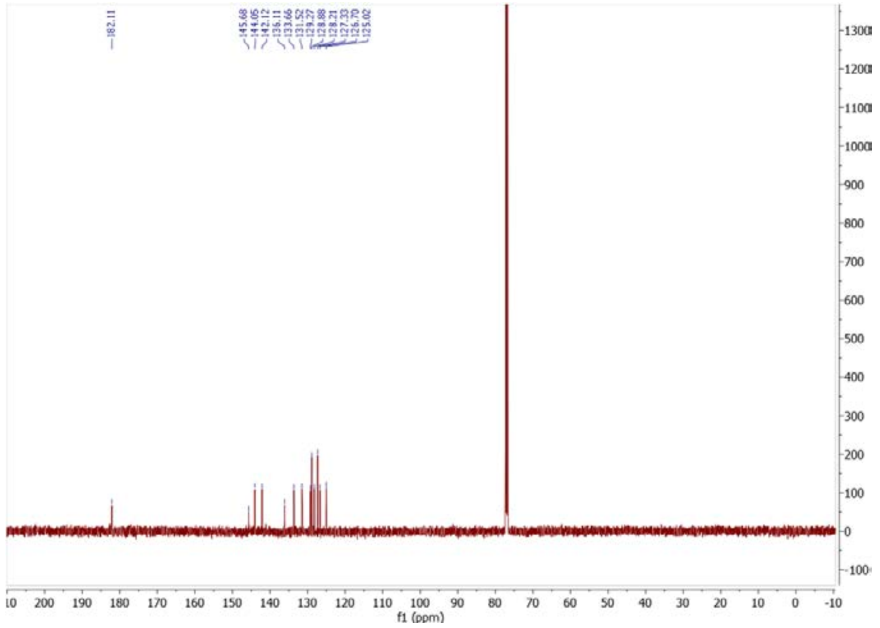
182.11
145.68
144.05
142.12
136.11
133.66
131.52
129.27
128.88
128.21
127.33
126.70
125.02
f1 (ppm)

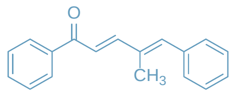
O
CH3

3k

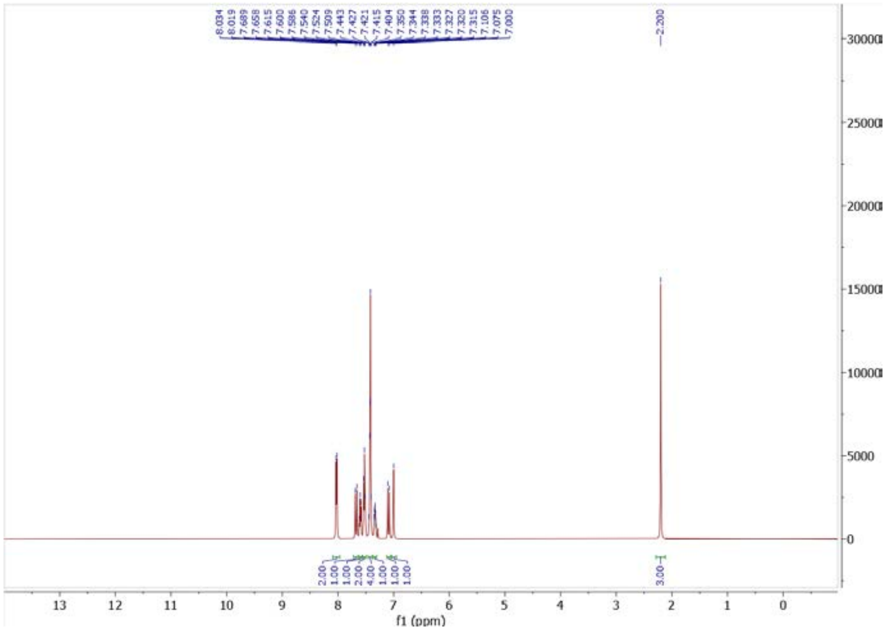
8.034
8.019
7.689
7.658
7.615
7.600
7.586
7.540
7.524
7.509
7.443
7.427
7.421
7.415
7.404
7.350
7.344
7.338
7.333
7.327
7.320
7.315
7.106
7.075
7.000
2.200
2.00
1.00
2.00
4.00
1.00
1.00
1.00
3.00
30000
25000
20000
15000
10000
5000
0
13 12 11 10 9 8 7 6 5 4 3 2 1 0
f1 (ppm)

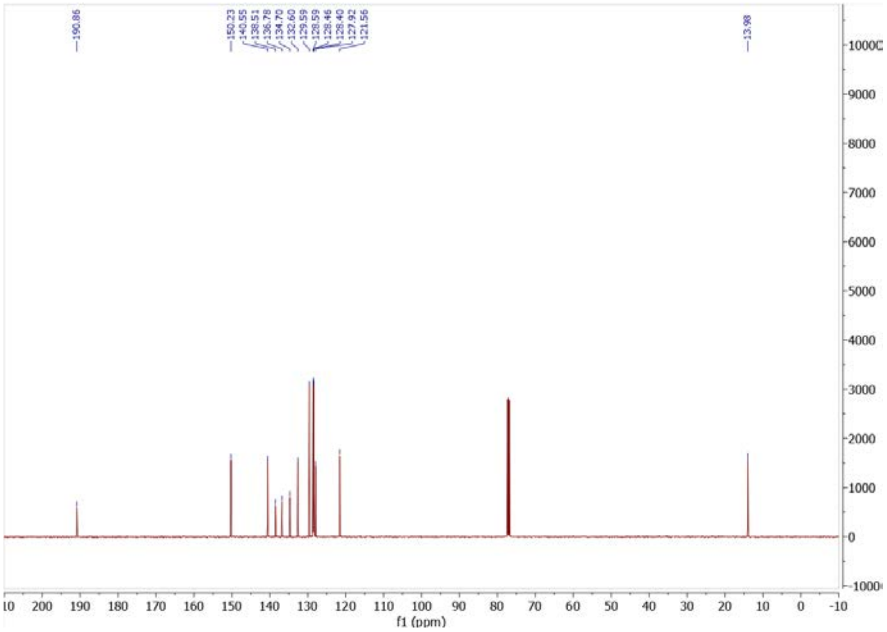
190.86
150.23
140.55
138.51
136.78
134.70
132.60
129.59
128.59
128.46
128.40
127.92
121.56
13.98
9000
8000
7000
6000
5000
4000
3000
2000
1000
0
200 190 180 170 160 150 140 130 120 110 100 90 80 70 60 50 40 30 20 10 0 -10
f1 (ppm)

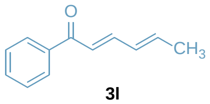
O
CH3

3l

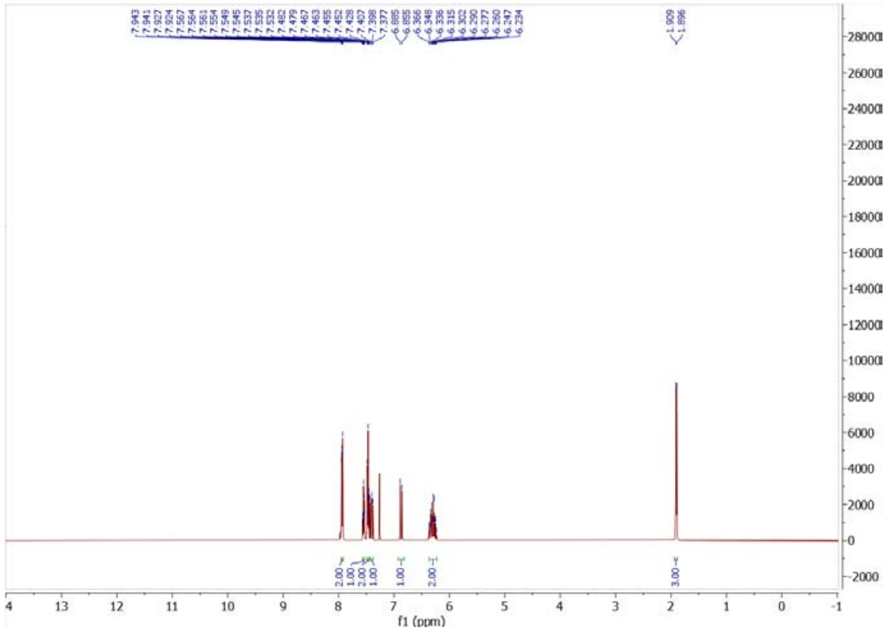
7.943
7.941
7.927
7.924
7.567
7.564
7.561
7.554
7.549
7.545
7.537
7.535
7.532
7.482
7.479
7.467
7.463
7.455
7.452
7.428
7.407
7.398
7.377
6.885
6.855
6.366
6.348
6.336
6.315
6.302
6.290
6.277
6.260
6.247
6.234
1.909
1.896
2.00
1.00
2.00
1.00
1.00
2.00
3.00
f1 (ppm)

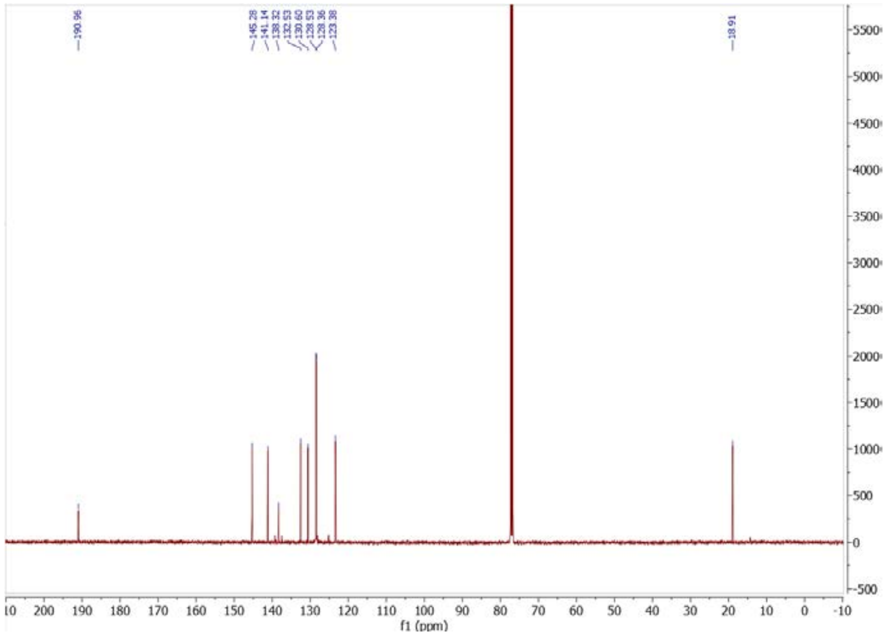
190.96
145.28
141.14
138.32
132.53
130.60
128.53
128.36
123.38
18.91
f1 (ppm)

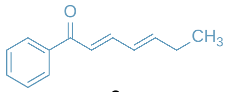
O
CH_3

3m

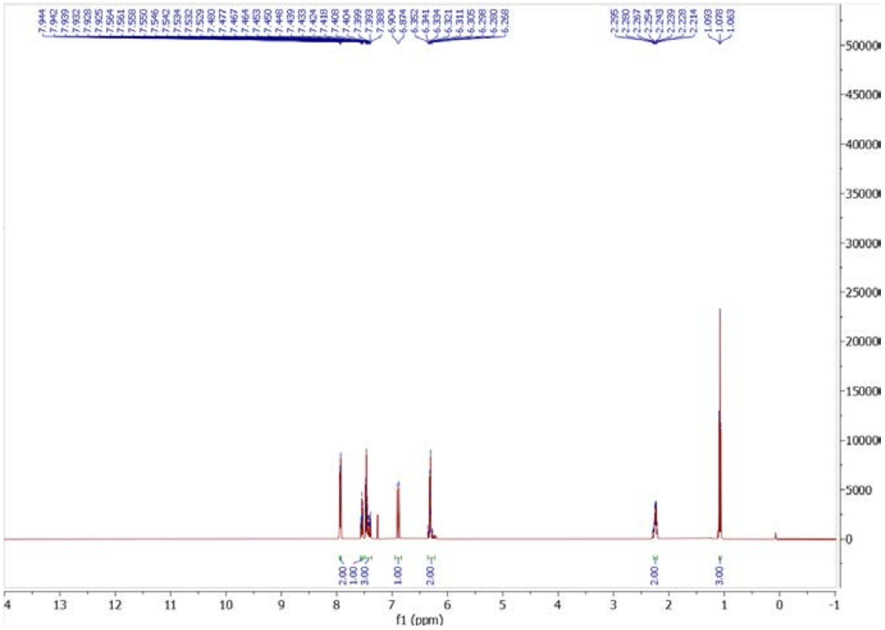
f1 (ppm)

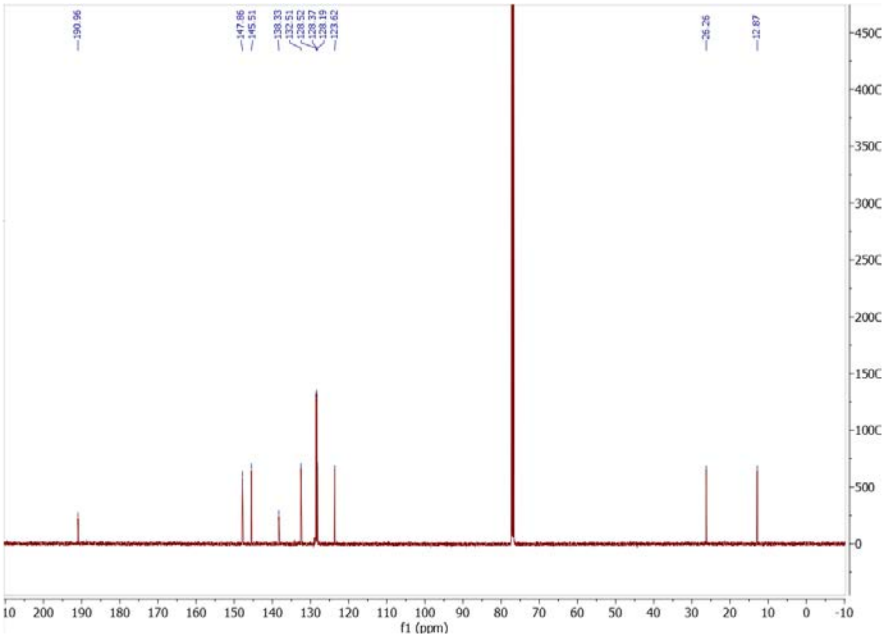
190.96
147.86
145.51
138.33
132.51
128.52
128.37
128.19
123.62
26.26
12.87
f1 (ppm)

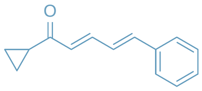

3n

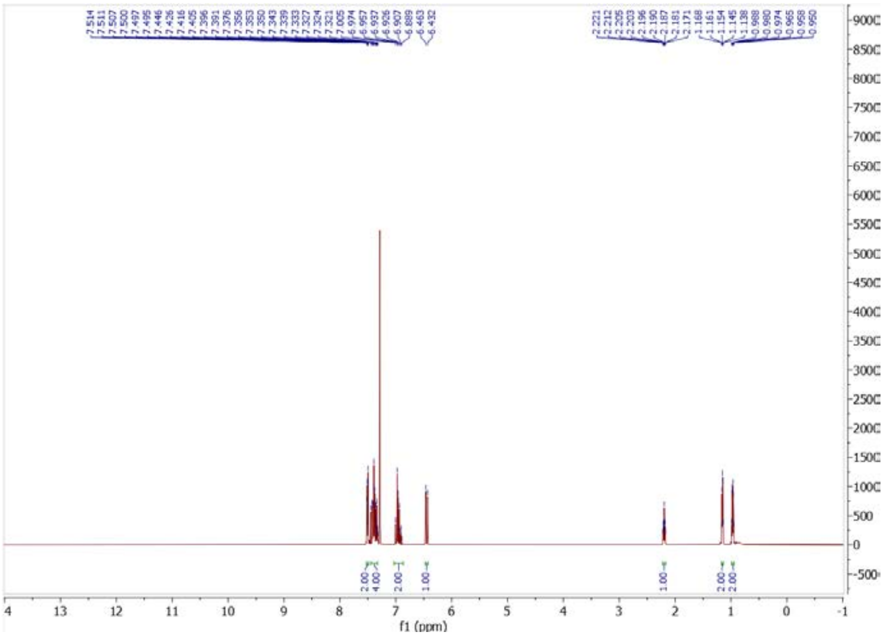

7.514
7.511
7.507
7.500
7.497
7.495
7.446
7.426
7.416
7.405
7.396
7.391
7.376
7.356
7.353
7.350
7.343
7.339
7.333
7.327
7.324
7.321
7.005
6.974
6.957
6.937
6.926
6.907
6.899
6.463
6.432
2.221
2.212
2.205
2.203
2.196
2.190
2.187
2.181
2.171
1.168
1.161
1.154
1.145
1.138
0.988
0.980
0.974
0.965
0.958
0.950
2.00
4.00
2.00
1.00
1.00
2.00
2.00
f1 (ppm)

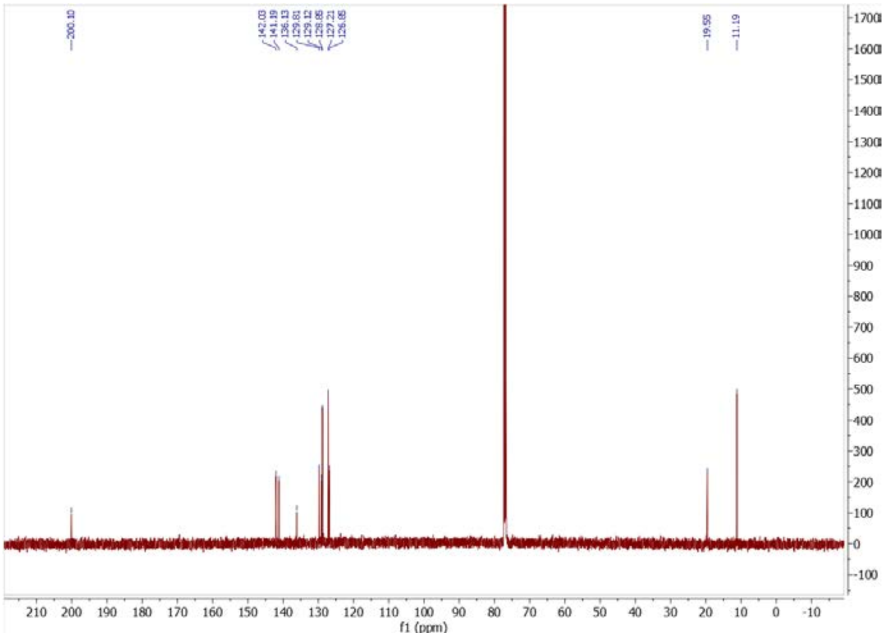

200.10
142.03
141.19
136.13
129.81
129.12
128.85
127.21
126.85
19.55
11.19
f1 (ppm)

9.2 1,6-conjugate addition products.

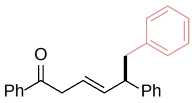

4a

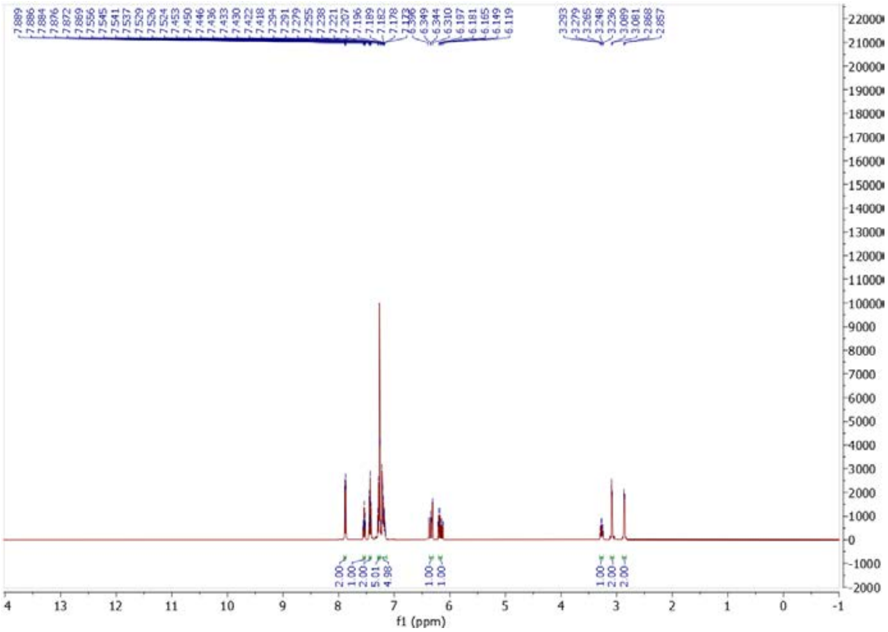

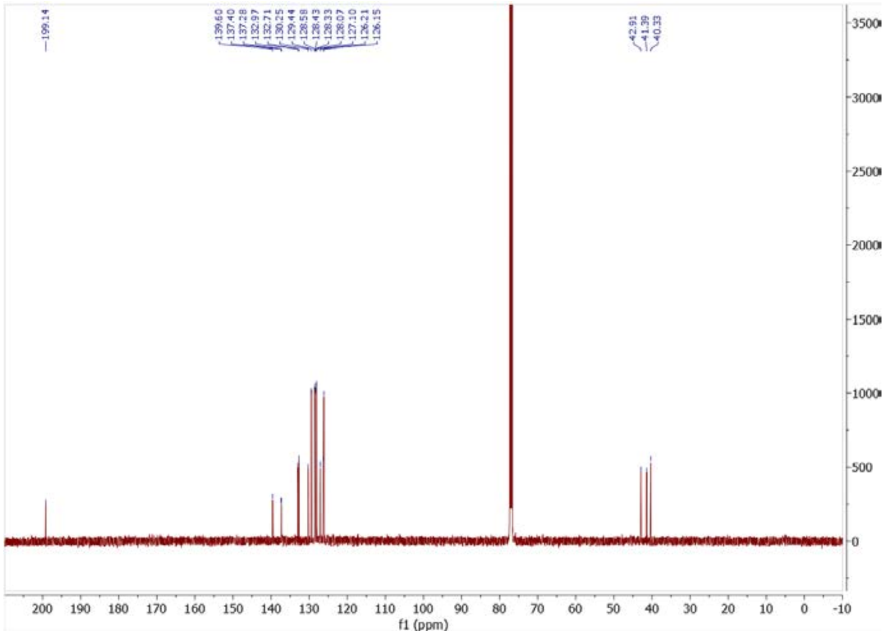

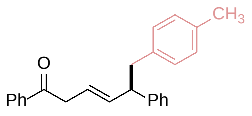

5

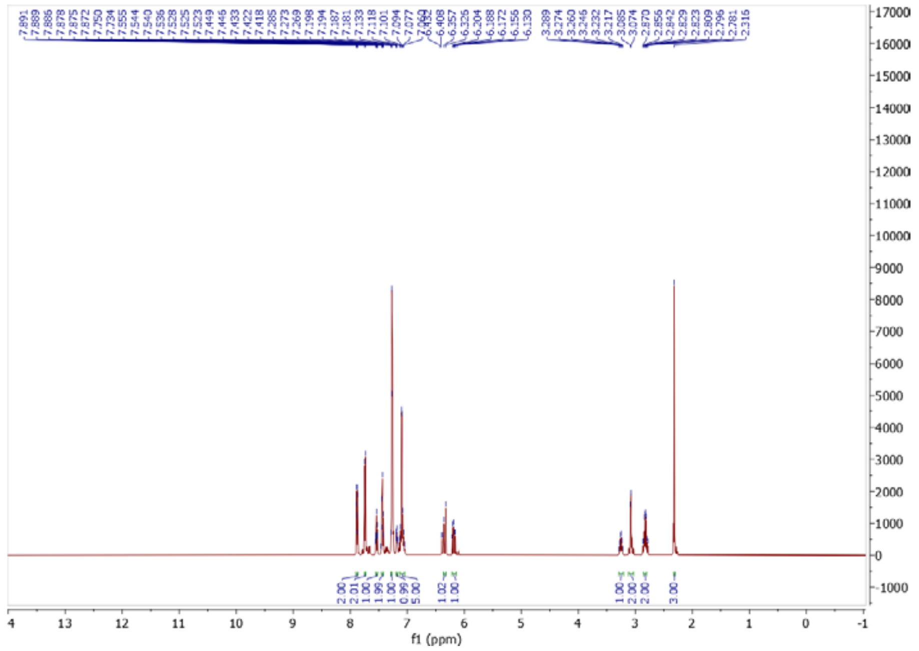

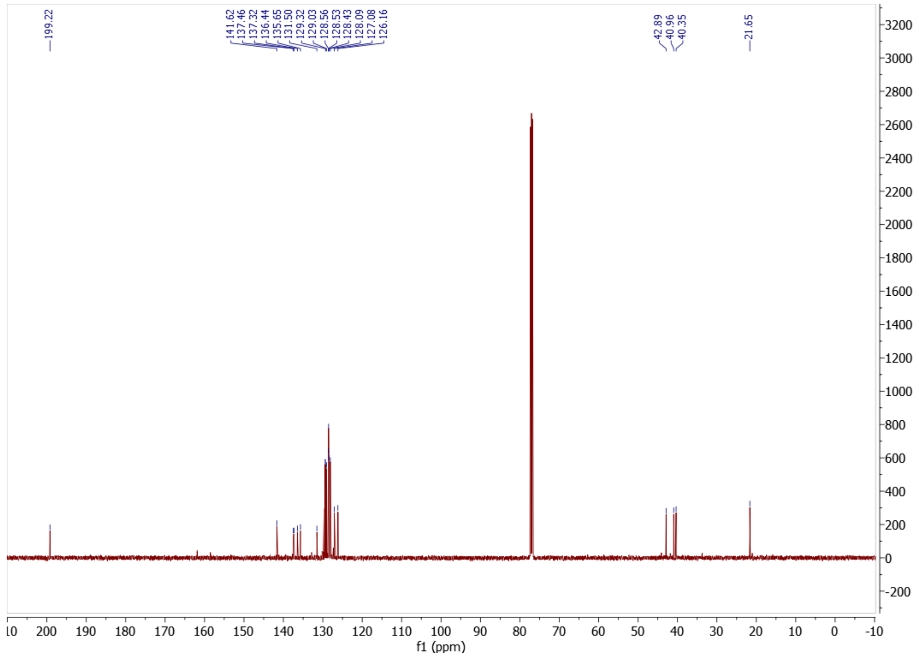

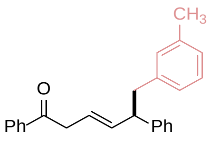

6

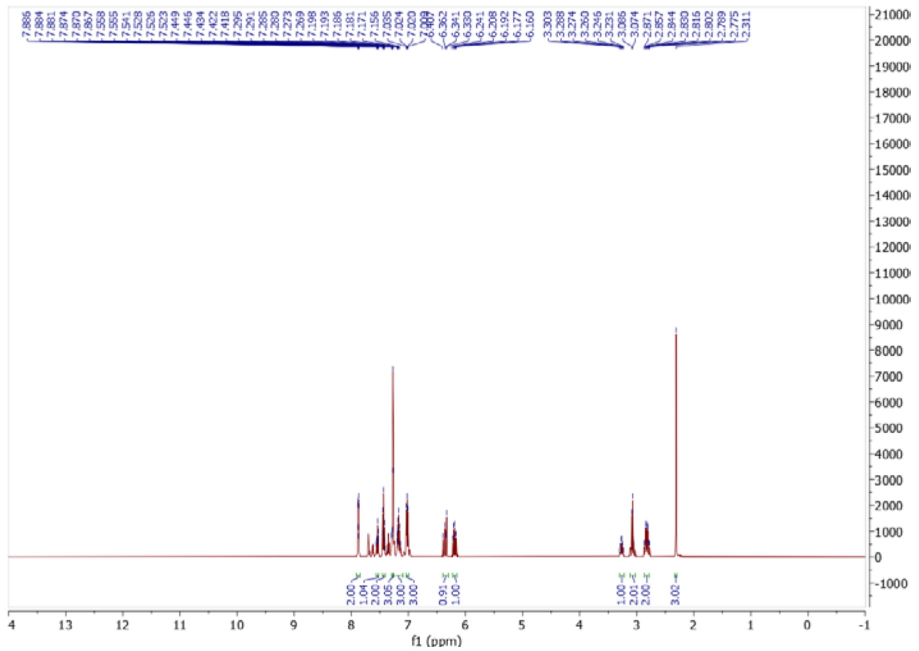

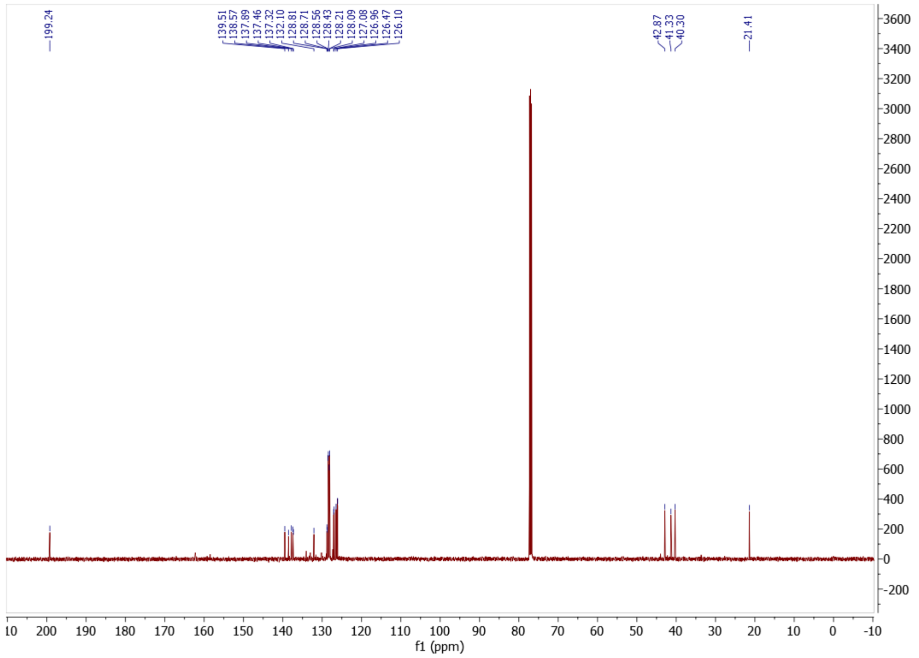

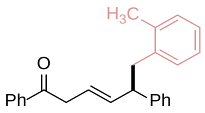

7

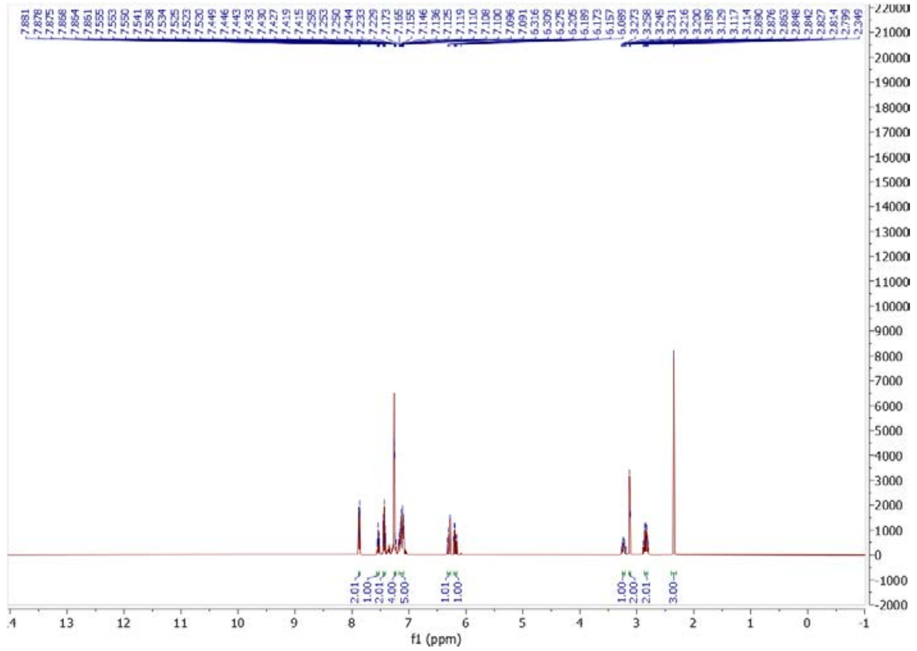

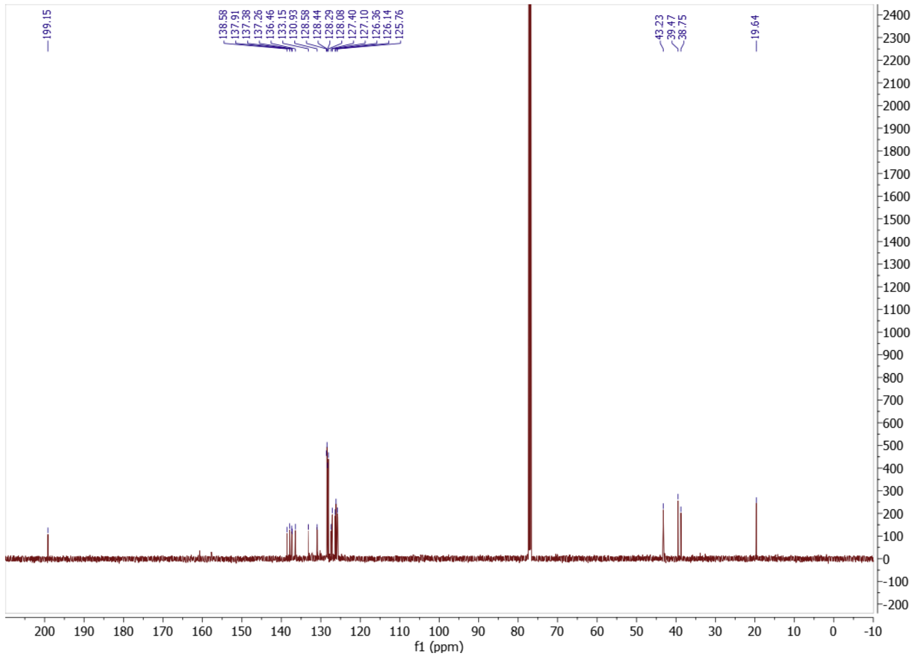

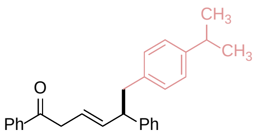

8

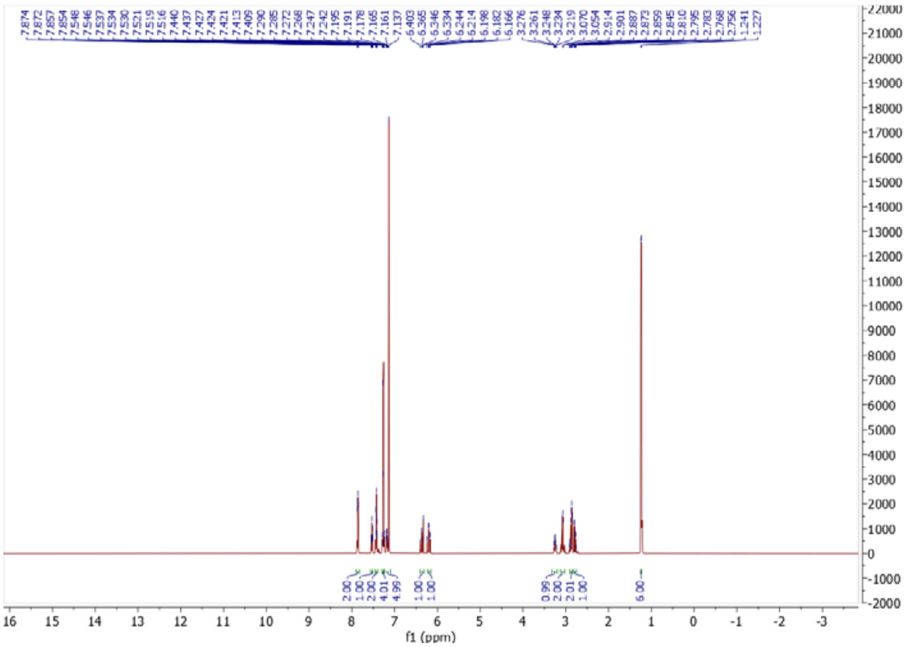

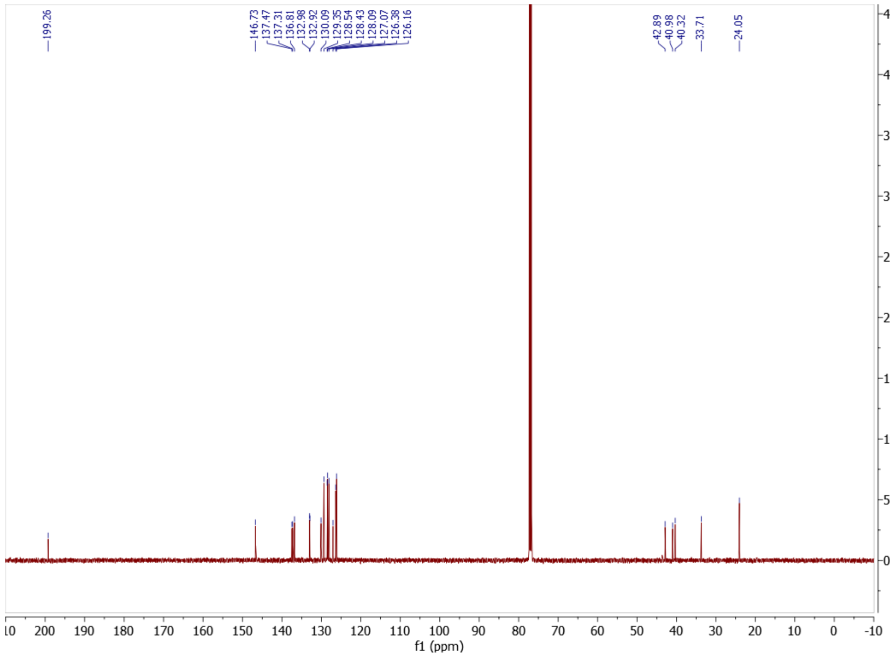

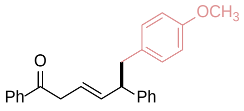

OCH3
O
Ph
Ph

9

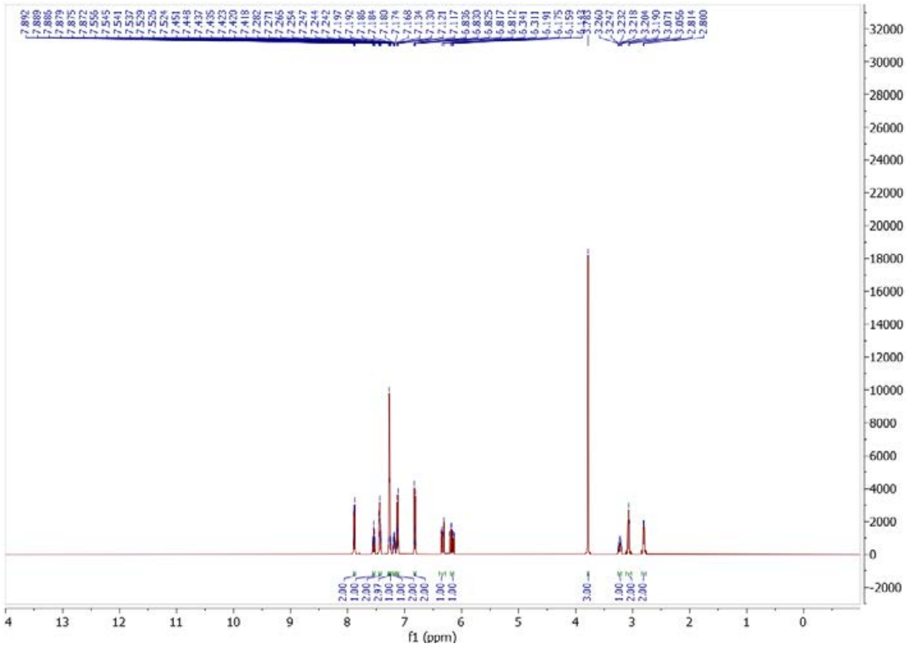

f1 (ppm)

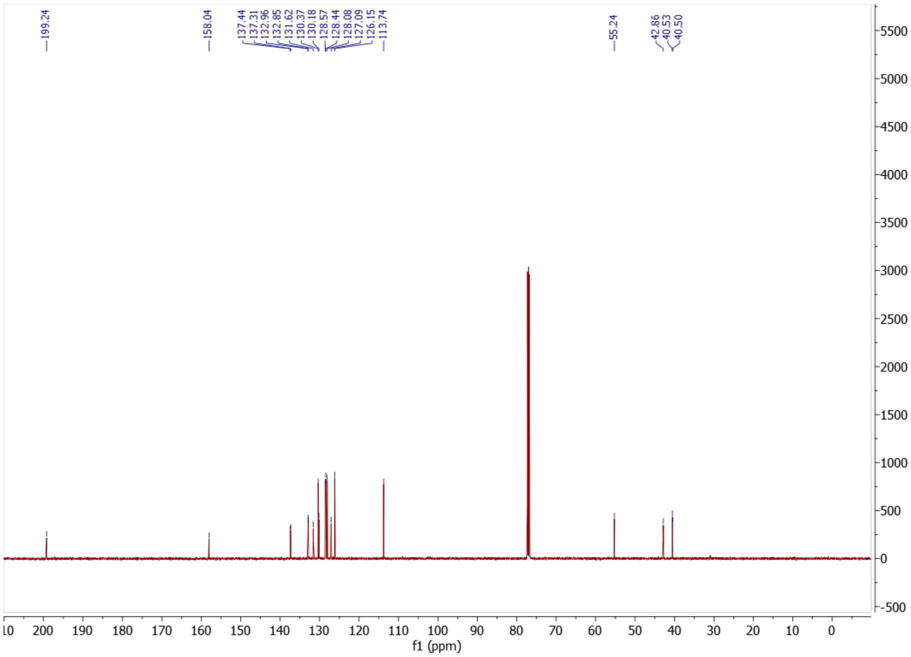

199.24
158.04
137.44
137.31
132.96
132.85
131.62
130.37
130.18
128.57
128.44
128.08
127.09
126.15
113.74
55.24
42.86
40.53
40.50
f1 (ppm)

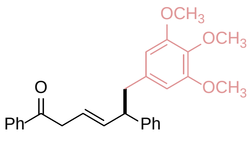

10

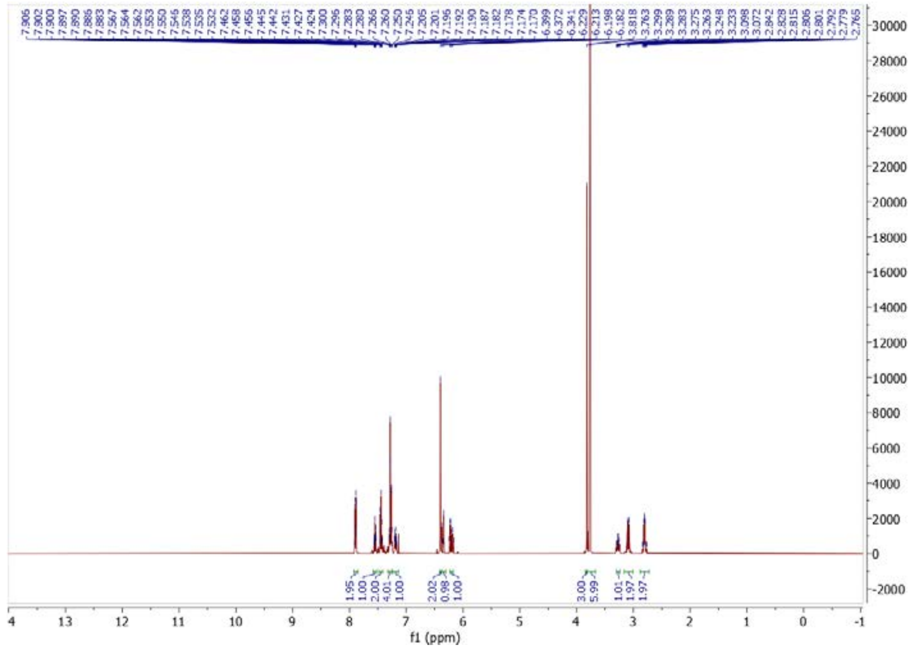

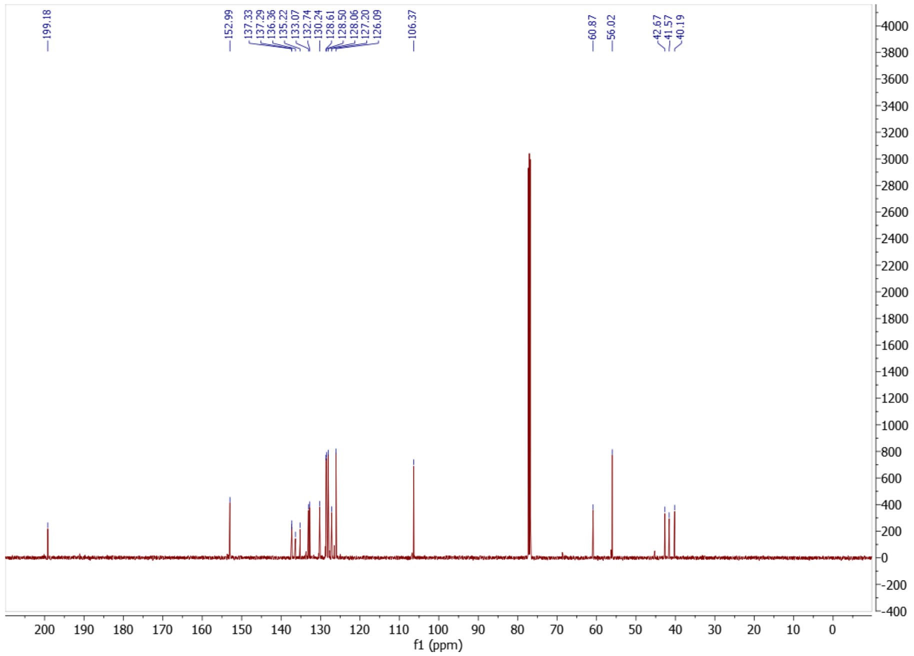

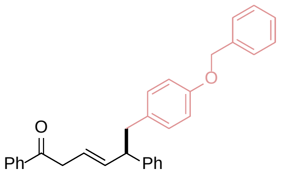

11

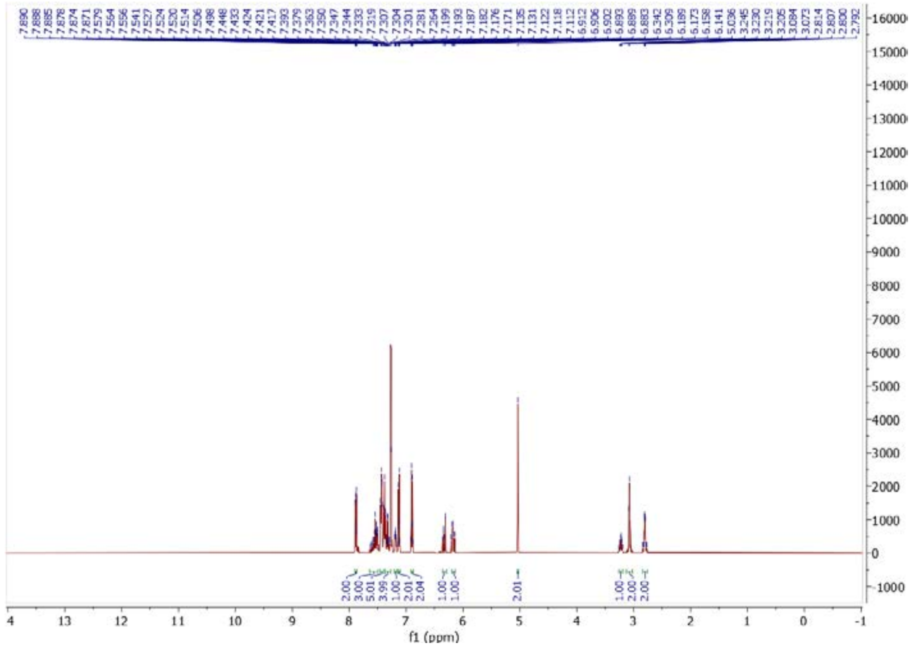

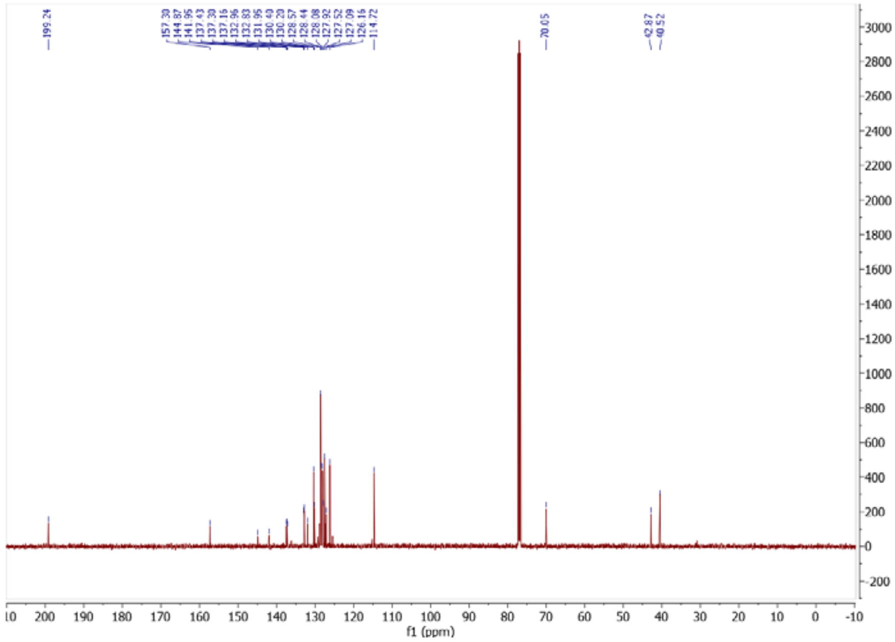

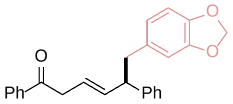

O
Ph
Ph

12

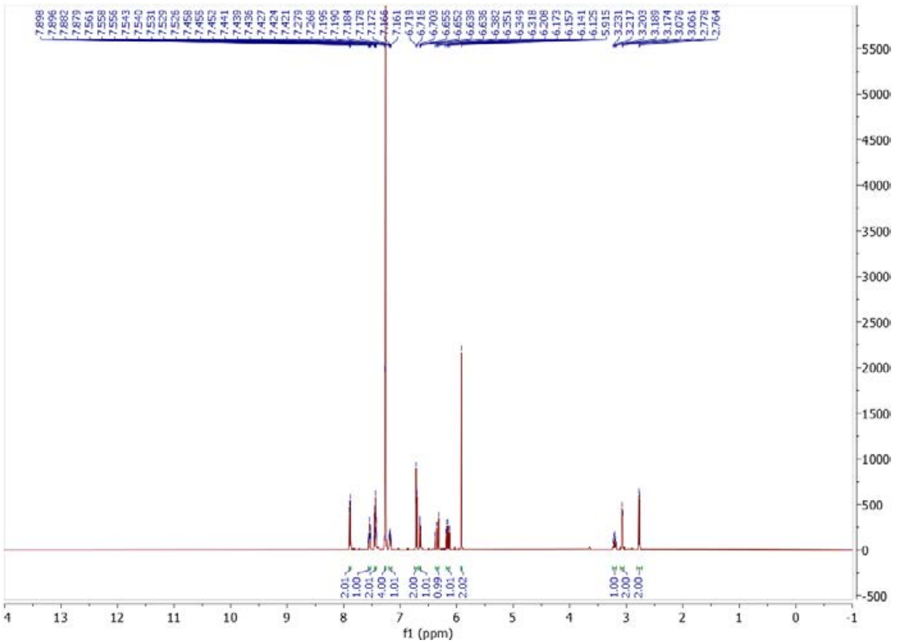

7.898 7.896 7.882 7.879 7.561 7.558 7.556 7.543 7.540 7.531 7.529 7.526 7.458 7.455 7.452 7.441 7.439 7.436 7.427 7.424 7.421 7.279 7.268 7.195 7.190 7.184 7.178 7.172 7.166 7.161 6.719 6.716 6.703 6.655 6.652 6.639 6.636 6.382 6.351 6.349 6.318 6.208 6.173 6.157 6.141 6.125 5.915 3.231 3.217 3.203 3.189 3.174 3.076 3.061 2.778 2.764
2.01 1.00 2.01 4.00 1.01 2.00 1.01 0.99 1.01 2.02 1.00 2.00 2.00
f1 (ppm)

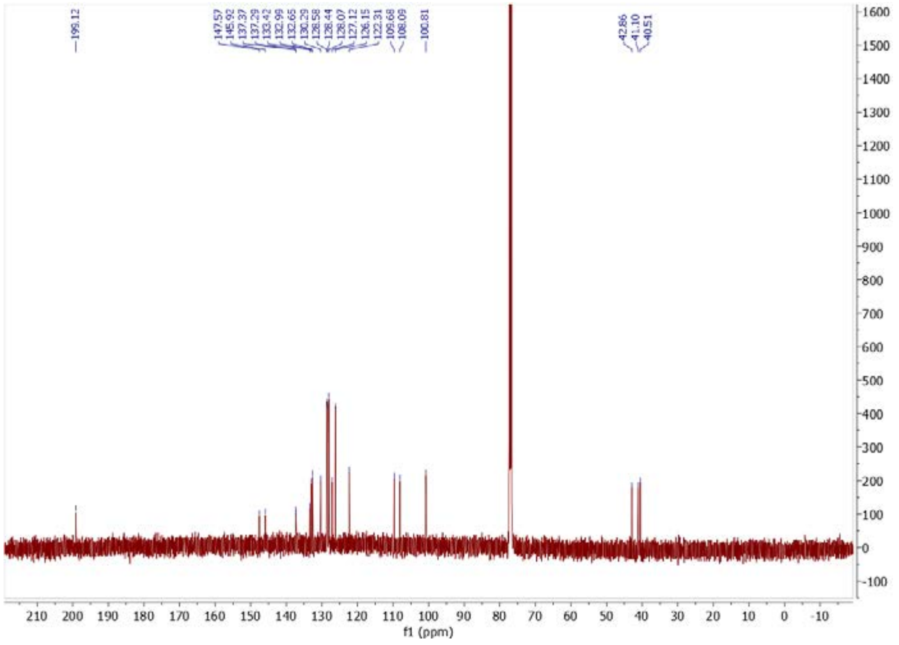

199.12
147.57 145.92 137.37 137.29 133.42 132.99 132.65 130.29 128.58 128.44 128.07 127.12 126.15 122.31 109.68 108.09
100.81
42.86 41.10 40.51
f1 (ppm)

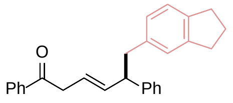
O
Ph
Ph

13

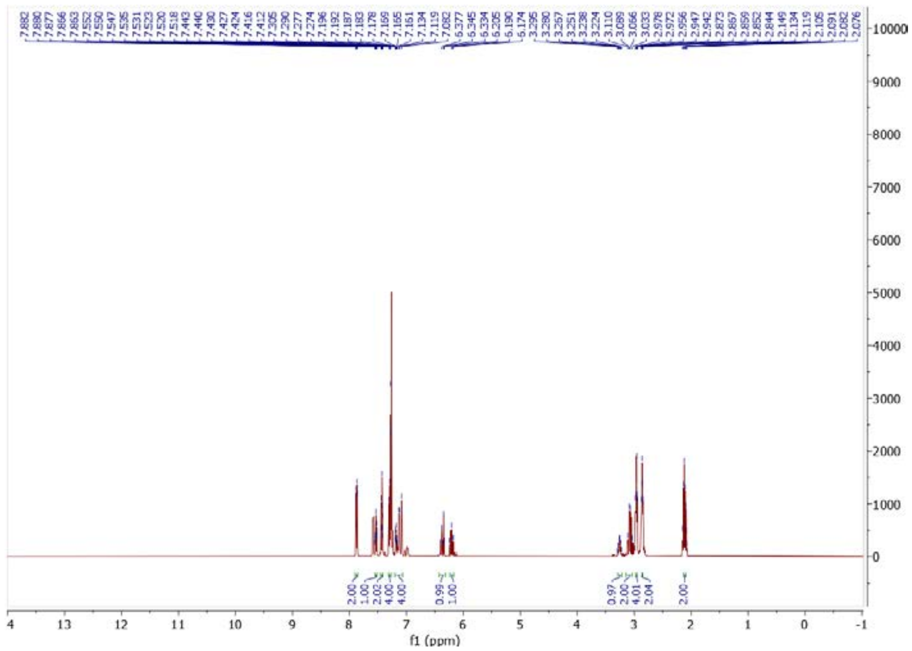
7.882 7.880 7.877 7.866 7.863 7.552 7.550 7.547 7.535 7.531 7.523 7.520 7.518 7.443 7.440 7.430 7.427 7.424 7.416 7.412 7.306 7.290 7.277 7.274 7.196 7.192 7.187 7.183 7.178 7.169 7.165 7.151 7.134 7.119 7.082 6.377 6.345 6.334 6.205 6.190 6.174 3.295 3.280 3.257 3.251 3.238 3.224 3.110 3.089 3.056 3.033 2.976 2.972 2.956 2.947 2.942 2.873 2.867 2.859 2.852 2.844 2.149 2.134 2.119 2.105 2.091 2.082 2.076
2.00 1.00 2.02 4.00 4.00 0.99 1.00 0.97 2.00 4.01 2.04 2.00
f1 (ppm)

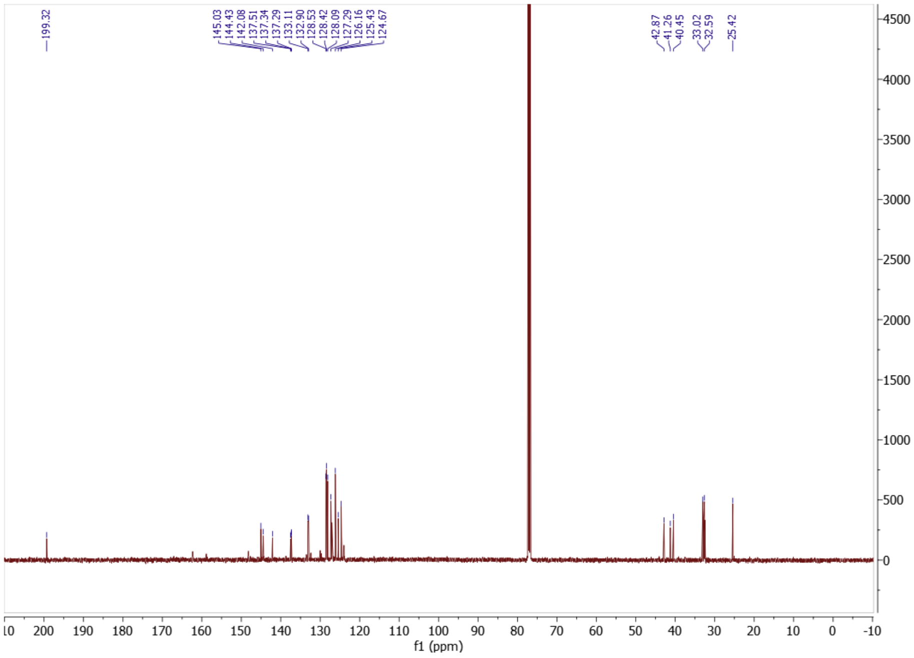
199.32
145.03 144.43 142.08 137.51 137.34 137.29 133.11 132.90 128.53 128.42 128.09 127.29 126.16 125.43 124.67
42.87 41.26 40.45 33.02 32.59 25.42
f1 (ppm)

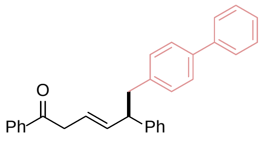

14

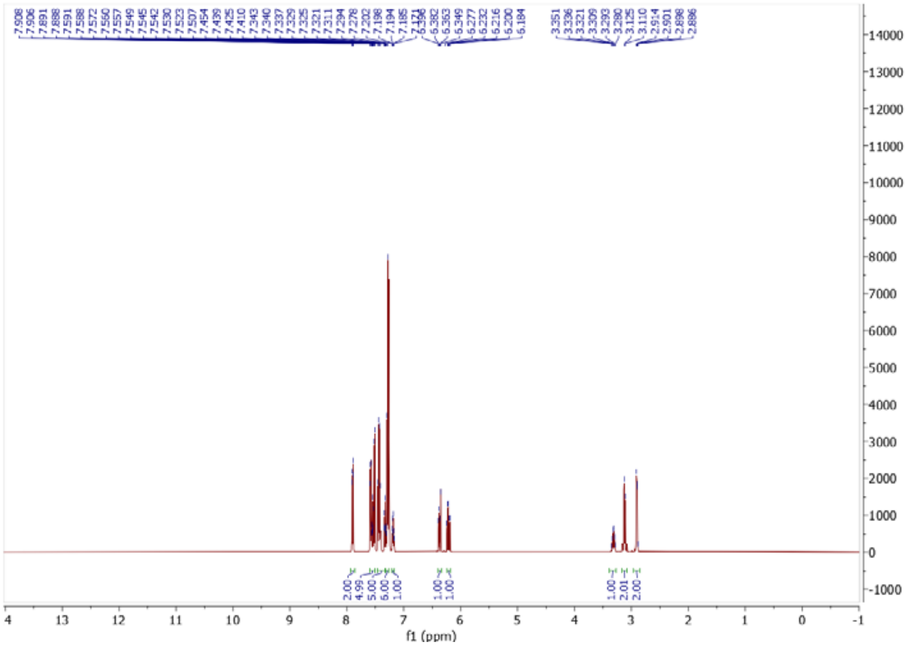

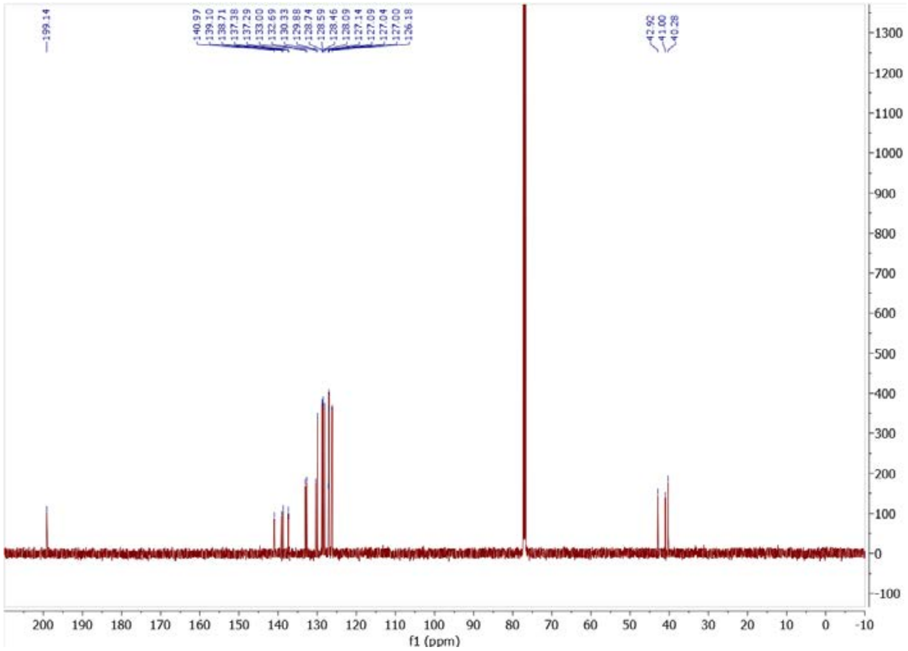

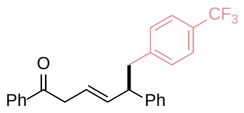
O
Ph
Ph
CF_3

15

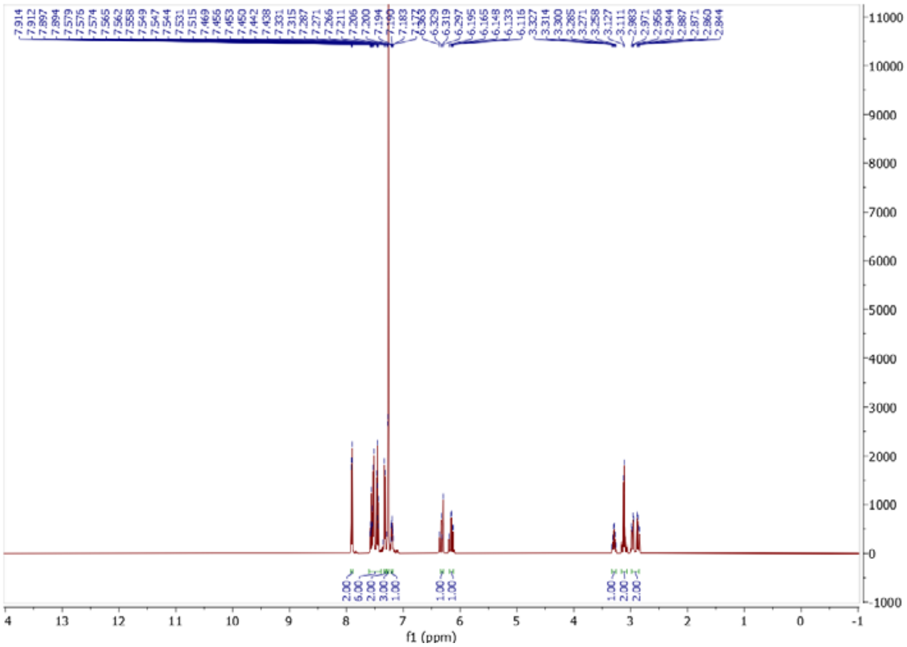
7.914
7.912
7.897
7.894
7.579
7.576
7.574
7.565
7.562
7.558
7.549
7.547
7.544
7.531
7.515
7.469
7.456
7.453
7.450
7.442
7.438
7.331
7.315
7.287
7.271
7.266
7.211
7.206
7.200
7.194
7.190
7.183
6.329
6.319
6.297
6.195
6.165
6.148
6.133
6.116
3.327
3.314
3.300
3.285
3.271
3.258
3.127
3.111
2.983
2.971
2.956
2.944
2.887
2.871
2.860
2.844
2.00
6.00
2.00
3.00
1.00
1.00
1.00
1.00
2.00
2.00
f1 (ppm)

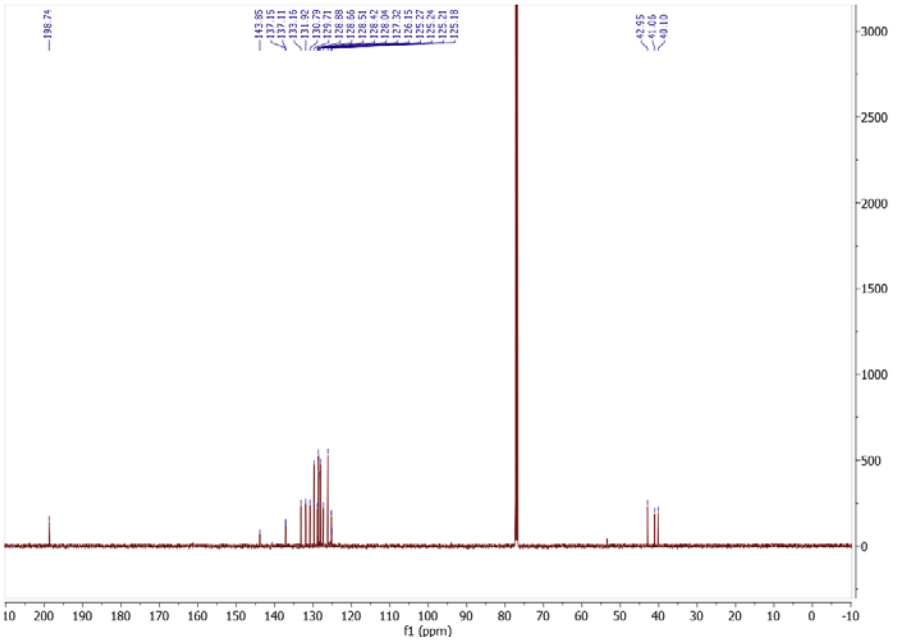
198.74
143.85
137.15
137.11
133.16
131.92
130.79
129.71
128.88
128.66
128.51
128.42
128.04
127.32
126.15
125.27
125.24
125.21
125.18
42.95
41.05
40.10
f1 (ppm)

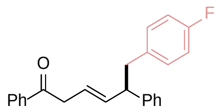

16

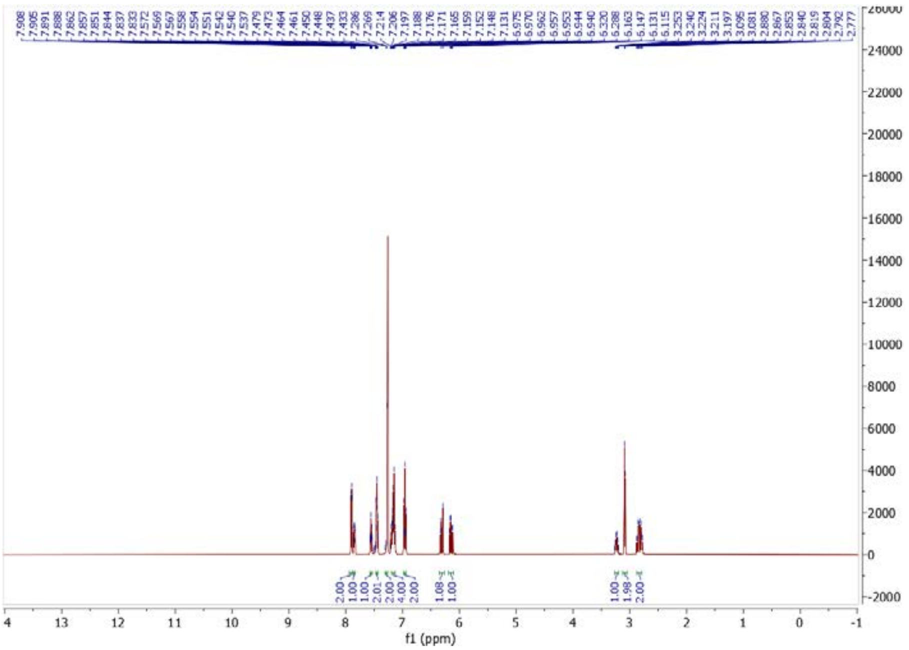

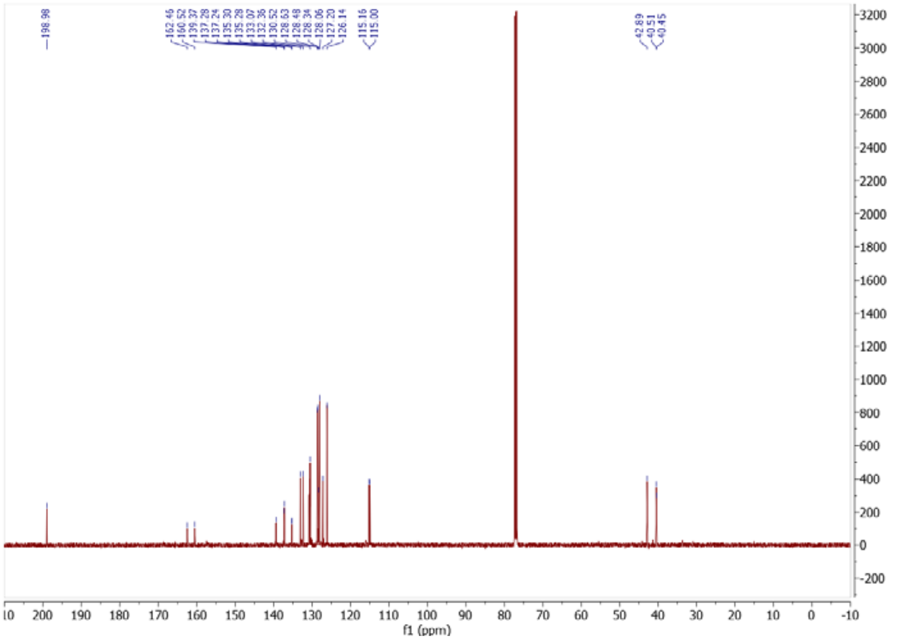

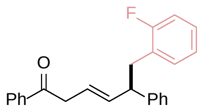

17

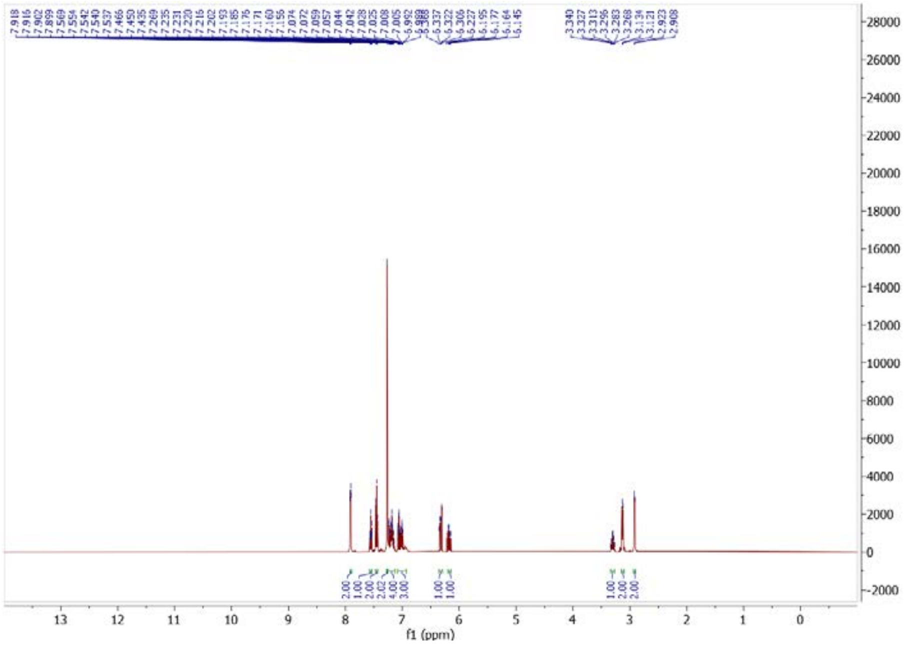

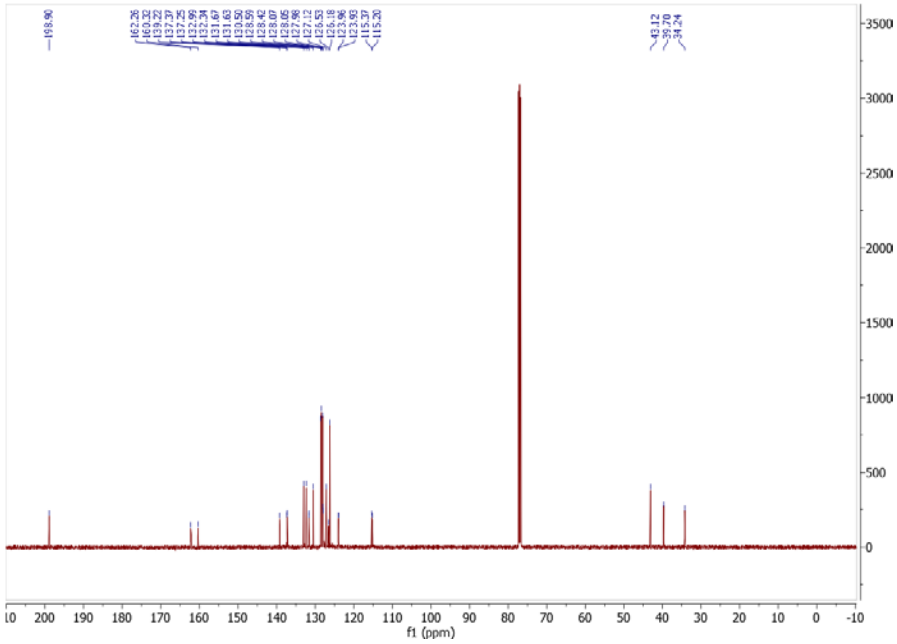

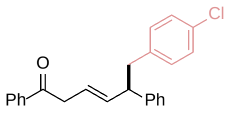
Cl
O
Ph
Ph

18

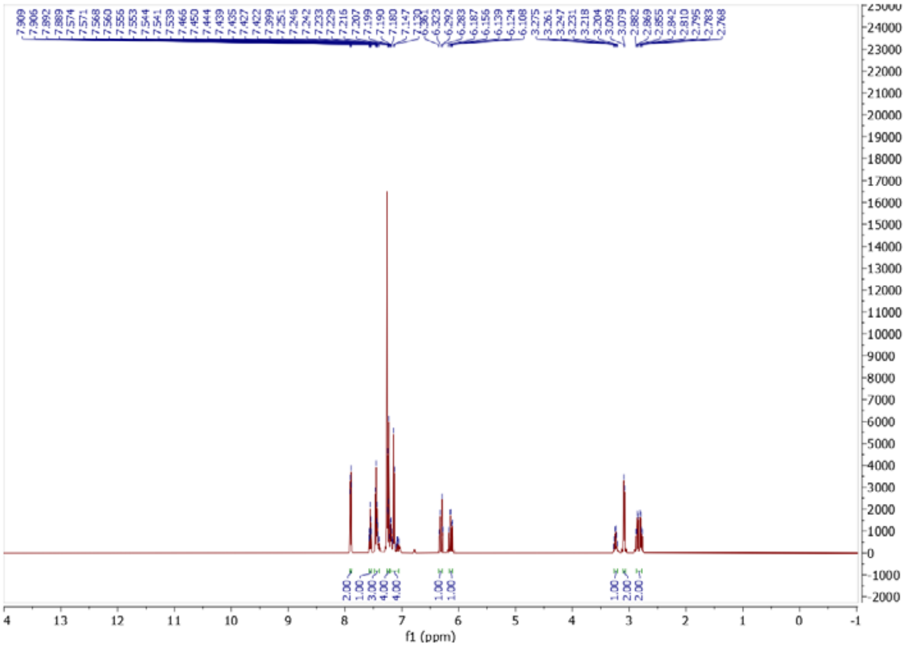
7.909
7.906
7.892
7.889
7.574
7.571
7.568
7.560
7.556
7.553
7.544
7.541
7.539
7.466
7.450
7.444
7.439
7.435
7.427
7.422
7.399
7.251
7.246
7.242
7.233
7.229
7.216
7.207
7.199
7.190
7.180
7.147
7.130
6.361
6.323
6.292
6.283
6.187
6.156
6.139
6.124
6.108
3.275
3.261
3.247
3.231
3.218
3.204
3.093
3.079
2.882
2.869
2.855
2.842
2.810
2.795
2.783
2.768
2.00
1.00
3.00
4.00
4.00
1.00
1.00
1.00
2.00
2.00
f1 (ppm)

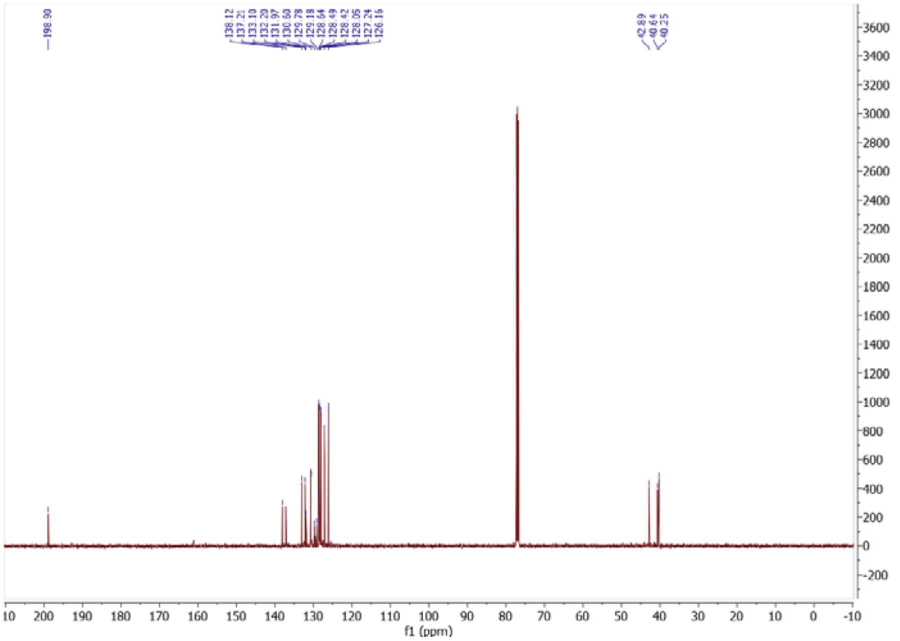
198.90
138.12
137.21
133.10
132.20
131.97
130.60
129.78
129.18
128.64
128.49
128.42
128.05
127.24
126.16
42.89
40.64
40.25
f1 (ppm)

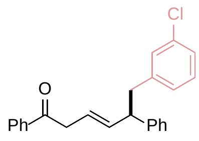
Cl
O
Ph
Ph

19

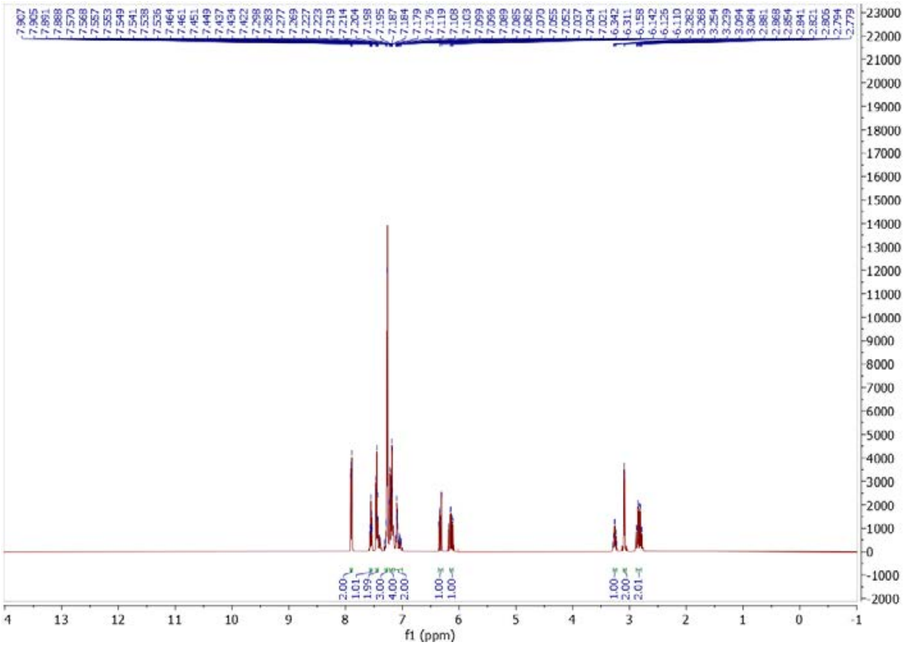
f1 (ppm)

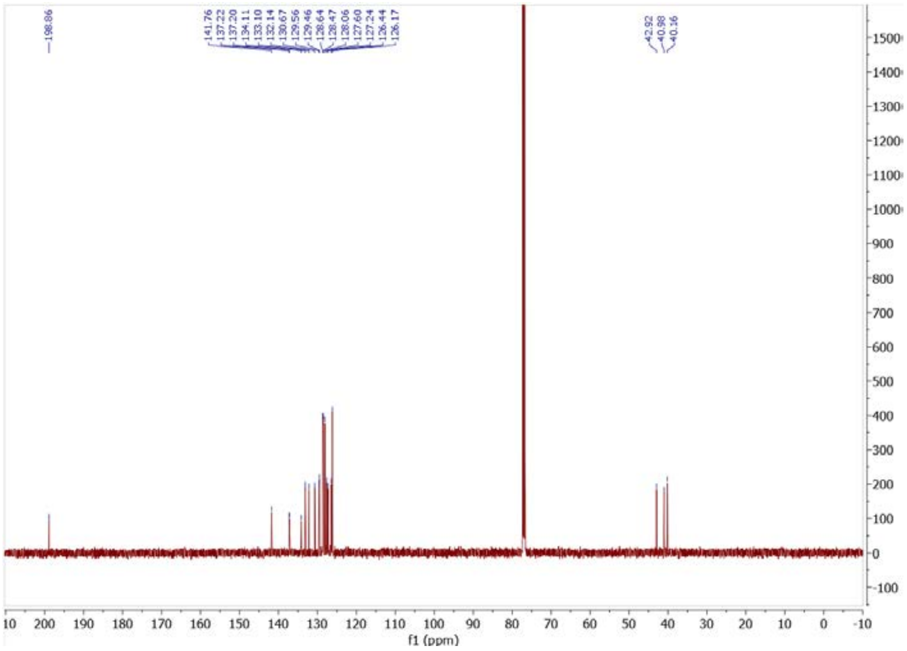
198.86
141.76
137.22
137.20
134.11
133.10
132.14
130.67
129.56
129.46
128.64
128.47
128.06
127.60
127.24
126.44
126.17
42.92
40.98
40.16
f1 (ppm)

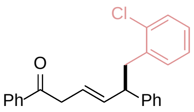

20

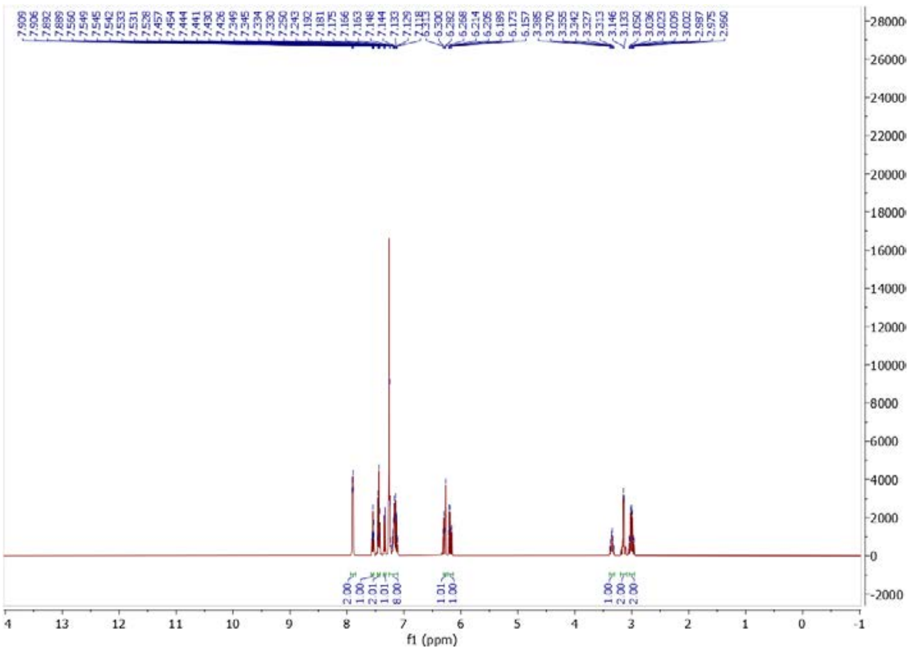

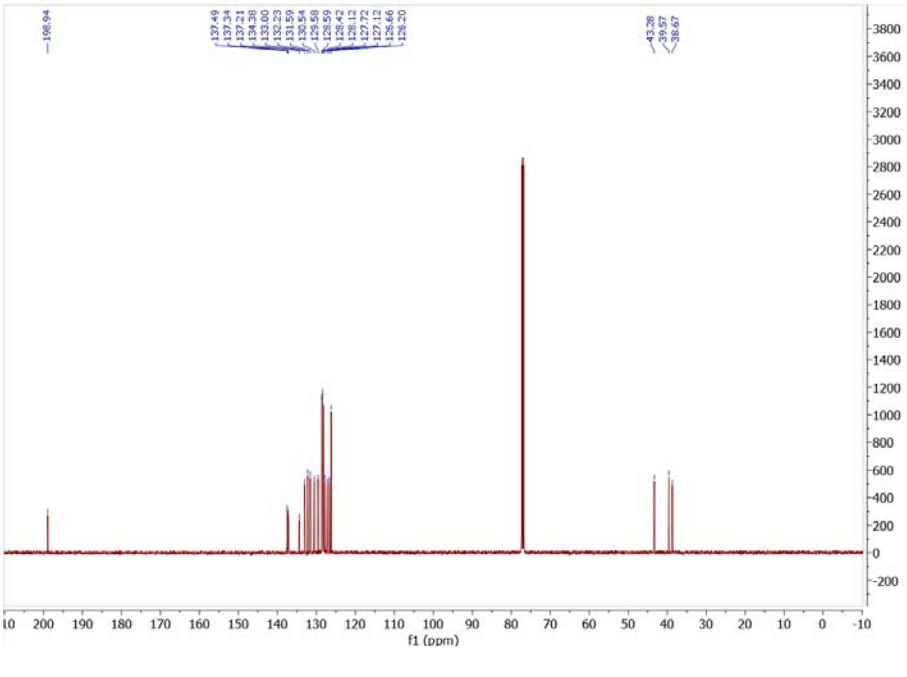

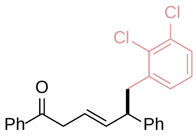

21

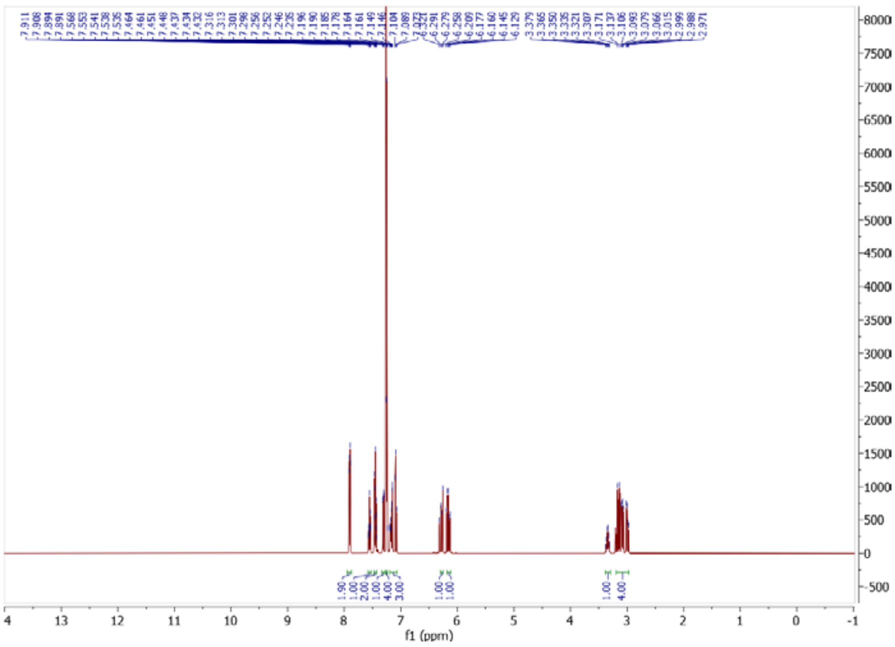

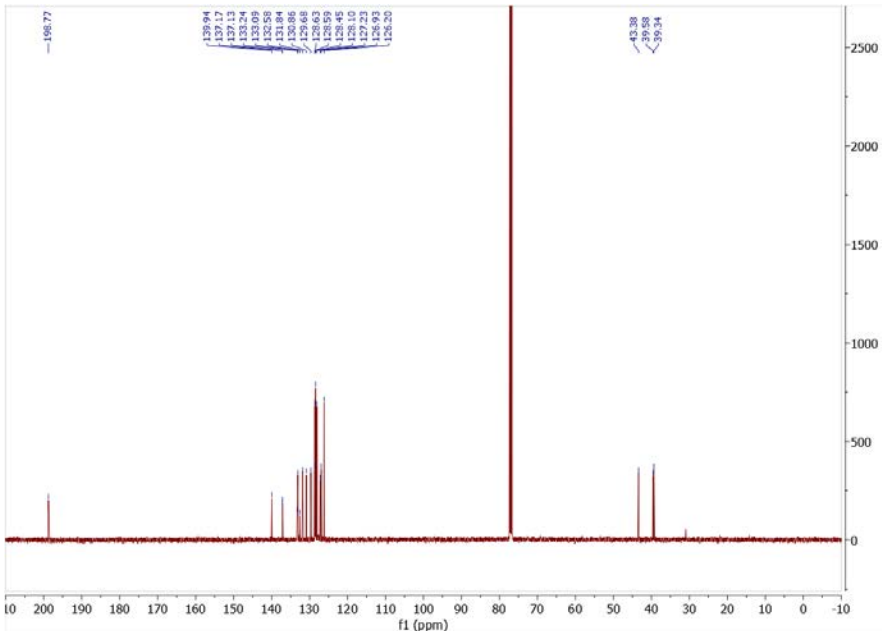

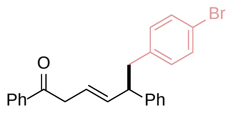

22

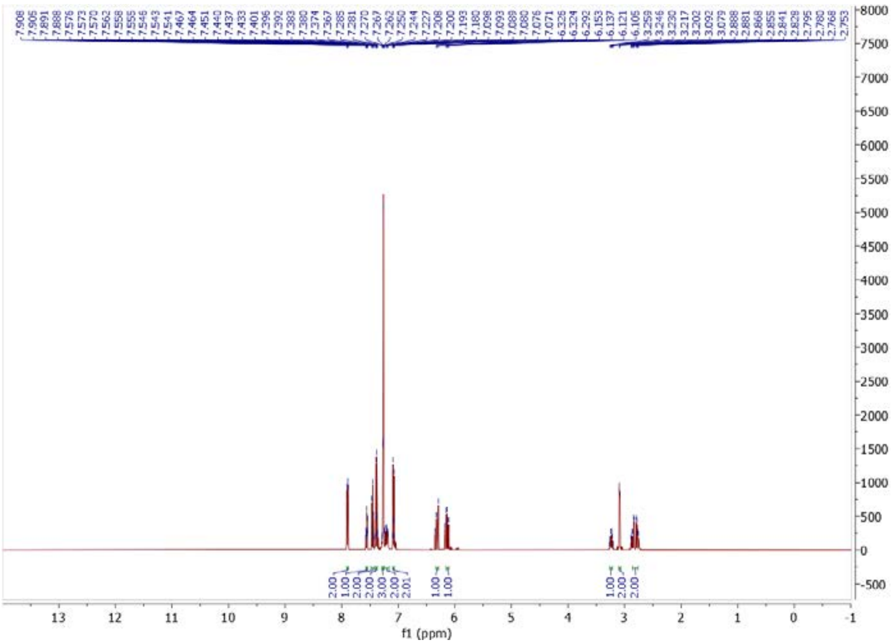

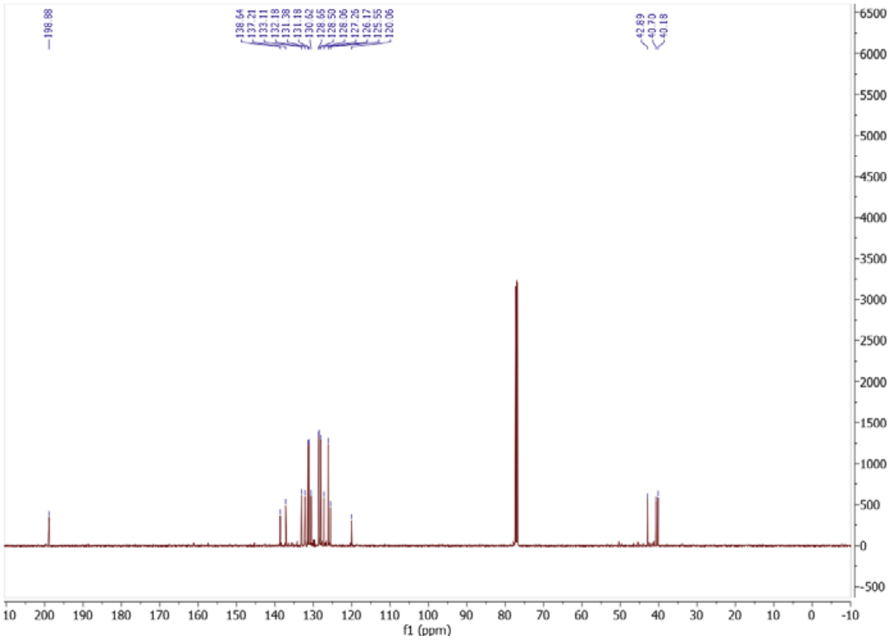

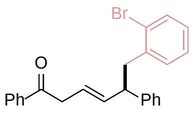

23

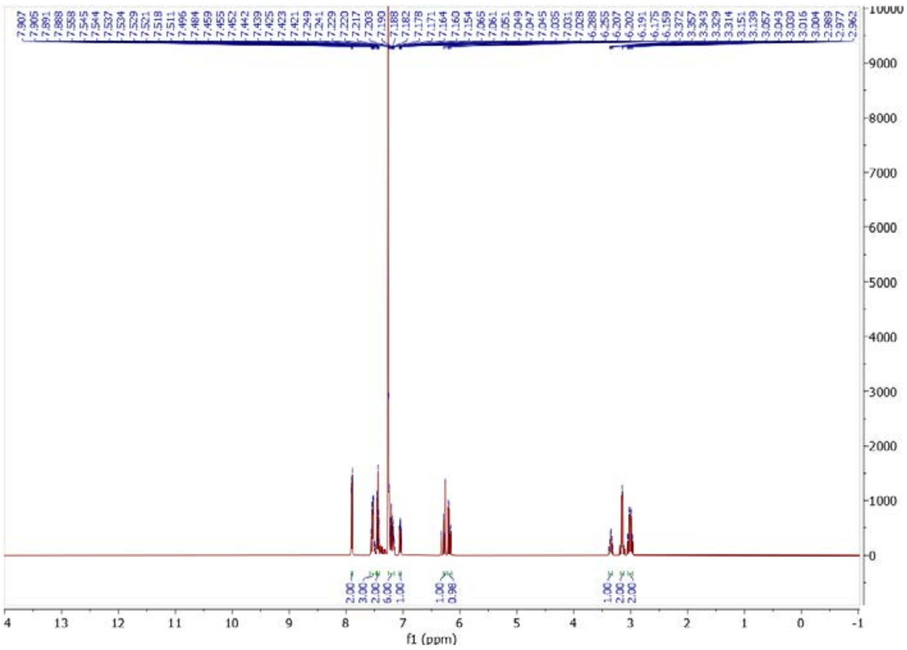

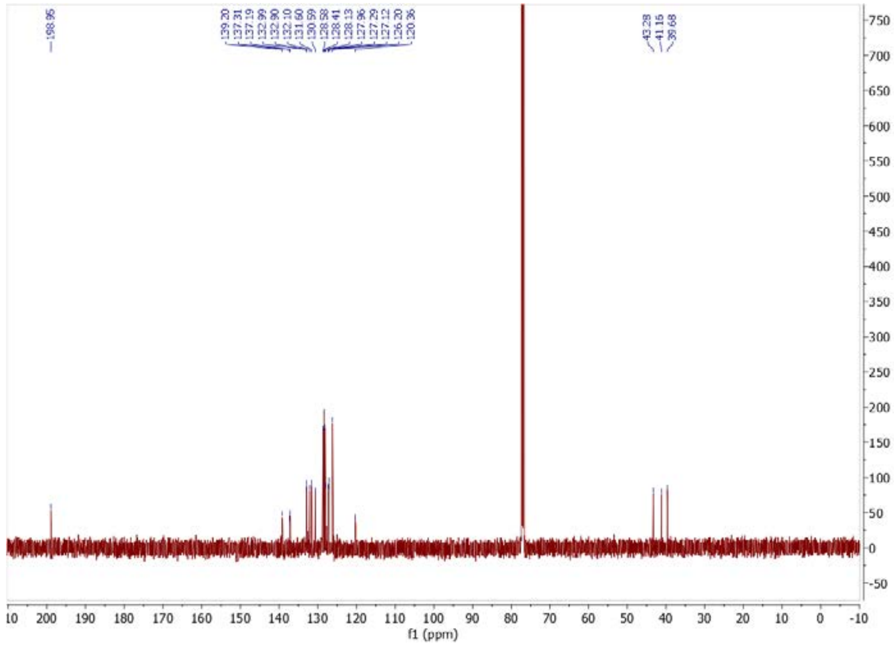

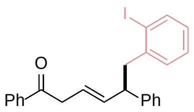

24

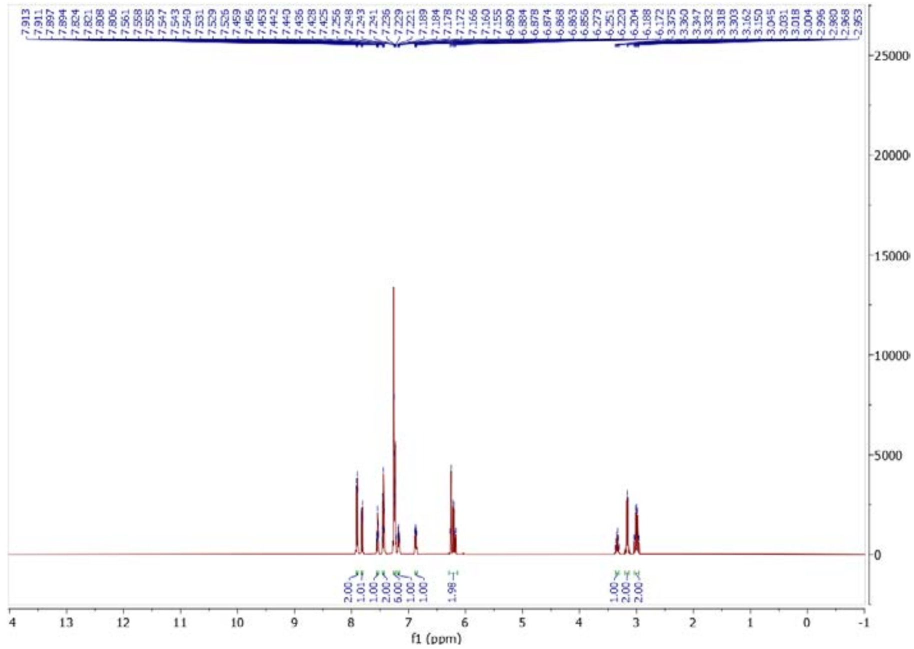

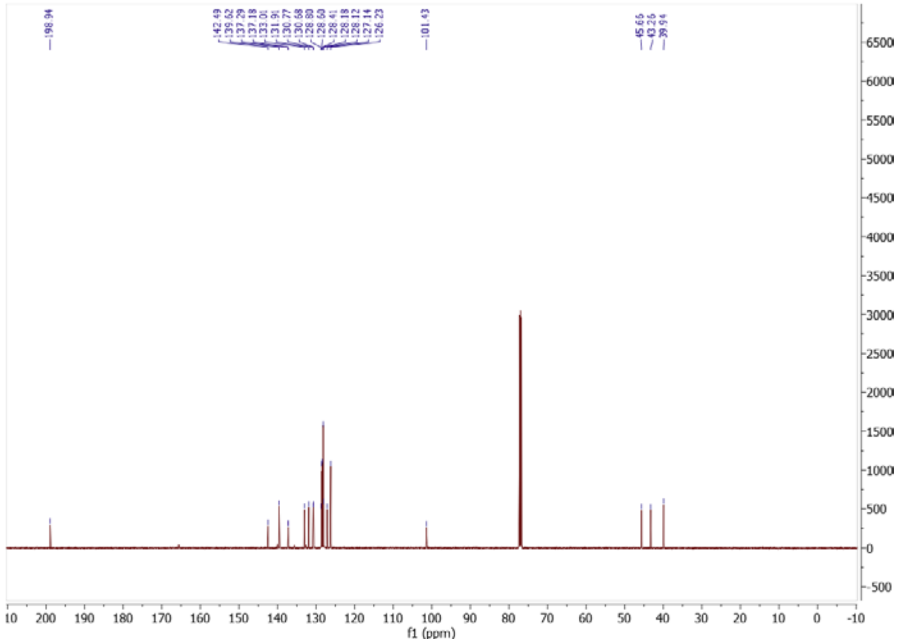

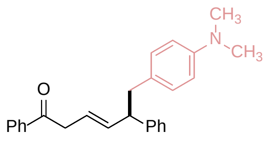

25

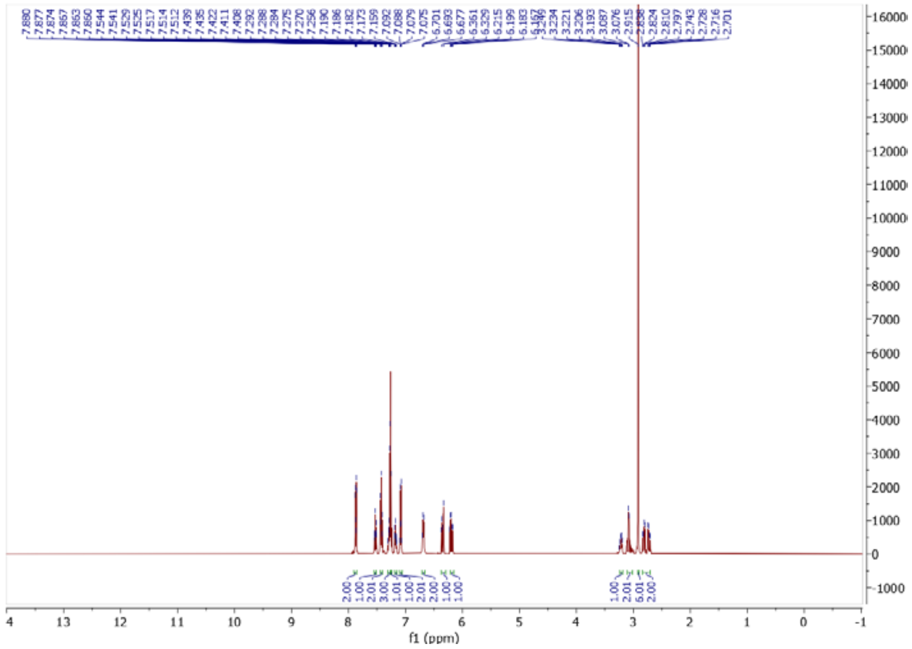

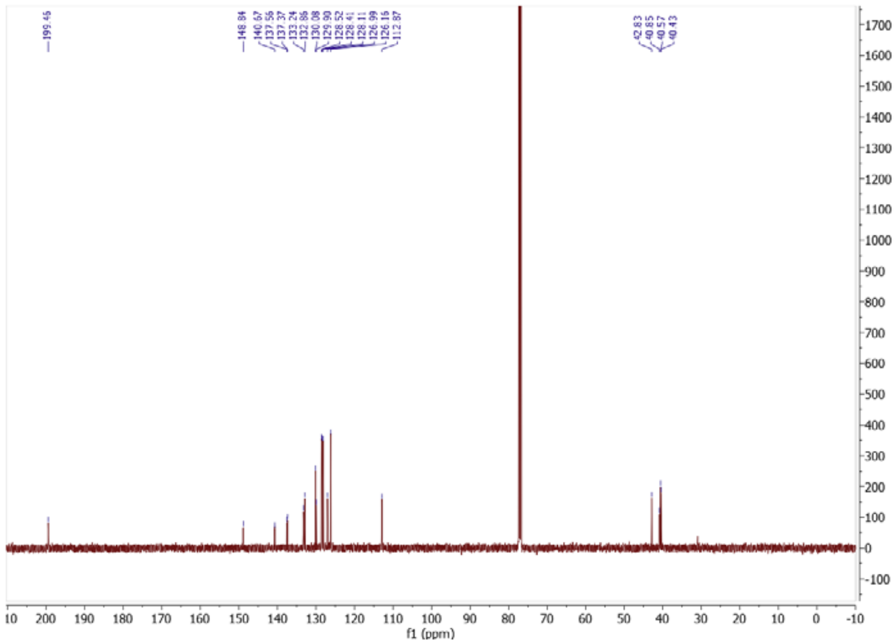

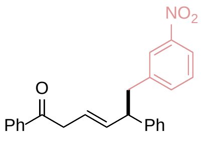

26

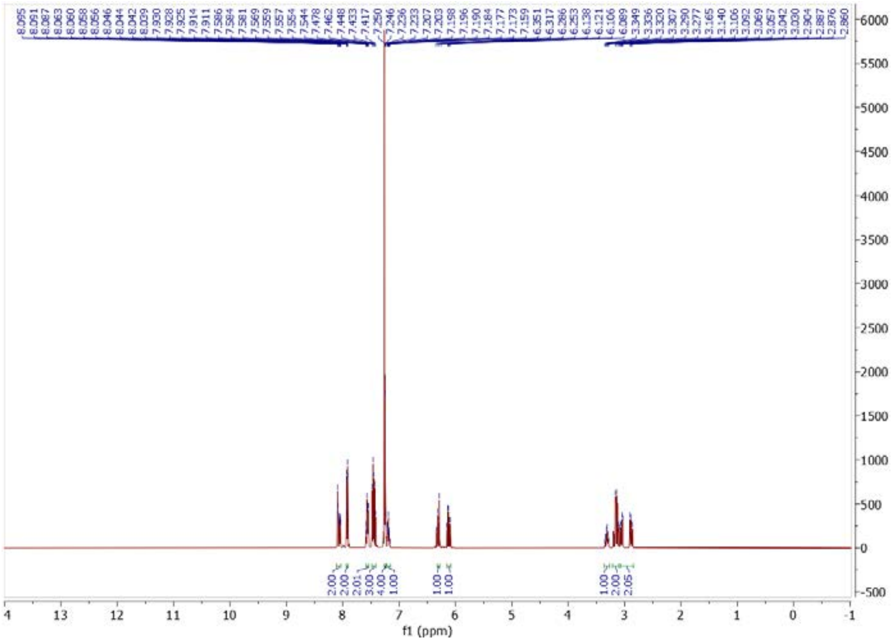

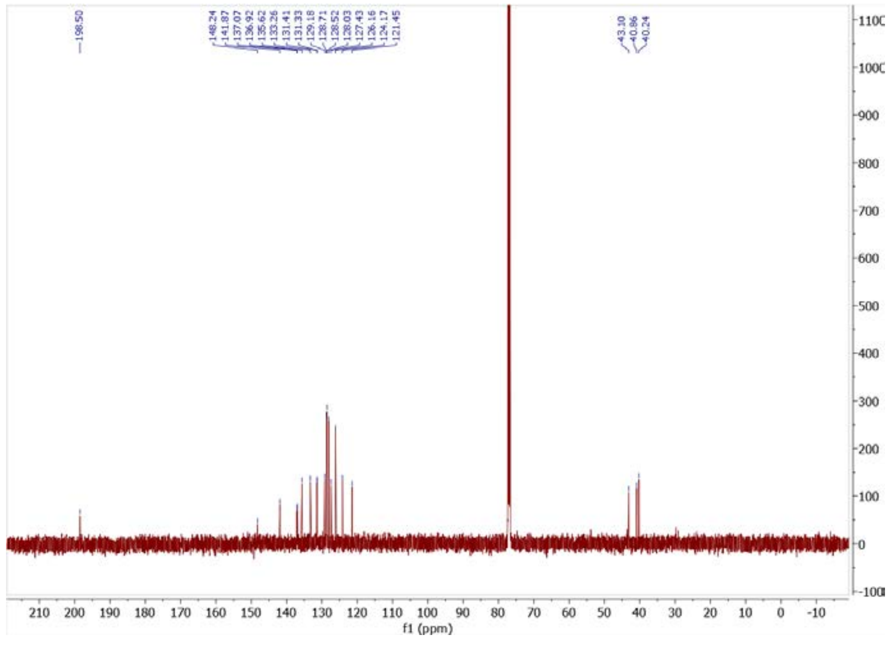

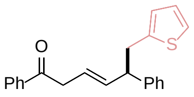

27

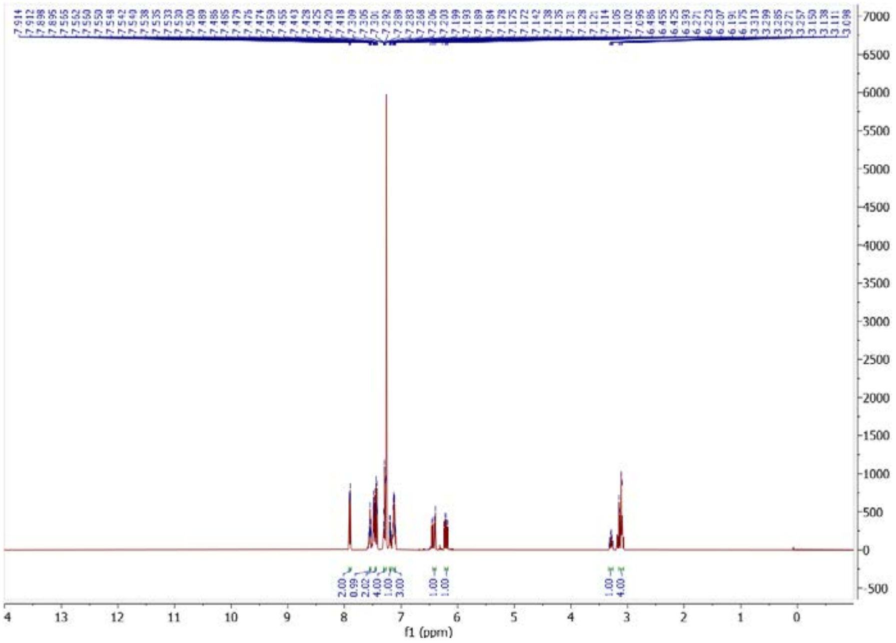

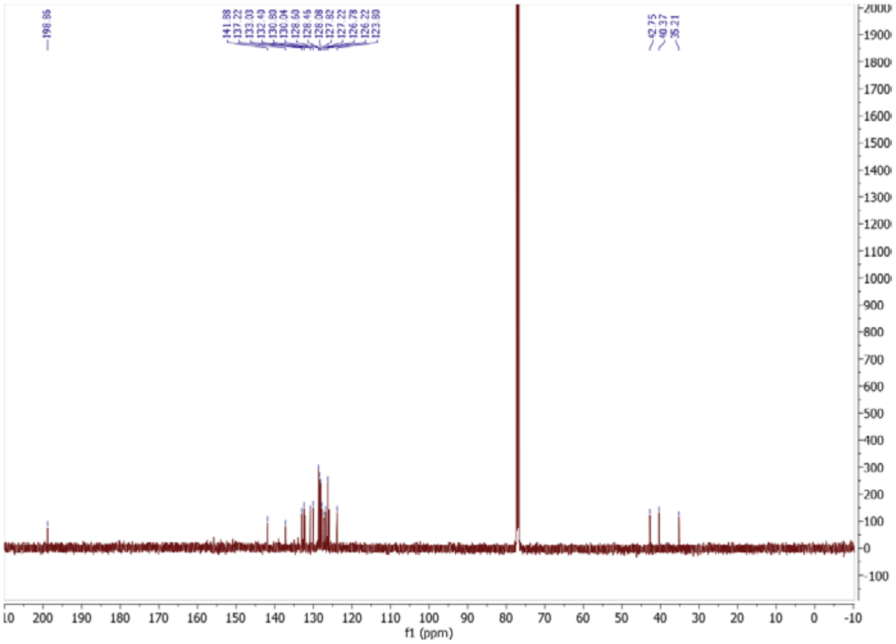

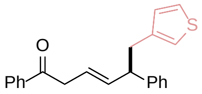

28

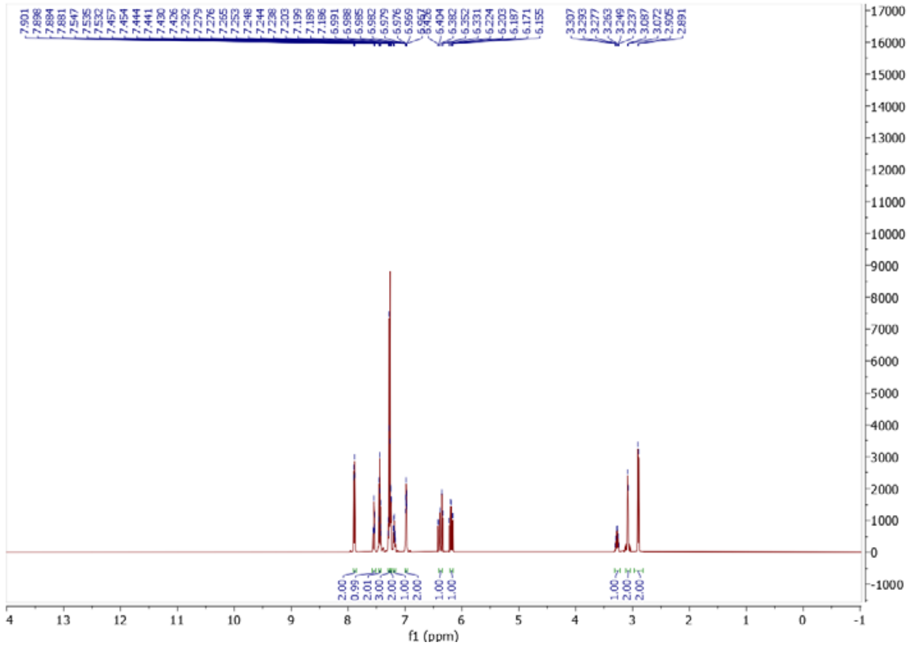

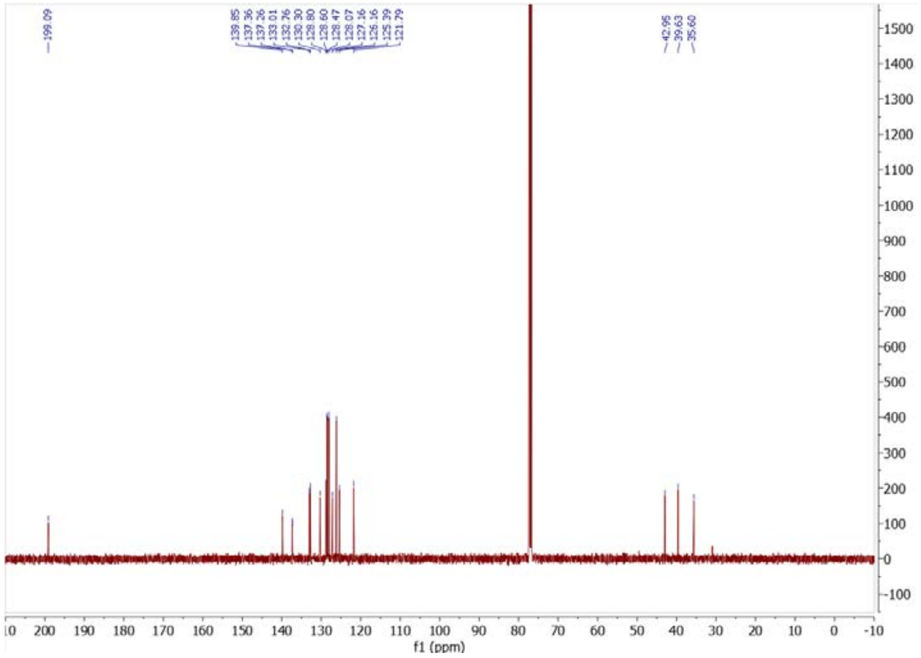

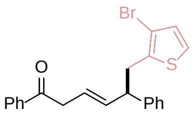

29

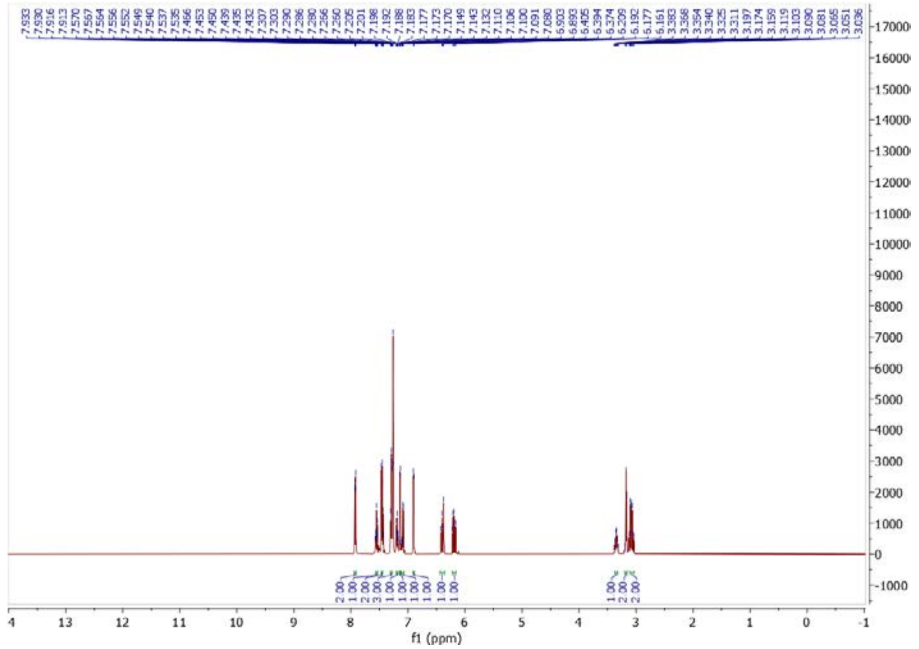

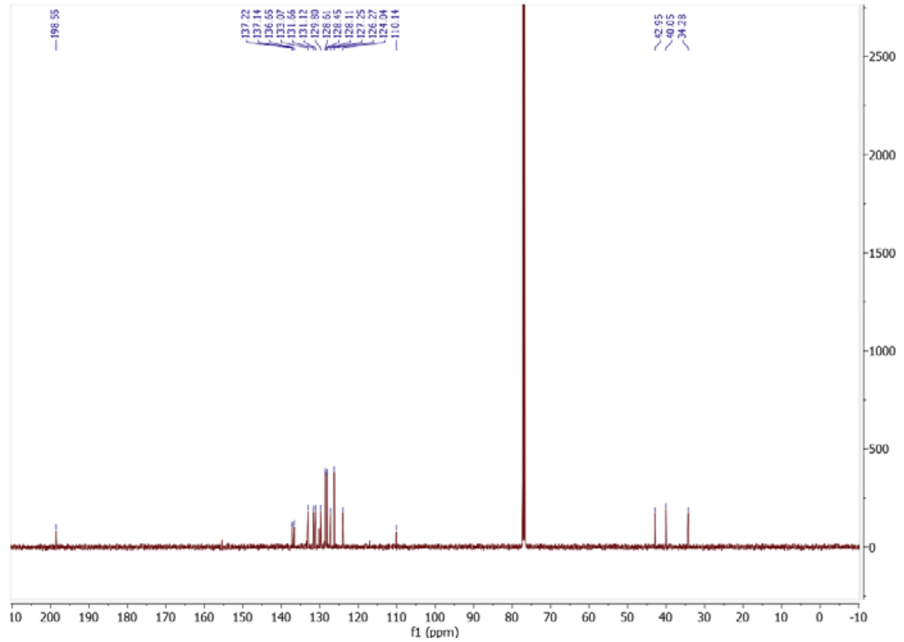

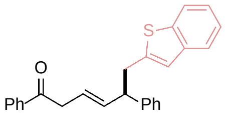

30

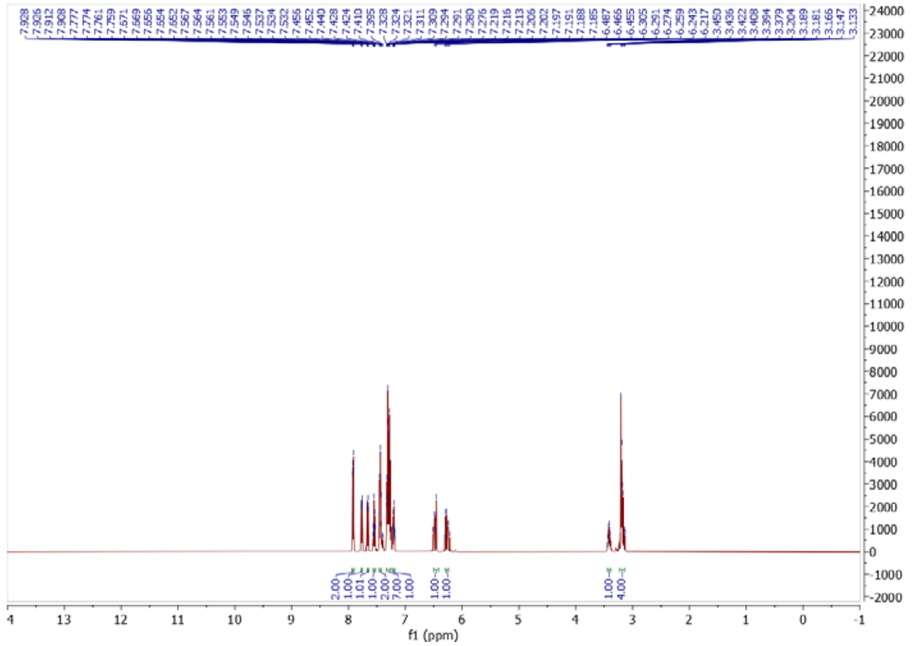

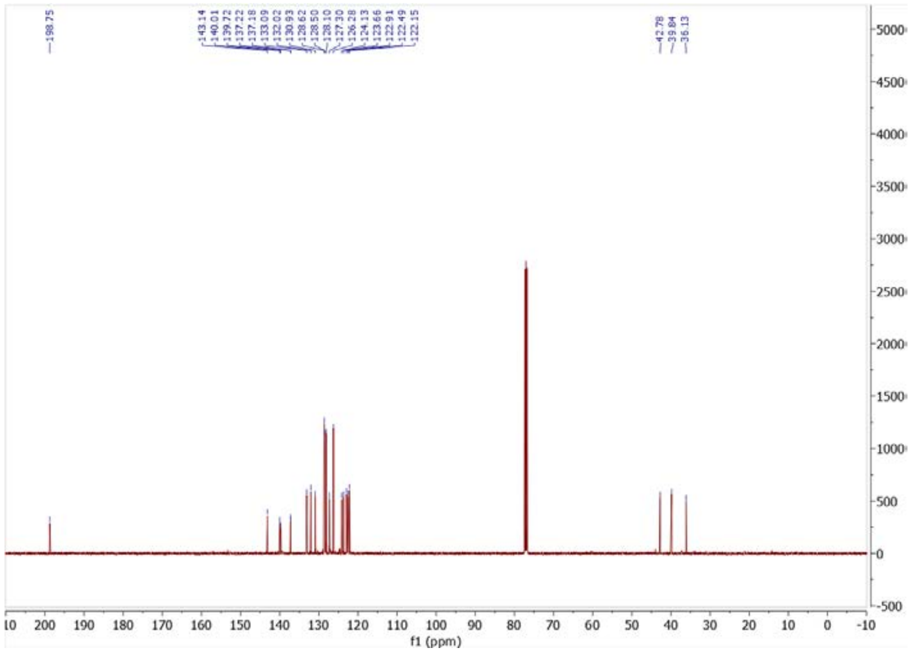

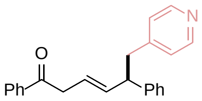

31

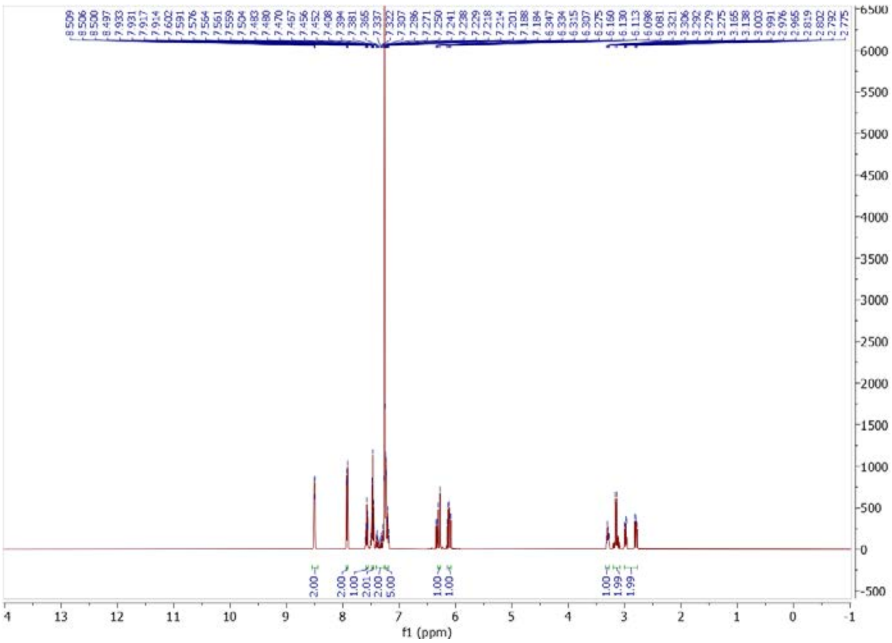

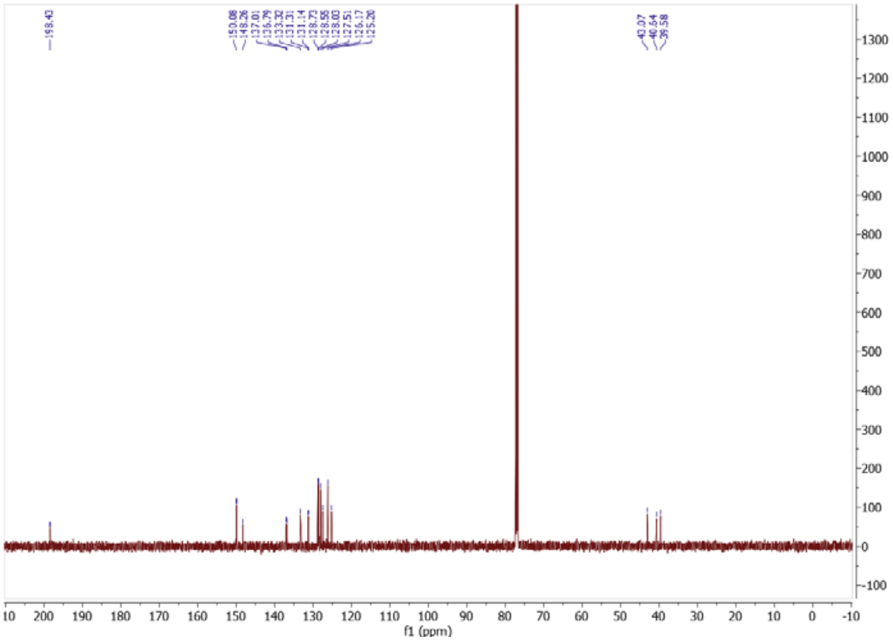

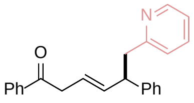

32

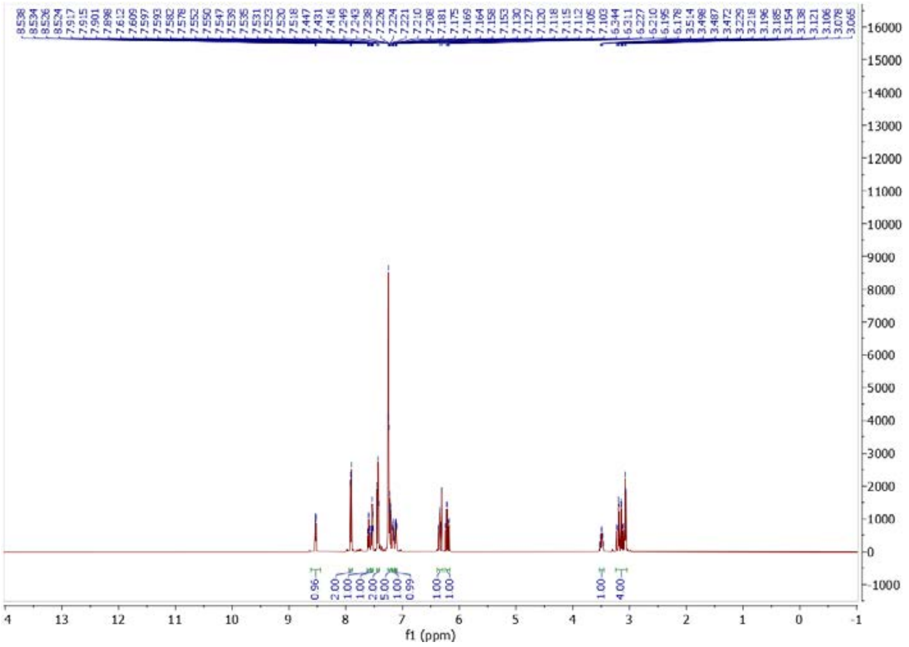

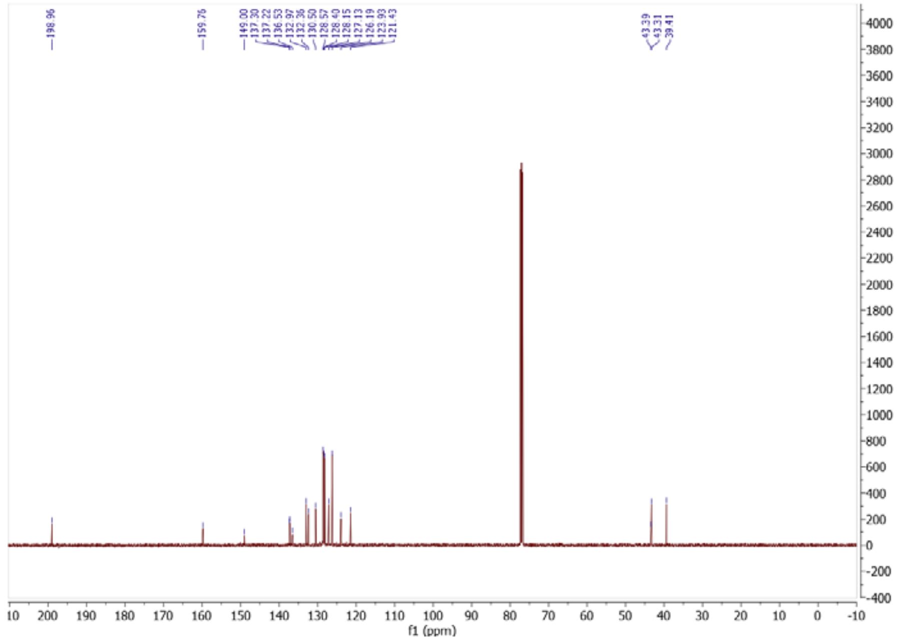

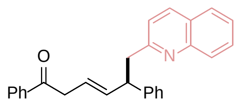

33

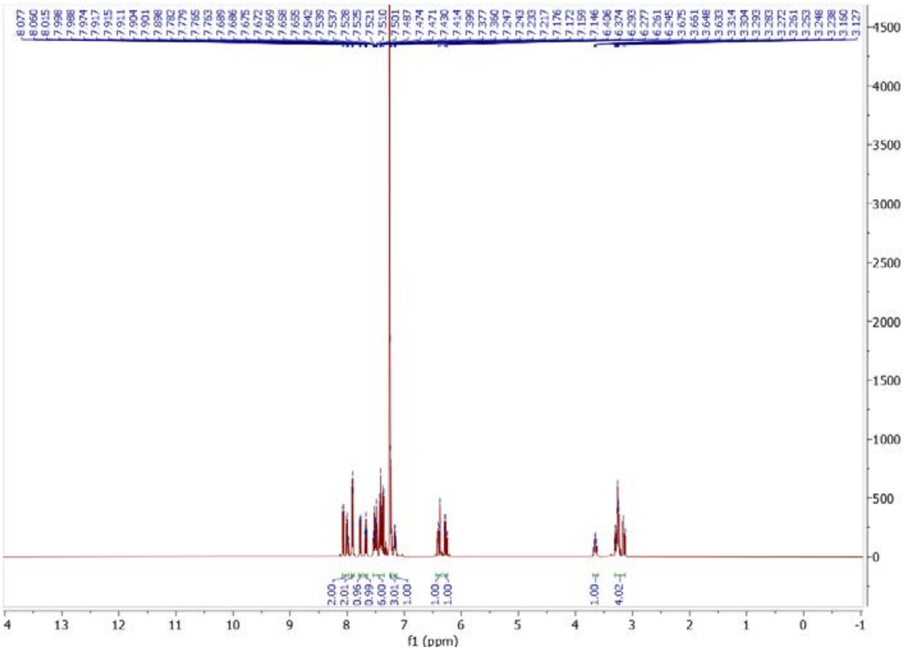

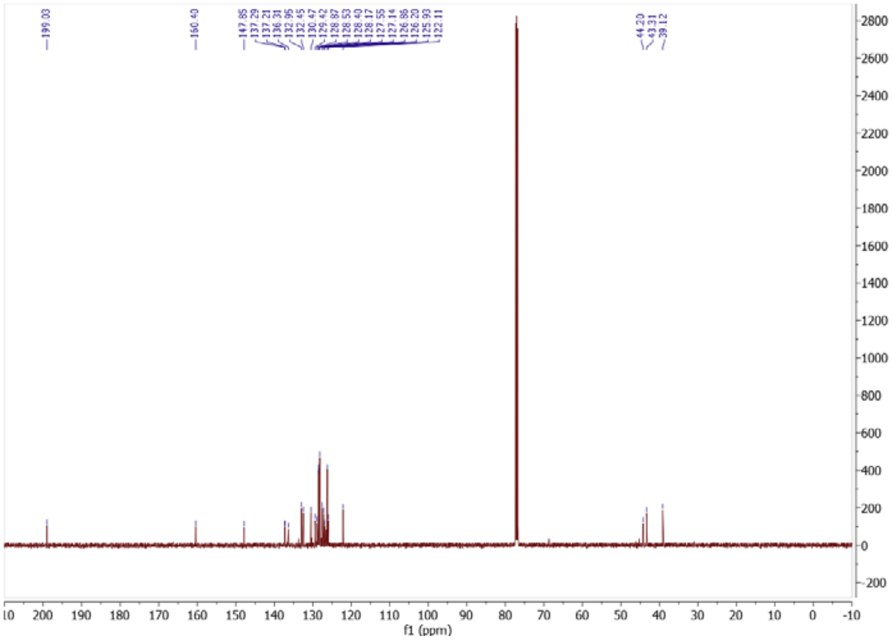

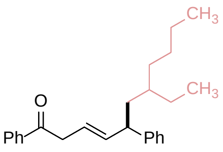

34

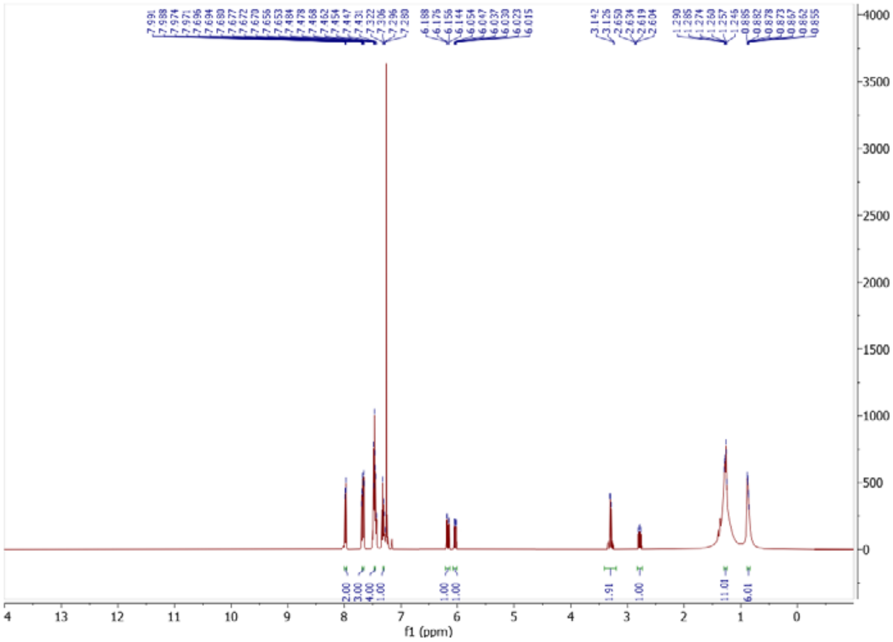

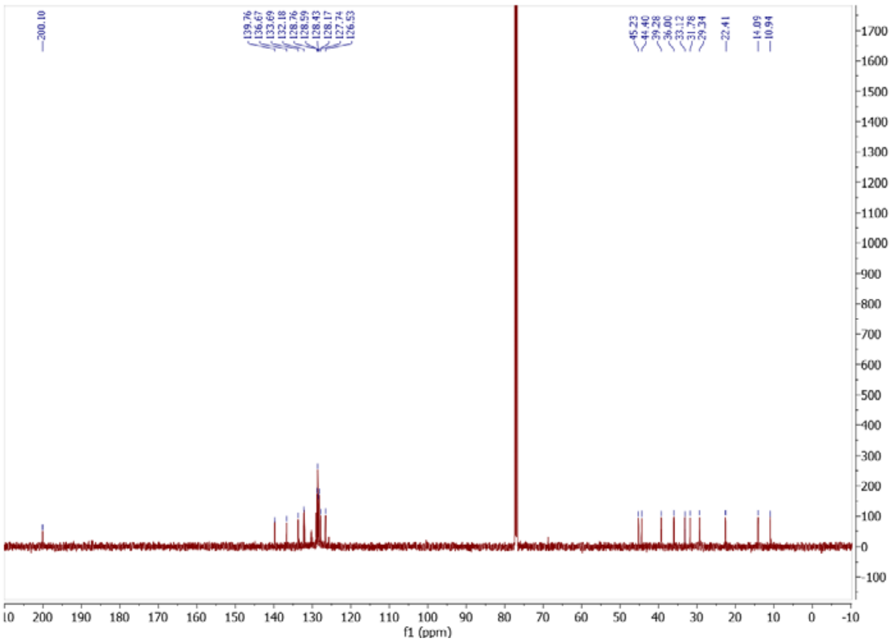

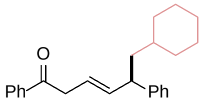
O
Ph
Ph

35

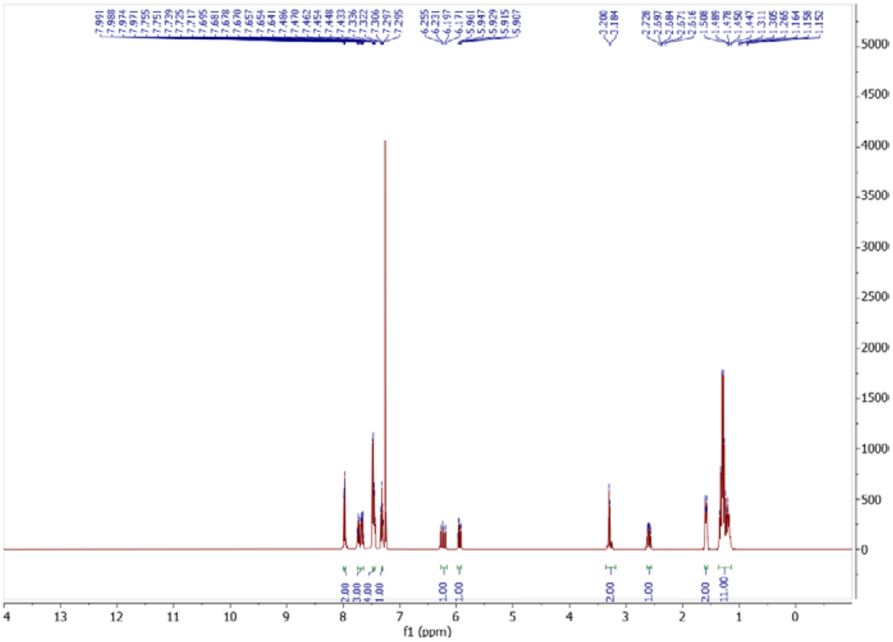
7.991
7.988
7.974
7.971
7.755
7.751
7.739
7.725
7.717
7.695
7.681
7.678
7.670
7.657
7.654
7.641
7.486
7.470
7.462
7.454
7.448
7.433
7.336
7.322
7.306
7.297
7.295
6.255
6.231
6.197
6.171
5.961
5.947
5.929
5.915
5.907
3.200
3.184
2.728
2.697
2.684
2.671
2.616
1.508
1.489
1.478
1.450
1.447
1.311
1.305
1.265
1.164
1.158
1.152
2.00
3.00
4.00
1.00
1.00
1.00
2.00
1.00
2.00
11.00
f1 (ppm)

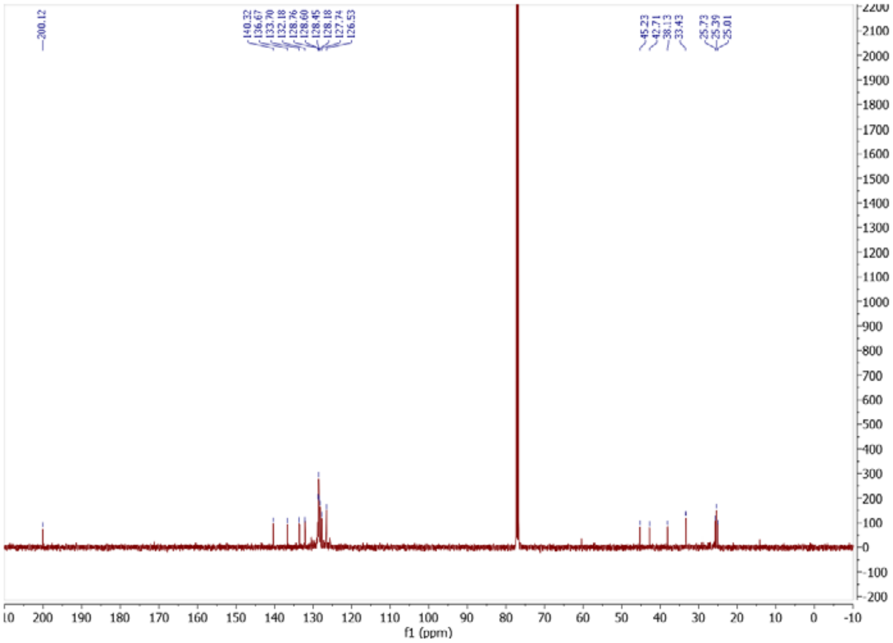
200.12
140.32
136.67
133.70
132.18
128.76
128.60
128.45
128.18
127.74
126.53
45.23
42.71
38.13
33.43
25.73
25.39
25.01
f1 (ppm)

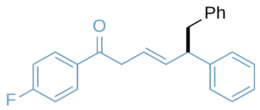

36

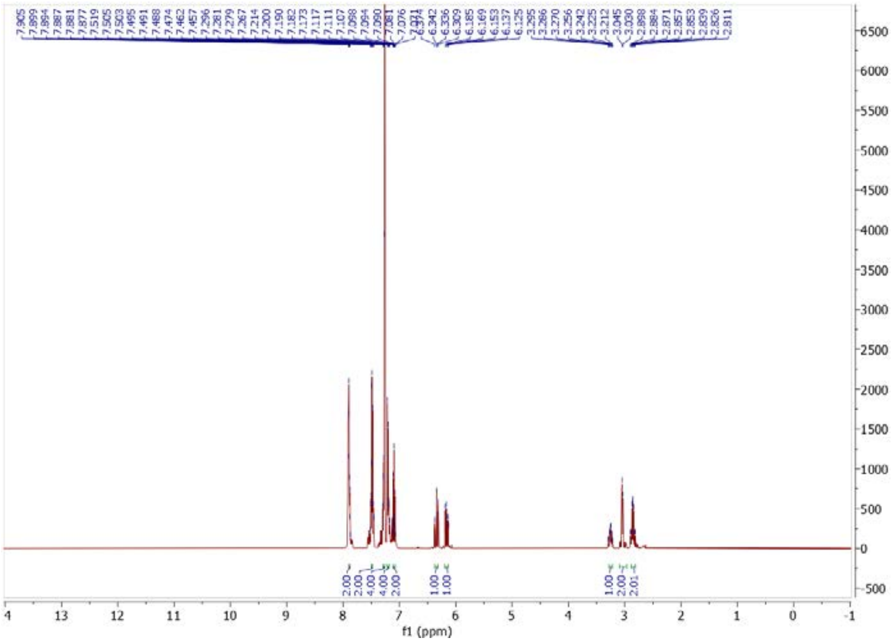

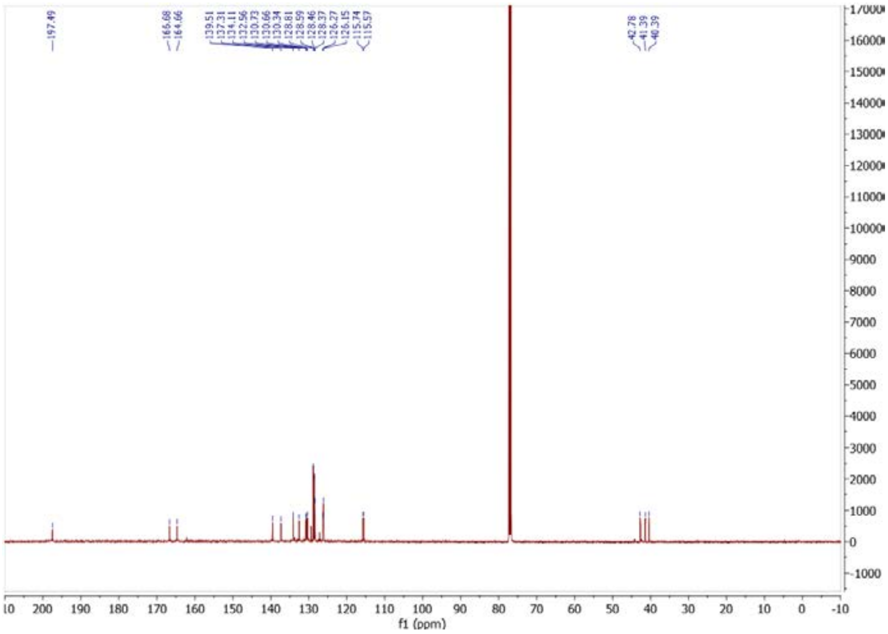

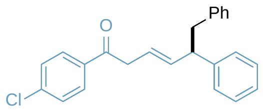

37

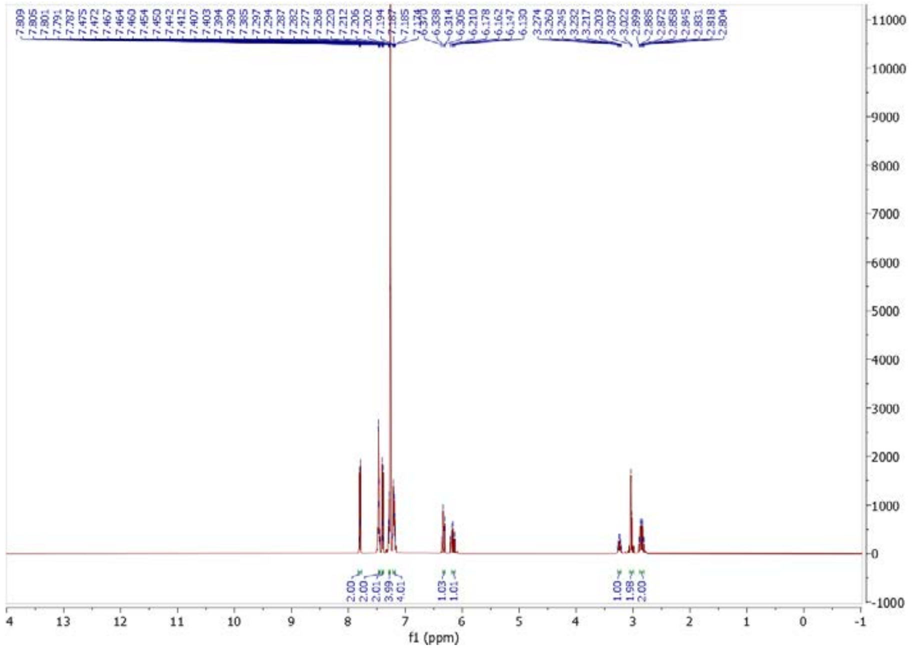

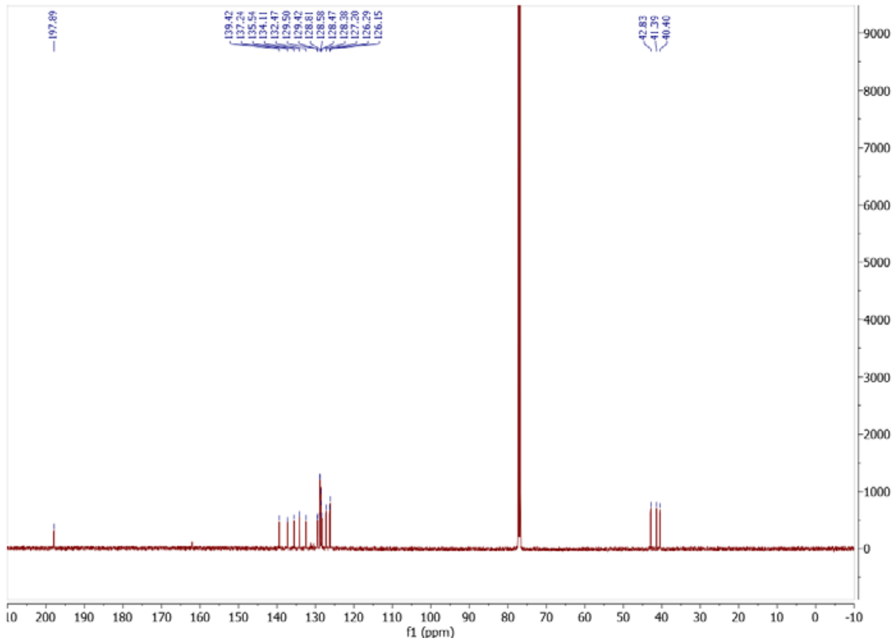

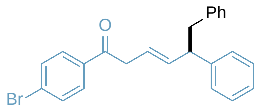

38

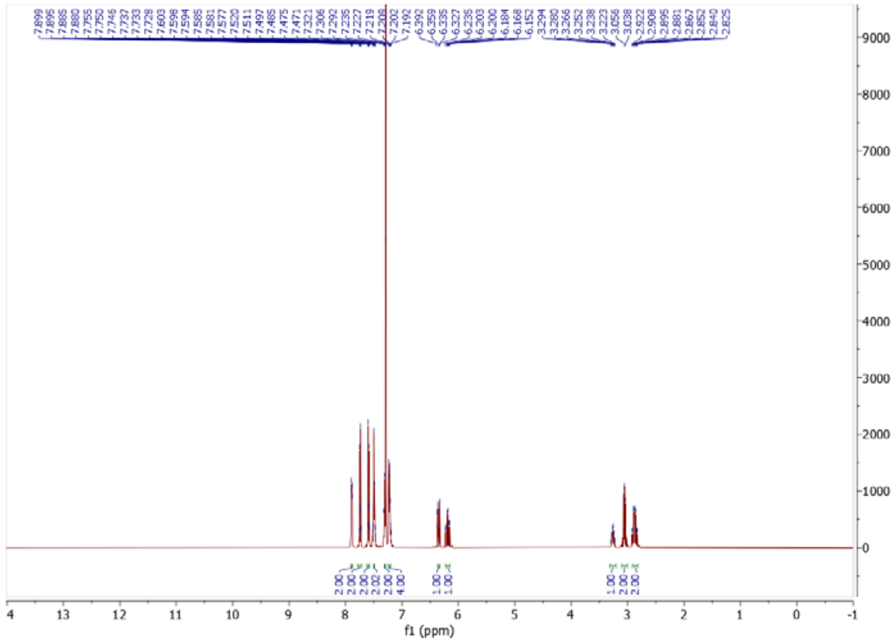

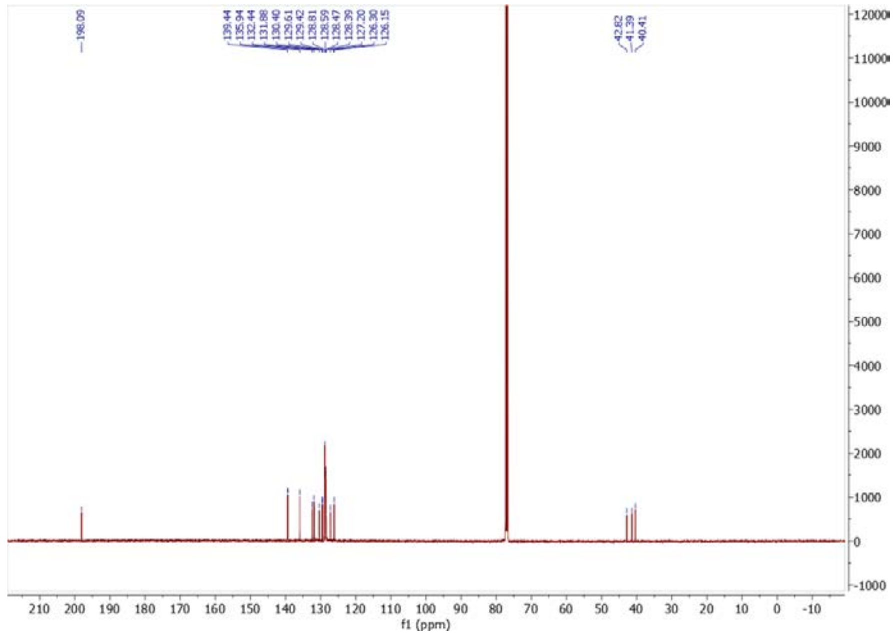

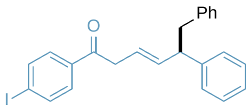

39

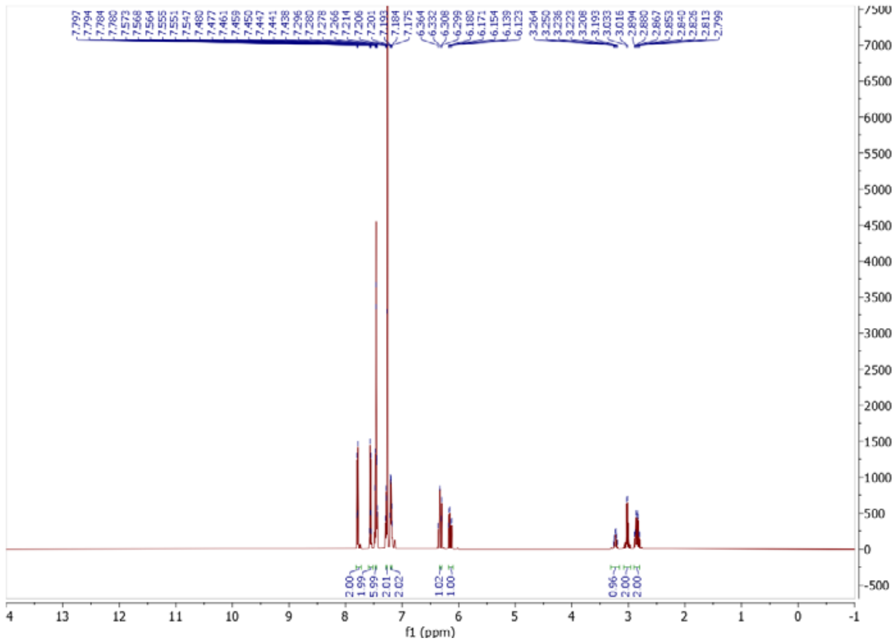

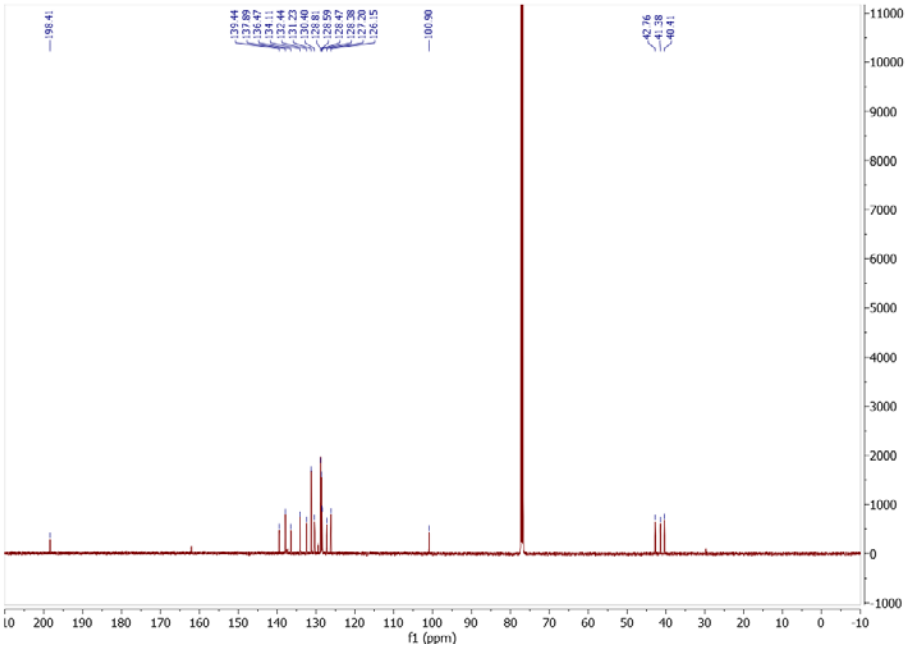

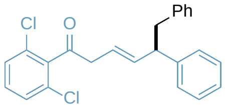

40

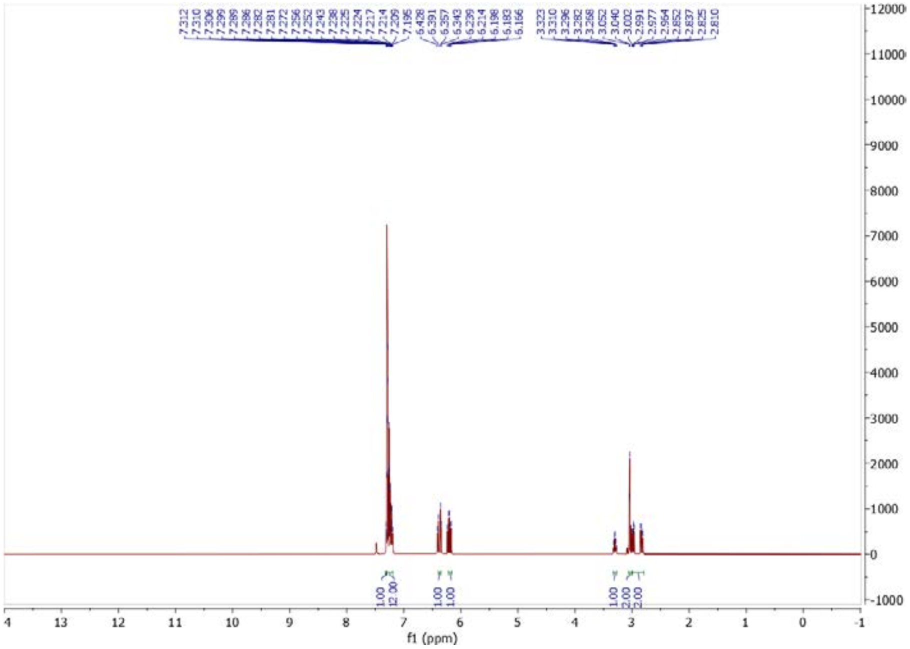

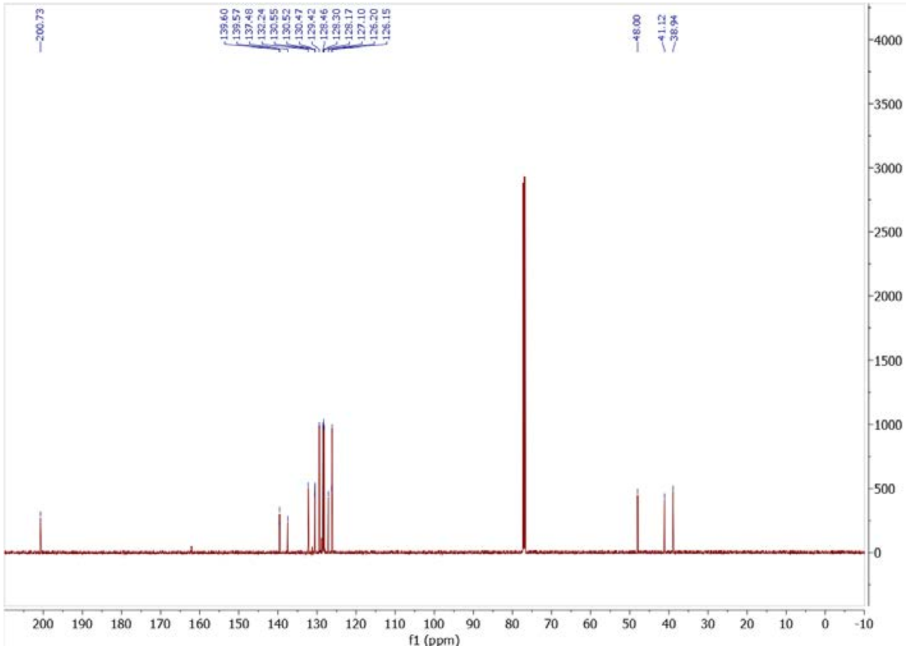

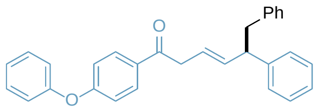

41

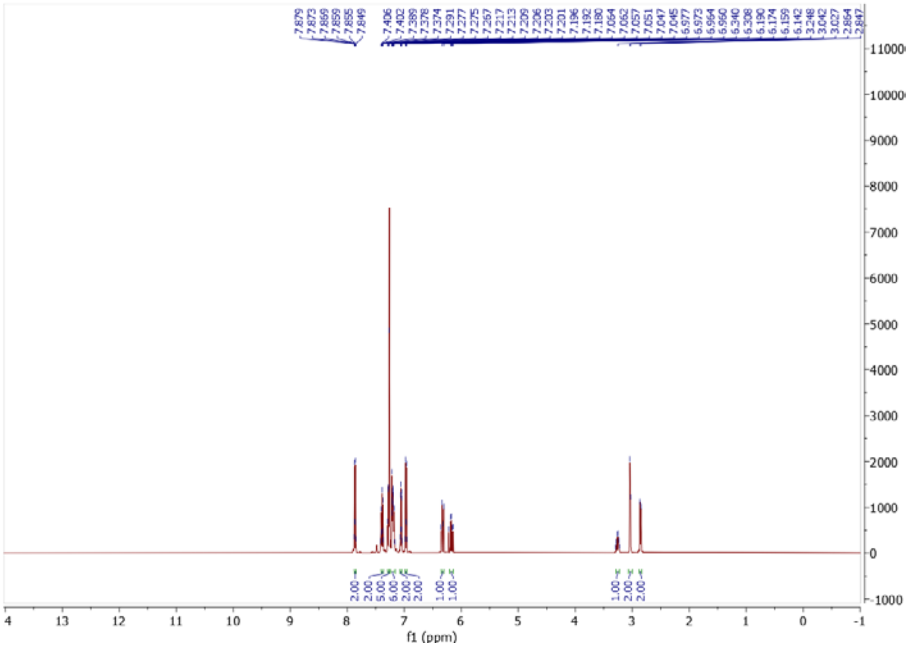

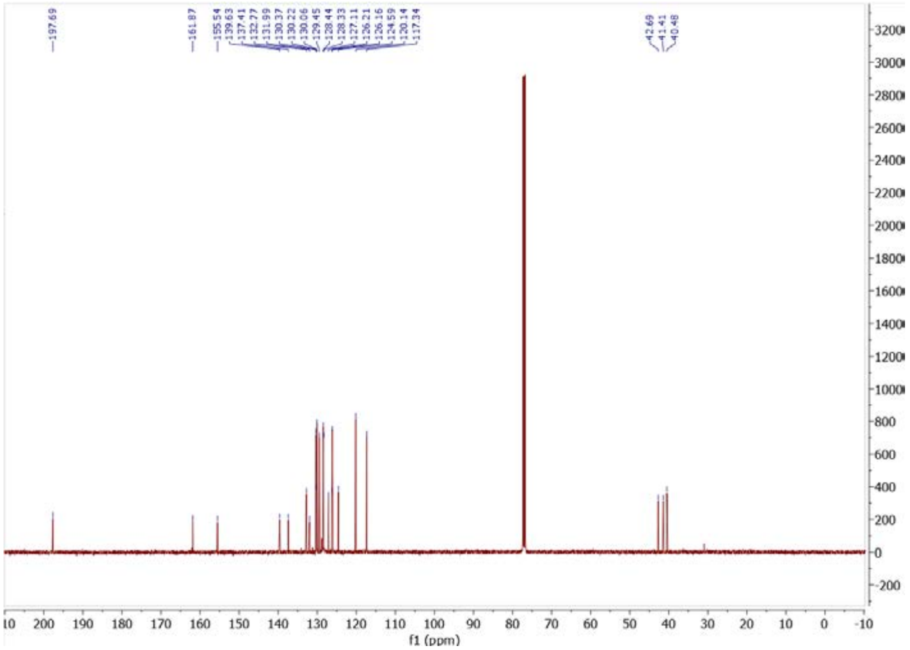

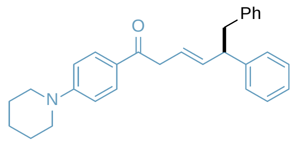

42

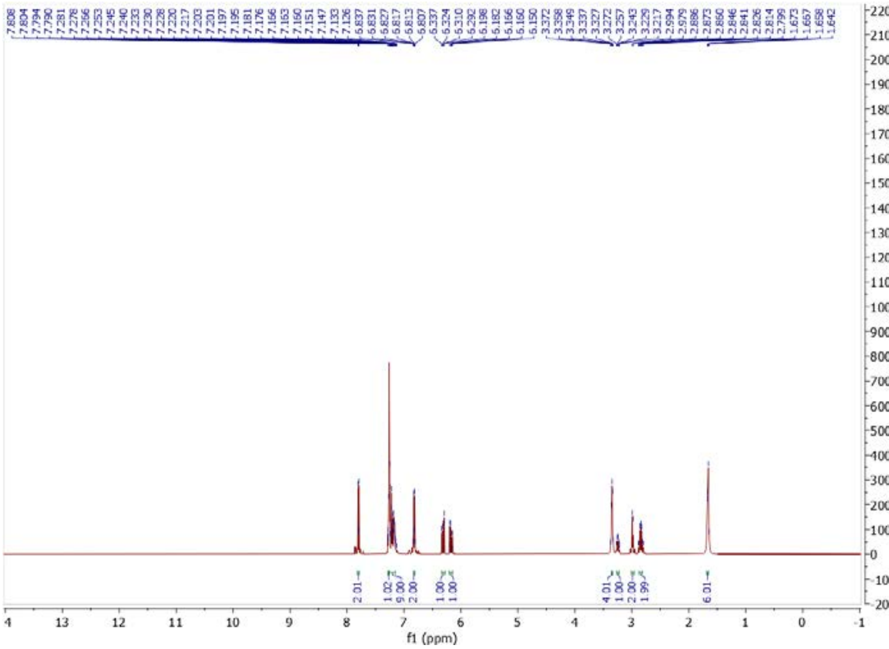

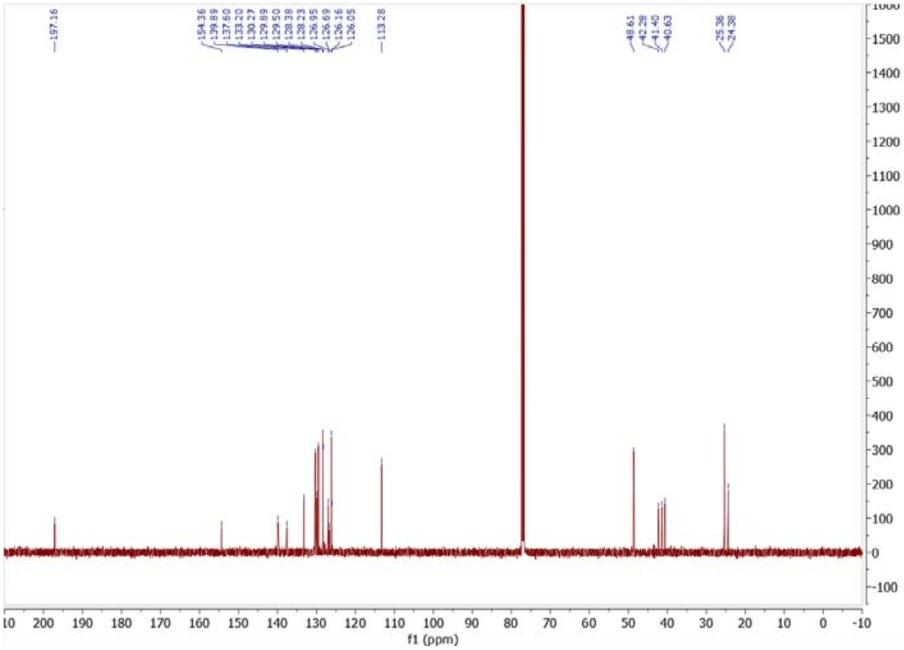

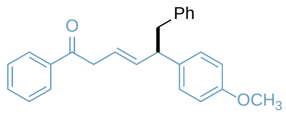

43

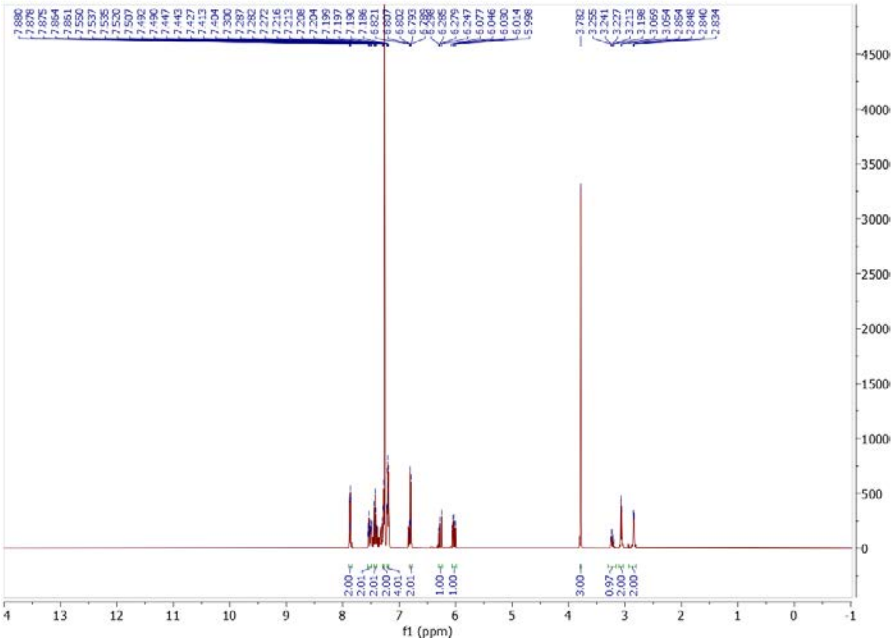

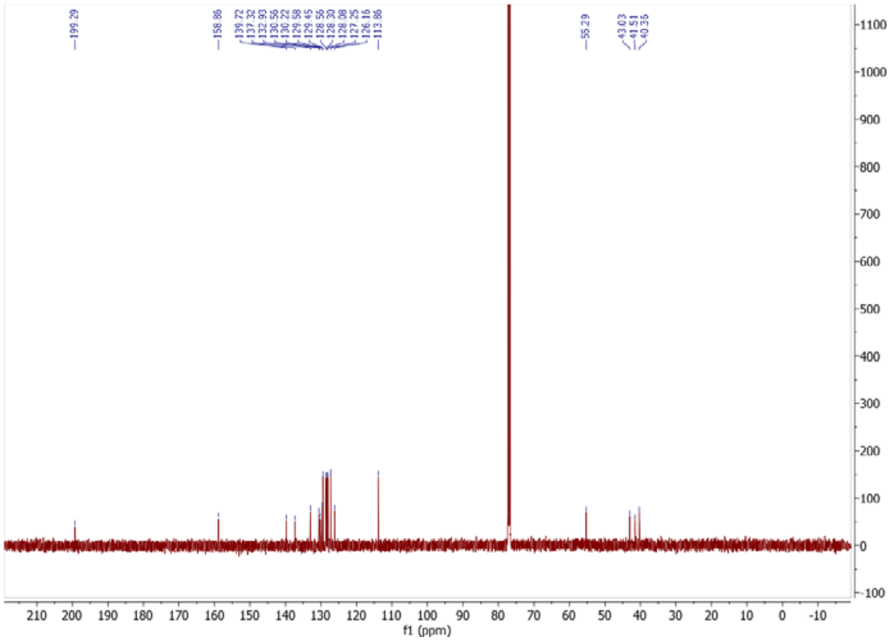

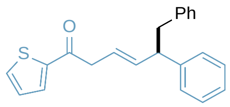
Ph
O
S

44

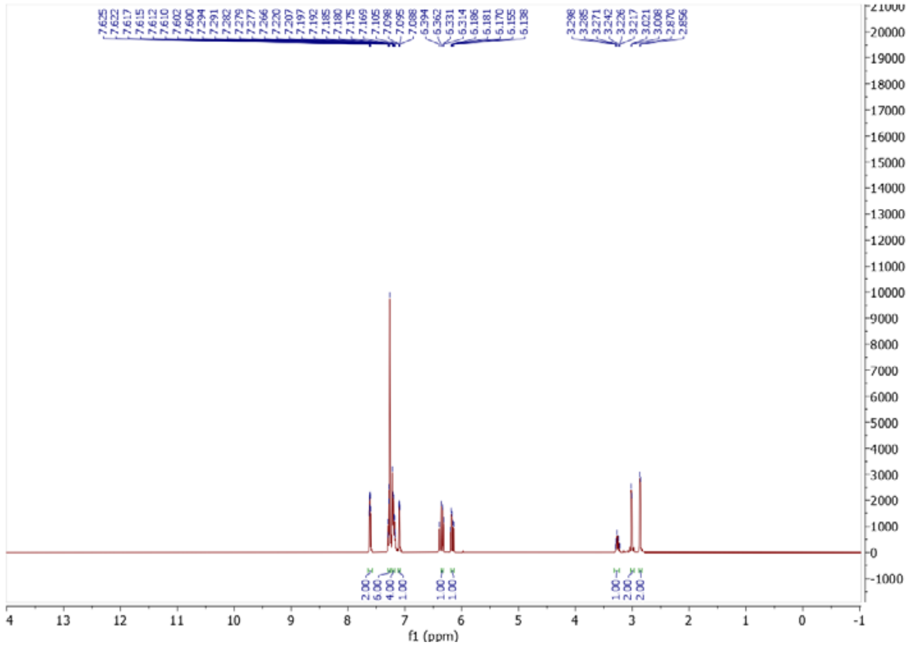
7.625 7.622 7.617 7.615 7.612 7.610 7.602 7.600 7.294 7.291 7.282 7.279 7.277 7.266 7.220 7.207 7.197 7.192 7.185 7.180 7.175 7.169 7.105 7.098 7.095 7.088 6.394 6.362 6.331 6.314 6.186 6.181 6.170 6.155 6.138
3.298 3.285 3.271 3.242 3.226 3.217 3.021 3.008 2.870 2.856
2.00 6.00 4.00 1.00 1.00 1.00 1.00 2.00 2.00
f1 (ppm)

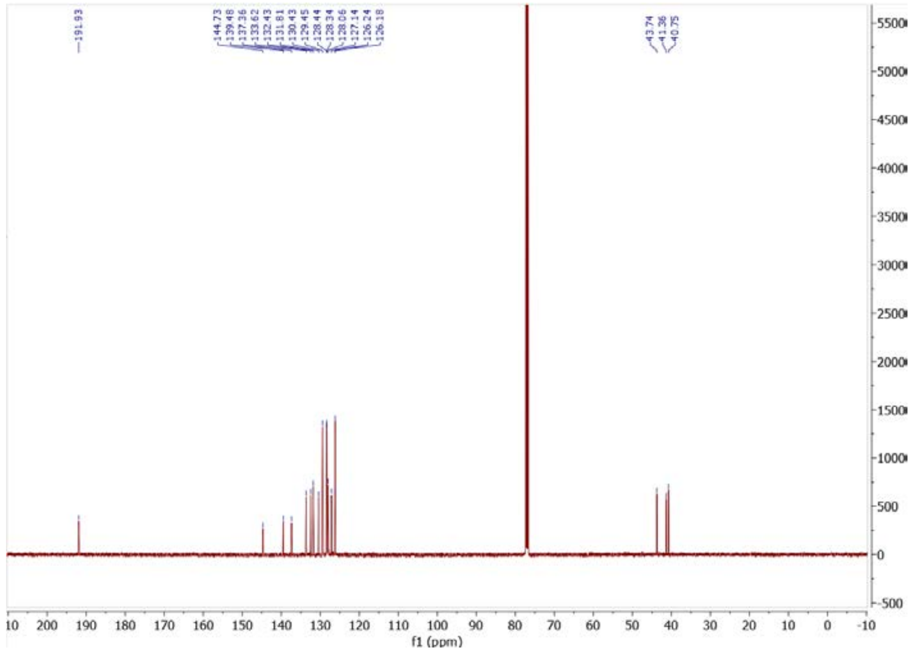
191.93
144.73 139.48 137.36 133.62 132.43 131.81 130.43 129.45 128.44 128.34 128.06 127.14 126.24 126.18
43.74 41.36 40.75
f1 (ppm)

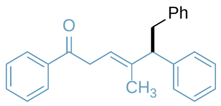

45

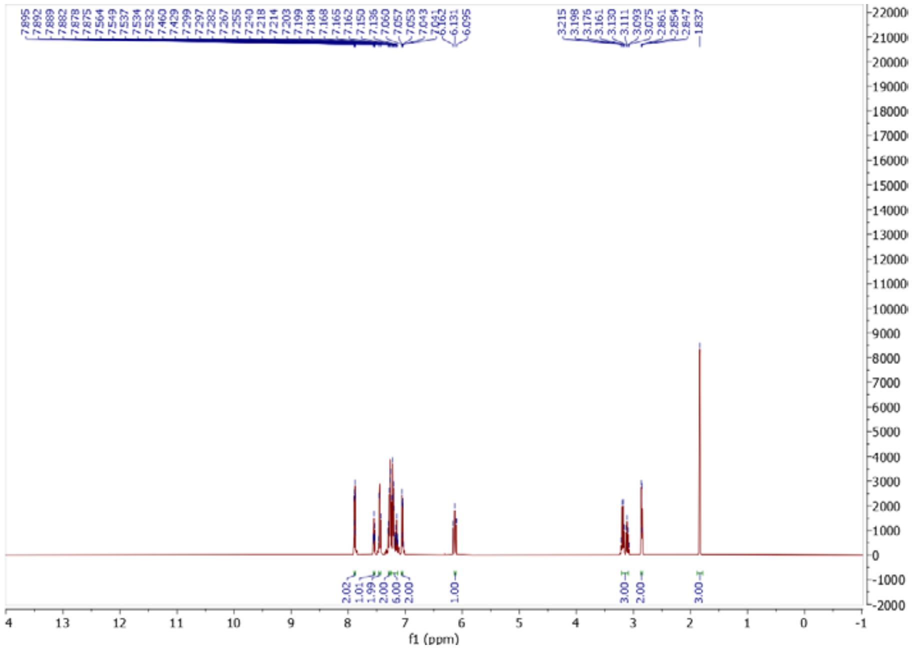

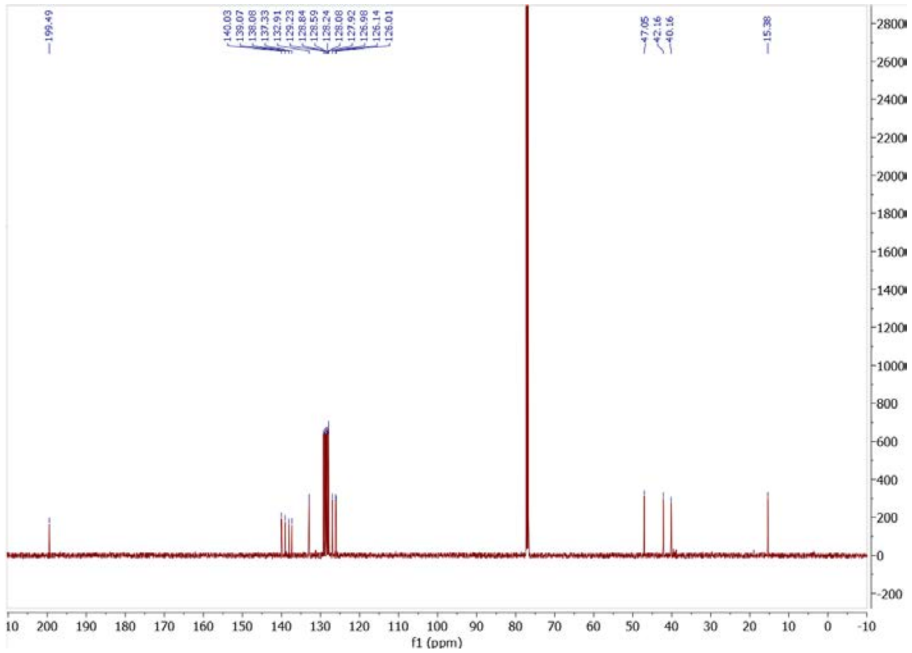

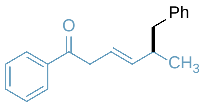

O
Ph
CH3

46

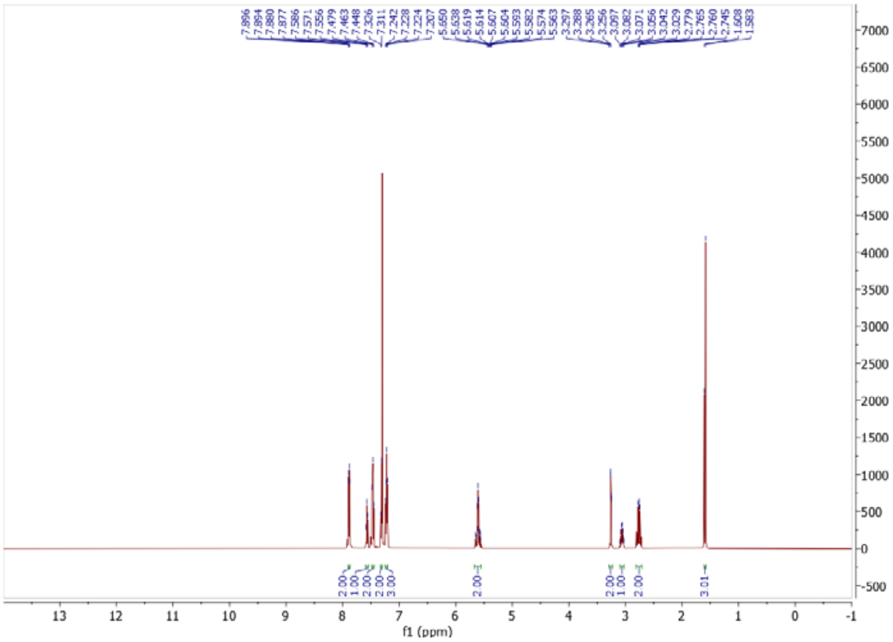

7.896
7.894
7.880
7.877
7.586
7.571
7.556
7.479
7.463
7.448
7.326
7.311
7.242
7.228
7.224
7.207
5.650
5.638
5.619
5.614
5.607
5.604
5.593
5.582
5.574
5.563
3.297
3.288
3.265
3.256
3.097
3.082
3.071
3.056
3.042
3.029
2.779
2.765
2.760
2.745
1.603
1.583
2.00
1.00
2.00
2.00
3.00
2.00
2.00
1.00
2.00
3.01
f1 (ppm)

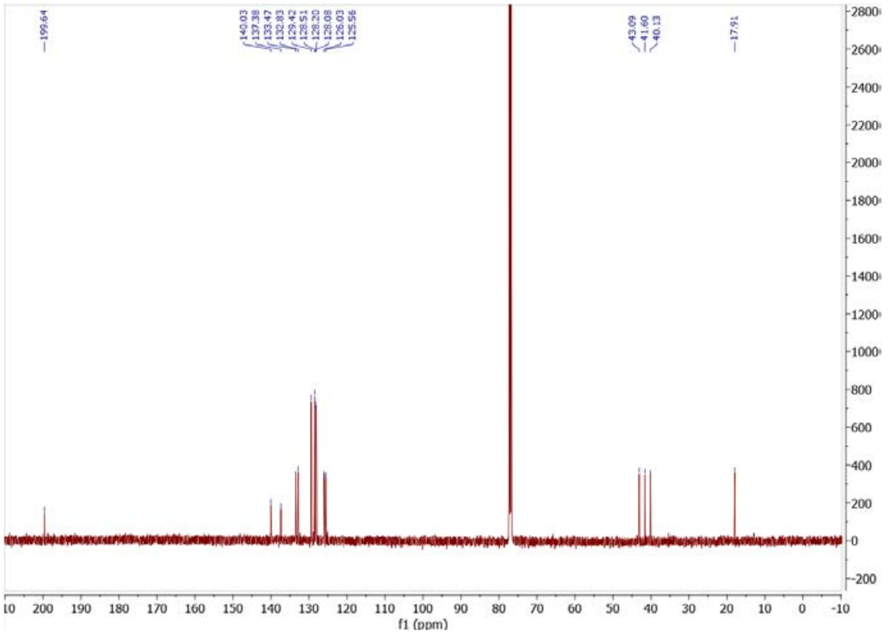

199.64
140.03
137.38
133.47
132.83
129.42
128.51
128.20
128.08
126.03
125.56
43.09
41.60
40.13
17.91
f1 (ppm)

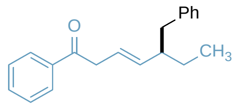
Ph
O
CH3

47

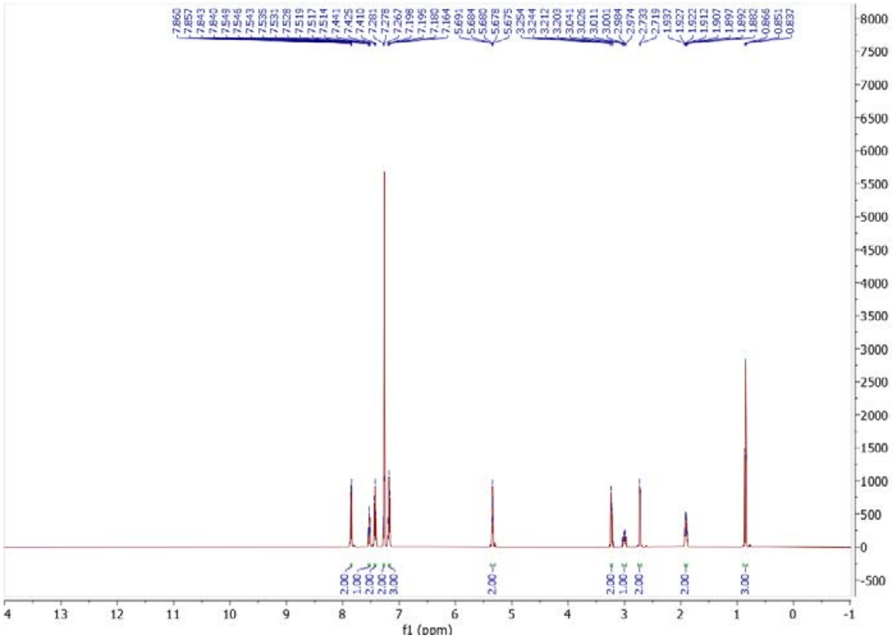
7.860 7.857 7.843 7.840 7.549 7.546 7.543 7.535 7.531 7.528 7.519 7.517 7.514 7.441 7.425 7.410 7.281 7.278 7.267 7.198 7.195 7.180 7.164 5.691 5.684 5.680 5.678 5.675 3.254 3.244 3.212 3.203 3.041 3.026 3.011 3.001 2.984 2.974 2.733 2.719 1.937 1.927 1.922 1.912 1.907 1.897 1.892 1.882 0.866 0.851 0.837
2.00 1.00 2.00 2.00 3.00 2.00 2.00 1.00 2.00 2.00 3.00
f1 (ppm)

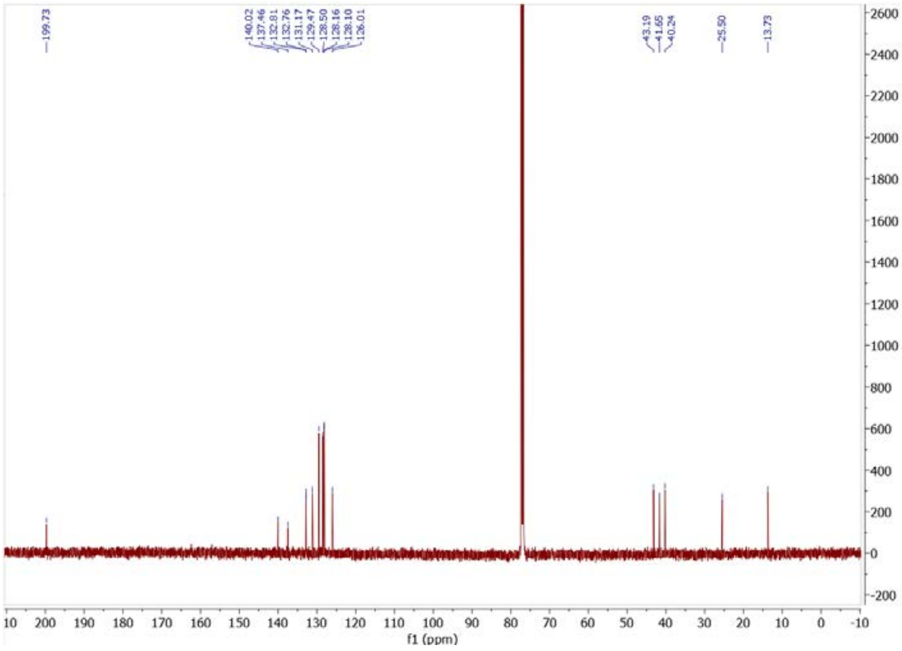
199.73
140.02 137.46 132.81 132.76 131.17 129.47 128.50 128.16 128.10 126.01
43.19 41.65 40.24
25.50
13.73
f1 (ppm)

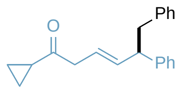

48

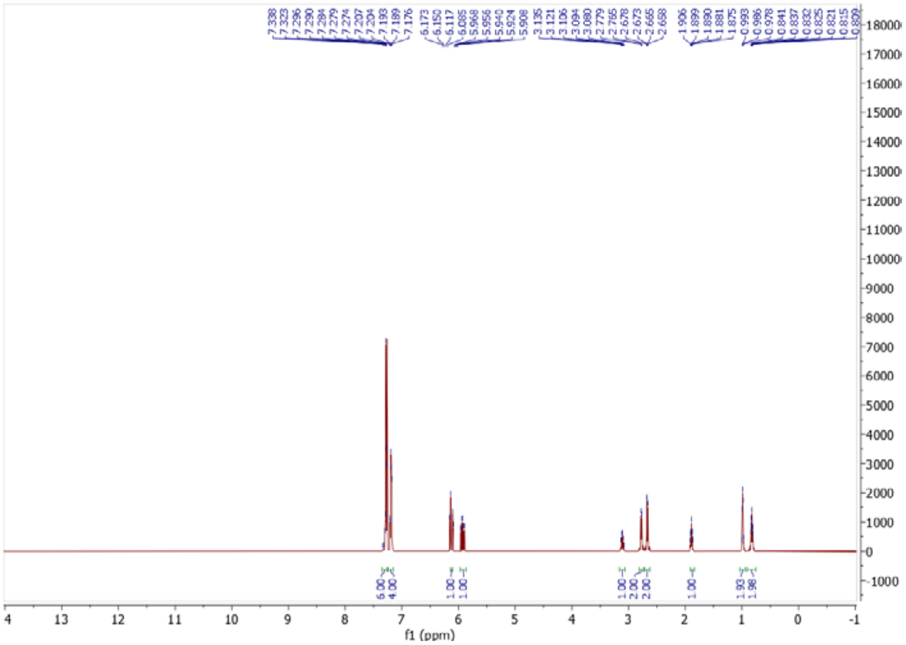

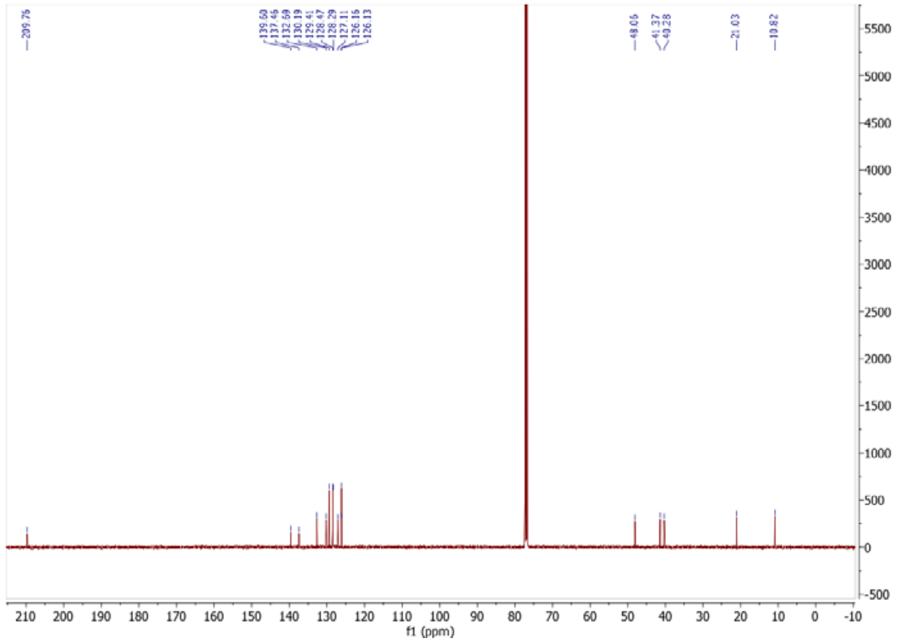

10. HPLC Chromatograph

HPLC instrument: Agilent Technologies 1260 Infinity instrument equipped with a quaternary pump

Column: Chiralpak OD-H, Daicel Corporation (250 mmL × 4.6 mm, 5μm)

Eluent: Pentane/IPA (98:2)

Flow rate: 0.7 mL/min

λ = 260 nm

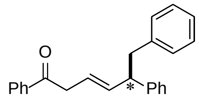

4aa

Racemic sample (**4a**):

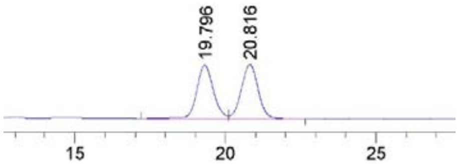

Peak #	RetTime [min]	Type	Width [min]	Area [mAU*s]	Height [mAU]	Area %
1	19.796	BV	0.5895	4443.76709	115.85569	49.3672
2	20.816	VB	0.6037	4557.68066	117.70588	50.6328

Enriched sample (**4aa**):

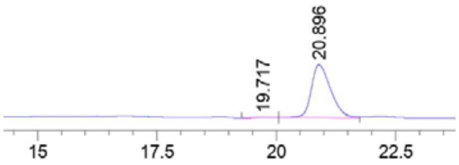

Peak #	RetTime [min]	Type	Width [min]	Area [mAU*s]	Height [mAU]	Area %
1	19.717	BB	0.4670	144.20700	5.11447	0.7735
2	20.896	BV	0.4310	1.84995e4	656.05652	99.2265

11. References

1. K. Lee and D. Y. Oh, *Synthesis*, 1991, 213-214.
2. C. M. M. Santos, A. M. S. Silva, J. A. S. Cavaleiro, A. Lévai and T. Patonay, *E. J. Org. Chem.*, 2007, 2877-2887.
3. D. Trubitsõn, J. Martõnova, K. Erkman, A. Metsala, J. Saame, K. Kõster, I. Järving, I. Leito and T. Kanger, *Synthesis*, 2020, **52**, 1047-1059.
4. L. Lv, D. Zhu, Z. Qiu, J. Li and C.-J. Li, *ACS Catal.*, 2019, **9**, 9199-9205.
